# Crystalline Silaspiropentane and Its Rearrangement to Silacyclobutane

**DOI:** 10.1021/acs.orglett.6c01402

**Published:** 2026-05-25

**Authors:** Wenbang Yang, Benedek Stadler, Mark R. Crimmin

**Affiliations:** Molecular Sciences Research Hub, Imperial College London, 82 Wood Lane, Shepherds Bush, London W12 0BZ, United Kingdom

## Abstract

The first crystallographically characterized silaspiropentane, a molecule containing 4 carbon atoms and 1 silicon atom in two fused three-membered rings, has been isolated. This species was synthesized from the addition of a well-defined silylene compound to methylidene cyclopropane. The silaspiropentane was shown to undergo a thermal rearrangement to the corresponding methylene silacyclobutane in solution. Kinetics show that this is a first-order reaction with little entropic cost to approaching the transition state (Δ*H* ^⧧^ = +26.9 kcal mol^–1^ and Δ*S* ^⧧^ = −2.2 cal K^–1^ mol^–1^). As a point of comparison, the addition of cyclic alkyl amino carbene (cAAC) to methylidene cyclopropane allowed isolation of a spiropentane. While this species did not rearrange to the corresponding methylene cyclobutane, attempts to generate analogues containing aryl substituents led directly to the formation of methylene cyclobutane products as *E* stereoisomers. DFT calculations were used to interrogate the mechanism of these rearrangements and support a concerted process but with distinctly different transition state geometries for the spiropentane and silaspiropentane rearrangement.

## Graphical abstract

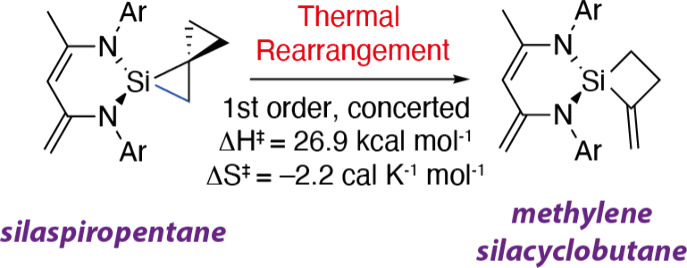

Spiropentane is a molecule that contains five carbon atoms connected in two fused three-membered rings (Figure 1).^1,2^ This functional group has potential applications in drug discovery and materials science. For example, the spiropentyl group is a bioisostere for the phenyl group that allows projection of the structure into three-dimensional space.^3^ Very recently, active pharmaceutical ingredients containing spiropentyl groups have emerged for the treatment of Parkinson’s disease and cystic fibrosis.^4,5^

**Figure 1:**
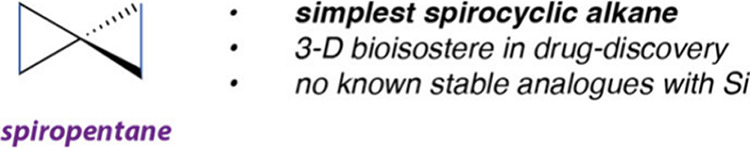
Structure of spiropentane.

Gas-phase electron diffraction studies on spiropentane have demonstrated that this compound contains two different carbon–carbon bond lengths, with those radiating from the central carbon [1.482(1) Å] being shorter than those connecting the peripheral carbon atoms [1.557(3) Å].^6^ While isolable, above 350 °C, spiropentane undergoes a thermally induced rearrangement to methylene cyclobutane, a process that occurs in competition with fragmentation to ethene and propan-1,2-diene.^7,8^ The formation of these products has been explained by invoking a diradical intermediate formed from the reversible homolytic cleavage of the peripheral carbon–carbon bond in spiropentane, with rearrangement to methylene cyclobutane occurring by a 1,2-alkyl shift and ring closure through radical recombination.^9–13^ The rearrangement is thought to exist on the boundary between a concerted and stepwise process, with unstable biradical intermediates leading to the loss of stereocontrol.^14–18^

Analogues of spiropentane that contain one or more silicon atoms in place of carbon have not been reported. Substitution of carbon for silicon is increasingly investigated in drug discovery.^19,20^ Hence, synthetic access to silicon-containing analogues of spiropentane could create new opportunities to study novel fragments for new potential applications. Tentative evidence has been put forward for the existence of a *D*_2*d*_-symmetric silaspiropentane isomer formed in an argon matrix at −263 °C by co-condensation of silicon atoms with ethylene.^21,22^ A more complex molecule containing silacyclopropane flanked by two cyclopropane rings has been isolated and crystallographically characterized,^23^ but the silaspiropentane group remains unknown.

In this paper, we report the synthesis of a silicon-containing analogue of spiropentane, silaspiropentane through the addition of a well-defined silylene compound to methylidene cyclopropane. We describe the first crystallographically characterized silaspiropentane. At elevated temperatures, this compound rearranges to methylene silacyclobutane. Kinetics and DFT calculations support a concerted pathway for the silaspiropentane to methylene silacyclobutane rearrangement, complementing prior studies on the high-temperature rearrangement of spiropentane itself.

The reaction between silylene **1a** and methylidene cyclopropane at 60 °C for 1 h yielded silaspiropentane **2** (Figure 2a). **2** is the first known silaspiropentane to be isolated. The six protons of silaspiropentane appear as a series of upfield resonances in the ^1^H NMR spectrum between δ_H_ of 1.05 and 0.06 ppm, with the four resonances of the cyclopropane moiety appearing as doublets of doublets of doublets. Prior work has demonstrated that silylenes react with alkenes to form cyclopropane analogues by [2 + 2] cycloadditions.^24,25^

**Figure 2:**
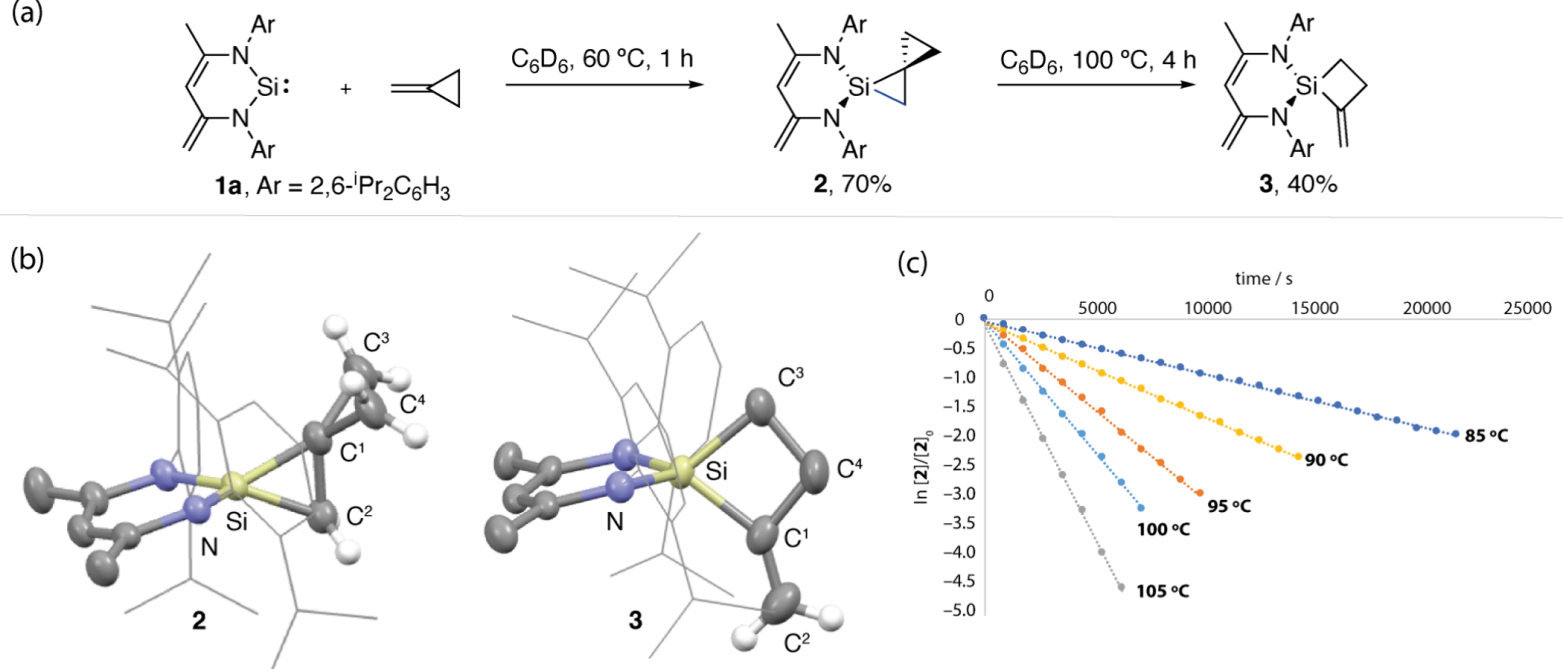
(a) Formation of silaspiropentane **2** and its rearrangement to methylene silacyclobutane **3**. (b) Crystal structures of **2** and **3**, with most hydrogen atoms omitted for clarity. (c) Plot of ln­[**2**]/[**2**]_0_ vs time at various temperatures used in Eyring analysis for the isomerisation of **2** to **3**.

In the ^13^C­{^1^H} NMR spectrum, the methylene carbons of silaspiropentane resonate at δ_C_ = 14.1, 12.9, and 12.7 ppm, with quaternary carbon observed at δ_C_ = 1.7 ppm. A single resonance at δ_Si_ = −39.6 ppm is observed in the ^29^Si NMR spectrum, corresponding to an upfield shift of Δδ_Si_ = 128.0 ppm relative to parent silylene **1a**. The crystal structure of **2** contains the first structural characterized silaspiropentane group (Figure 2b). There are two distinct bond lengths from carbon to silicon, with that to the central carbon atom C^1^–Si of 1.7912(14) Å shorter than the peripheral C^2^–Si bond of 1.8389(15) Å. The C^1^–C^3^, C^1^–C^4^, and C^3^–C^4^ bond lengths take values of 1.493(2), 1.499(2), and 1.565(2) Å, respectively.

Heating a toluene-*d*_8_ solution of **2** to 100 °C resulted in conversion to methylene silacyclobutane **3** (Figure 2a). The rearrangement of silaspiropentane **2** to methylene silacyclobutane **3** is noteworthy not only because it has never been observed before but also as it proceeds to selectively form a single isomer of the product. **3** demonstrates a singlet resonance at δ_Si_ = −21.9 ppm in the ^29^Si NMR spectrum along with characteristic alkenic protons between δ_H_ = 5.43 and 5.37 ppm in the ^1^H NMR spectrum. Heating of **3** to 150 °C for extended periods resulted in no further reaction, with no evidence for fragmentation to silaallene or ethene.^22^ The solid-state structure of **3** shows a methylene silacyclobutane structure with two independent molecules within the asymmetric unit. The Si–C bonds range between 1.872(3) and 1.894(2) Å. The internal C–C bonds of the silacyclobutane ring of 1.529(4)–1.558(4) Å are long in comparison to the exocyclic C=C bond length of 1.313(4)–1.318(4) Å. The bond angles within cyclobutane are expectedly acute, with C^1^–Si–C^3^, Si–C^3^–C^4^, and Si–C^1^–C^4^ angles of 78.2(1)–78.7(1)°, 89.9(2)°, and 90.5(2)–90.7(2)°, respectively. To the best of our knowledge, only one other structurally similar silacyclobutane with an exocyclic alkene has been reported, in this case as part of an *ortho*-fused ring system.^26^

Kinetic analysis was conducted. Plotting ln­[**2**] against time showed a linear relationship indicating that the reaction is first-order in **2** and highly likely to be an intramolecular process. Five rate constants were determined in the temperature range of 85–105 °C at 5 °C intervals, and a plot of ln­(*k*_obs_) against 1/*T* allowed calculation of the thermodynamic parameters using the Eyring equation. The activation parameters for the reaction were found to be Δ*H* ^⧧^ = +26.9 kcal mol^–1^ and Δ*S* ^⧧^ = −2.2 cal K^–1^ mol^–1^, with Δ*G* ^⧧^_298 K_ = 27.6 kcal mol^–1^. The small and negative activation entropy is consistent with minimal reorganization of the structure at the transition state. For comparison, the rearrangement of spiropentane to methylene cyclobutane has also been recorded as a first-order process but with a much higher activation energy of 57.6 kcal mol^–1^.^7^ Substitution of spiropentane by an isopropenyl group has been estimated to lower this value to around 41.0 kcal mol^–1^.^10^

As a point of mechanistic comparison, we sought a synthetic approach to analogous spiropentane compounds. Attempts to react *N*-heterocyclic carbene IMes [1,3-bis­(2,6-diisopropylphenyl)-4,5-dimethyl-1*H*-imidazol-3-ium-2-ide] with methylene cyclopropane only resulted in the recovery of the starting material. A more nucleophilic carbene analogue was considered. Cyclic alkyl amino carbene (cAAC) **1b** reacted with methylidene cyclopropane to form spiropentane analogue **4** after 1 h at 60 °C in C_6_D_6_ (Figure 3a). Prior work has shown that singlet methylene ^1^[CH_2_] reacts with methylidene cyclopropane to form a mixture containing spiropentane, methylene cyclobutane, substituted cyclopropane isomers, ethene, propan-1,2-diene, and isoprene.^7,27^ In the current case, no side products are observed spectroscopically. **4** was isolated and spectroscopically characterized. The crystal structure of **4** contains a disordered spiropentane motif modeled in two orientations (Figure 3b). The four C–C bond lengths to the central carbon atom take values between 1.481(2) to 1.484(2) Å, with those of the peripheral C–C bonds being 1.521(2) and 1.552(2) Å, with the longer distance incorporating the quaternary carbon atom derived from the cAAC reagent. While **4** did not undergo a clear reaction to form the expected methylene cyclobutane **5a**, **1b** reacts with a range of benzylidene cyclopropanes to yield the corresponding *E*-isomeric products of the spiropentane rearrangement **5b**–**5f** as air-stable crystalline solids in 40–70% yield (Figure 3b).^28^

**Figure 3:**
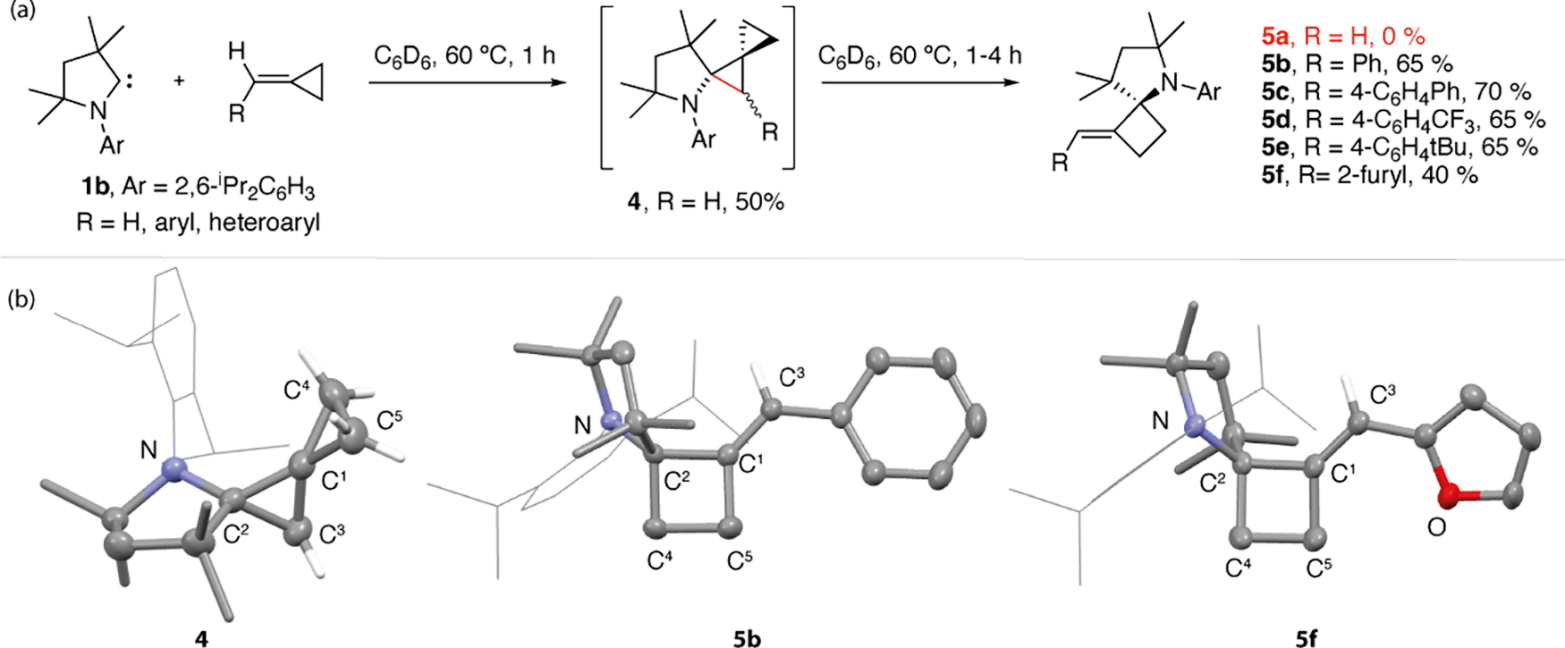
(a) Reaction of carbene analogue **1b** with methylene cyclopropane to form spiropentane **4** and benzylidene cyclopropane to form **5b**–**5f**. (b) Crystal structure of **4**, **5b**, and **5f**, with most hydrogen atoms omitted for clarity.

Although the comparison of **2** and **4** is imperfect both electronically and sterically, as they are not isostructural, consideration of the mechanisms of the rearrangements of these species is still informative.

Density functional theory (DFT) calculations were performed on the rearrangement of both **2** and **4**. Geometry optimizations and frequency analyses were conducted with the BP86-D3 functional considering solvent effects (CPCM, benzene, ε = 2.2706). Single-point electronic energies are reported with the def2-TZVPP basis set, corrected to the experimental temperature and concentration using GoodVibes. A viable closed-shell pathway was found for both reactions. The formation of silaspiropentane **2** was calculated as an exergonic process (Δ*G*_373 K_^°^ = −3.7 kcal mol^–1^) that occurs through the expected [2 + 2] cycloaddition transition state **TS-1** (Δ*G*_373 K_^⧧^ = 24.0 kcal mol^–1^). The subsequent rearrangement of **2** to **3** was calculated to be feasible through concerted transition state **TS-2** (Δ*G*_373 K_^⧧^ = 26.2 kcal mol^–1^) in an exergonic step (Δ*G*_373 K_^°^ = −30.8 kcal mol^–1^) reminiscent of α-alkyl migration, known to occur at metal centers (Figure 4a).^29,30^ There is excellent agreement of the calculated activation energy with the experimentally determined activation parameters with Δ*G*_373 K_^⧧^(calc.) = 26.2 kcal mol^–1^ and Δ*G*_373 K_^⧧^(exp.) = 27.7 kcal mol^–1^.

**Figure 4:**
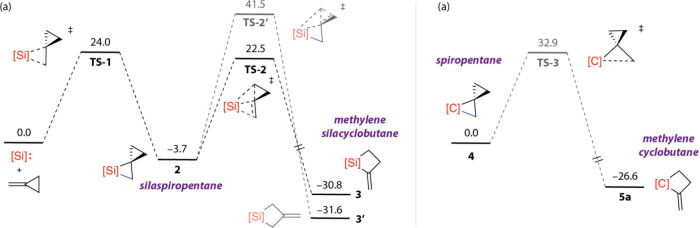
Calculated concerted pathways for the formation and isomerization of (a) silaspiropentane **2** to methylene silacyclobutane **3** and (b) spiropentane **4** to methylene cyclobutane **5a** and G16: BP86-D3/def2-TZVPP/CPCM­(benzene)//BP86-D3/def2-SVP­(C,H)/def2-TZVPP­(N,Si)/CPCM­(benzene). Gibbs energy values are in kcal mol^–1^. The temperature (333 K for **4** to **5a** and 373 K for **2** to **3**) and concentration correction (0.14 M) were performed on the reaction pathway using GoodVibes. Silylene and cAAC fragments were represented as [Si] and [C], respectively.

It was expected that the peripheral carbon–silicon bond of **2** would be a more likely site of initial reactivity based on known C–C and C–Si bond dissociation energies.^31^ The alternative, cleavage of the peripheral carbon–carbon bond, was also considered computationally. While this pathway leads to a more thermodynamically stable isomer of product **3′** (Δ*G*_373 K_^°^ = −31.6 kcal mol^–1^), it was found to proceed through a much higher energy transition state **TS-2′** (Δ*G*_373 K_^⧧^ = 41.5 kcal mol^–1^ and ΔΔ*G*_373 K_^⧧^ = 19.0 kcal mol^–1^) and is not expected to be competitive with the experimentally observed pathway. For comparison, the pathway from spiropentane **4** to the corresponding methylene cyclobutane **5a** was found to occur through a concerted transition state **TS-3** (Δ*G*_373 K_^⧧^ = 32.9 kcal mol^–1^). The high activation energy is consistent with the lack of experimental evidence for product formation in this reaction (Figure 4b).

Although viable concerted pathways can be calculated for both the silaspiropentane and spiropentane rearrangements, a comparison of the key transition states suggests that these pathways fundamentally differ (Figure 5). The silaspiropentane motif is largely intact at the transition state geometry of **TS-2** but distorted to allow bond breaking and making at an apparently hypervalent silicon center. The breaking Si- - -C^2^ bond is 1.89 Å with the Si–C^1^–C^2^ angle of 66.7°, remaining close to that expected of the starting material. The geometry of the central carbon atom of silaspiropentane is distorted away from tetrahedral, deviating toward a seesaw-type geometry. This distortion allows the silacyclobutane motif to begin to form as the Si- - -C^3^ distance of 2.04 Å begins to approach the sum of the covalent radii of Si and C of 1.81 Å.^32^ NBO analysis shows that the Si center is electropositive (+1.95) with negative charge buildup across C^1^ (−0.24), C^2^ (−0.89), and C^3^ (−0.64). As **TS-2**, such can be conceptualized in terms of rearrangement of an anionic hydrocarbon fragment in the coordination sphere of silicon.

**Figure 5:**
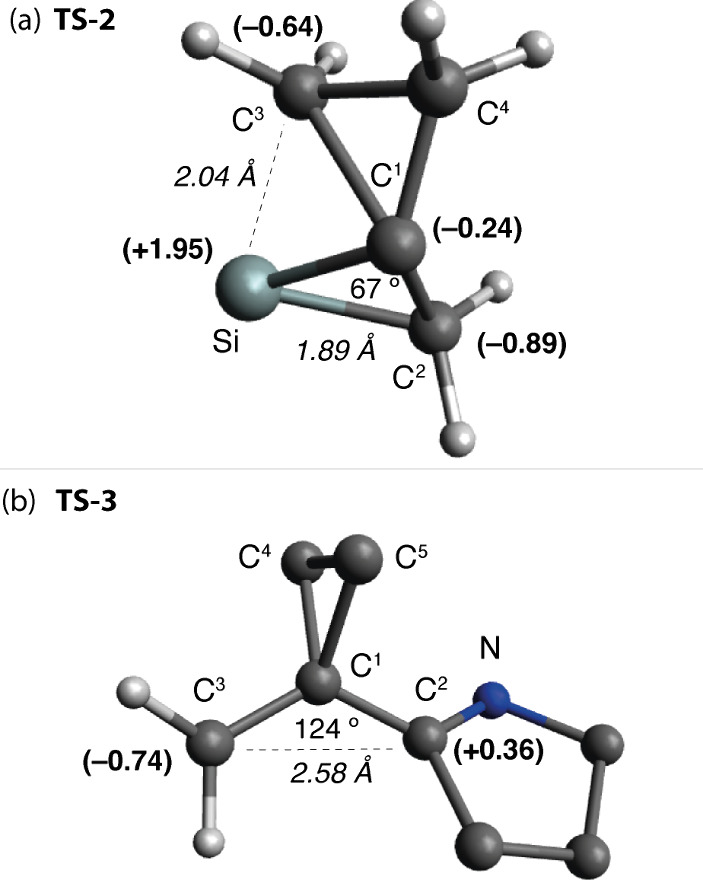
Comparison of transition states of the silaspiropentane and spiropentane rearrangement. G16: BP86-D3/def2-TZVPP/CPCM­(benzene)//BP86-D3/def2-SVP­(C,H)/def2-TZVPP­(N,Si)/CPCM­(benzene).

In contrast, for spiropentane, **TS-3** has an extremely stretched C^2^- - -C^3^ distance of 2.58 Å in the breaking carbon–carbon bond along with an obtuse C^2^–C^1^–C^3^ angle of 124°. The trigonal C^2^ and C^3^ centers are nearly coplanar with the carbon skeleton. Hofmann has conducted extended Hückel calculations on trimethylene (CH_2_CH_2_CH_2_) and concluded that a closely related structure is a singlet and an intermediate in ring closure and ring opening of cyclopropane.^33^ NBO analysis is consistent with consideration of **TS-3** as a 1,3-dipolar structure as a positive charge builds up on C^2^ (+0.36) and a negative charge builds up on C^3^ (−0.74). This dipolar nature of the transition state is likely induced by the presence of the cAAC moiety.

In summary, we report the reaction of a stable silylene with methylidene cyclopropane to form the first isolated and structurally characterized silaspiropentane. At high temperatures, this species undergoes a thermal rearrangement to the corresponding methylene silacyclobutane. The reaction follows first-order kinetics and proceeds with activation parameters of Δ*H* ^⧧^ = +26.9 kcal mol^–1^ and Δ*S* ^⧧^ = −2.2 cal K^–1^ mol^–1^. DFT calculations suggest that the silaspiropentane to methylene silacyclobutane rearrangement proceeds through a concerted mechanism. As a point of comparison, the spiropentane to methylene cyclobutane rearrangement was also studied. We conclude that, while both processes are related, they show fundamentally different transition state geometries. Silaspiropentane reported here is the first of its kind and opens the door to potential applications of this new group as a three-dimensional scaffold. Moreover, our findings expand the understanding of non-metallic main group centers in C–C σ-bond activation, providing a mechanistic alternative to Büchner ring expansion.

## Supplementary Material





## Data Availability

The data underlying this study are available in the published article and its .

## Notes

Experimental procedures, details of the kinetic studies, crystal structures of compounds **S1**, **S2**, and **S3**, calculations, and spectroscopic data (PDF)
Cartesian coordinates for DFT-calculated stationary points (XYZ)

## References

[1] Gustavson G. (1896). Ueber aethylidentrimethylen. J. Prakt. Chem..

[2] Applequist D. E., Fanta G. F., Henrikson B. W. (1958). Chemistry
of spiropentane. I. An
improved synthesis of spiropentane. J. Org.
Chem..

[3] Sun Q., Belting J.-N., Hauda J., Tymann D., Antoni P. W., Goddard R., Hansmann M. M. (2025). Spiro-C­(sp^3^)-atom transfer:
Creating rigid three-dimensional structures with Ph_2_SCN_2_. Science.

[4] Candito D. A., Simov V., Gulati A., Kattar S., Chau R. W., Lapointe B. T., Methot J. L., DeMong D. E., Graham T. H., Kurukulasuriya R., Keylor M. H., Tong L., Morriello G. J., Acton J. J., Pio B., Liu W., Scott J. D., Ardolino M. J., Martinot T. A., Maddess M. L., Yan X., Gunaydin H., Palte R. L., McMinn S. E., Nogle L., Yu H., Minnihan E. C., Lesburg C. A., Liu P., Su J., Hegde L. G., Moy L. Y., Woodhouse J. D., Faltus R., Xiong T., Ciaccio P., Piesvaux J. A., Otte K. M., Kennedy M. E., Bennett D. J., DiMauro E. F., Fell M. J., Neelamkavil S., Wood H. B., Fuller P. H., Ellis J. M. (2022). Discovery and Optimization of Potent, Selective, and
Brain-Penetrant 1-Heteroaryl-1*H*-Indazole LRRK2 Kinase
Inhibitors for the Treatment of Parkinson’s Disease. J. Med. Chem..

[5] Wang Y., Yang F., Wang B., Xie L., Chen W. (2025). New FDA drug
approvals for 2024: Synthesis and clinical application. Eur. J. Med. Chem..

[6] Sandwisch J. W., Erickson B. A., Hedberg K., Nibler J. W. (2017). Combined
electron-diffraction
and spectroscopic determination of the structure of spiropentane,
C_5_H_8_. J. Phys. Chem. A.

[7] Doering W. V. E., Gilbert J. C., Leermakers P. A. (1968). Symmetrical
distribution of energy
in initially unsymmetrically excited products: Reaction of dideuteriodiazomethane
with allene, methylenecyclopropane and vinylcyclopropane. Tetrahedron.

[8] Flowers M. C., Gibbons A. R. (1971). Kinetics of the thermal gas phase
reactions of methylspiro[2.2]­pentane. J. Chem.
Soc. B: Phys. Org..

[9] Doering W. V. E., Gilbert J. C. (1966). Degenerate rearrangement
of methylenecyclobutane. Tetrahedron.

[10] Gajewski J. J. (1967). Alkyl shifts
in diradicals: thermal isomerization of isopropenylspiropentane. Chem. Commun..

[11] Gillbert J. C. (1969). Geometric
isomerisation of spiropentane. Tetrahedron.

[12] Gajewski J. J., Burka L. T. (1971). Stereochemistry
of and alteration in the mechanism
of the spiropentane to methylenecycbutane thermal rearrangement by
polar substituents. J. Am. Chem. Soc..

[13] Gajewski J. J., Burka L. T. (1972). Alkyl shifts in
thermolyses. V. Thermal epimerization
of the 1,4-dimethylspiropentanes. J. Am. Chem.
Soc..

[14] Gajewski J. J., Burka L. T. (1972). Alkyl shifts in thermolyses. IV. Carbethoxyspiropentane-carbethoxymethylenecyclobutane
isomerization. Evidence for orbital symmetry control and an intermediate. J. Am. Chem. Soc..

[15] Gajewski J. J., Burka L. T. (1972). Alkyl shifts in thermolyses. VII. Stereochemistry and
kinetics of the carbethoxyspiropentane to carbethoxymethylenecyclobutane
rearrangement. Evidence for concertion and an intermediate. J. Am. Chem. Soc..

[16] Gajewski J. J., Weber R. J., Chang M. J. (1979). Dominant double rotation in the thermally
induced 1,2,4-trimethylspiropentane geometric isomerization. J. Am. Chem. Soc..

[17] Gajewski J. J., Chang M. J. (1980). Dominant disrotatory double rotation in the thermally
induced 1,2-dimethylspiropentane geometric isomerization. J. Am. Chem. Soc..

[18] Johnson W. T. G., Hrovat D. A., Borden W. T. (1999). Ab Initio Calculations on Spiropentane
Stereomutations Lead to a Reinterpretation of the Experimental Results. J. Am. Chem. Soc..

[19] Panayides J. L., Riley D. L., Hasenmaile F., van Otterlo W. A. L. (2024). The
role of silicon in drug discovery: a review. RSC Med. Chem..

[20] Lazareva N. F., Lazarev I. M. (2015). Drug design based on the carbon/silicon switch strategy. Russ. Chem. Bull..

[21] Maier G., Reisenauer H. P., Egenolf H. (1998). Reaction of Silicon
Atoms with Acetylene
and Ethylene: Generation and Matrix-Spectroscopic Identification of
C_2_H_2_Si and C_2_H_4_Si Isomers. Eur. J. Org. Chem..

[22] Yildiz C. B., Azizoglu A. (2016). A Mechanistic investigation
on the formation of silaspiropentane:
A theoretical study. J. Mol. Model.

[23] Delker G. L., Wang Y., Stucky G. D., Lambert R. L., Haas C. K., Seyferth D. (1976). Molecular structure
and bonding of a silacyclopropane,
dimethyldispiro­[bicyclo[4.1.0]­heptane-7,2′-silacyclopropane-3′,7″-bicyclo[4.1.0]­heptane]. J. Am. Chem. Soc..

[24] Lips F., Mansikkamäki A., Fettinger J. C., Tuononen H. M., Power P. P. (2014). Reactions
of Alkenes and Alkynes with an Acyclic Silylene and Heavier Tetrylenes
under Ambient Conditions. Organometallics.

[25] Ishida S., Iwamoto T., Kira M. (2011). Addition of
a Stable Dialkylsilylene
to Carbon–Carbon Unsaturated Bonds. Heteroat.
Chem..

[26] Milnes K.
K., Jennings M. C., Baines K. M. (2006). Addition of Cyclopropyl Alkynes to
a Brook Silene: Definitive Evidence for a Biradical Intermediate. J. Am. Chem. Soc..

[27] Frey H. M., Jackson G. E., Smith R. A., Walsh R. (1975). Reaction of singlet
methylene with methylenecyclopropane. Part 1. Evidence for multistep
collisional deactivation of chemically activated spiropentane. J. Chem. Soc., Faraday Trans.

[28] Gajewski J. J., Burka L. T. (1972). Alkyl shifts in
thermolyses. VI. Synthesis and characterization of the 2,4- and 4,5-dimethyl-1-carbethoxyspiropentanes
and the 2-methyl-3-ethylidene-1-carbethoxycyclopropanes. J. Am. Chem. Soc..

[29] Kong R. Y., Crimmin M. R. (2020). Activation and Functionalization of C–C σ
Bonds of Alkylidene Cyclopropanes at Main Group Centers. J. Am. Chem. Soc..

[30] Parr J. M., Crimmin M. R. (2023). Carbon–carbon bond activation by Mg, Al, and
Zn complexes. Chem. Sci..

[31] Grunenberg J. (2001). Intrinsic
Bond Strengths of Multiple C–C, Si–Si, and C–Si
Bonds. Angew. Chem., Int. Ed..

[32] Pyykkö P., Atsumi M. (2009). Molecular Single-Bond
Covalent Radii for Elements 1–118. Chem.
- Eur. J..

[33] Hoffmann R. (1968). Trimethylene
and the addition of methylene to ethylene. J.
Am. Chem. Soc..

