# Supplementary material for: Crystalline Silaspiropentane and Its Rearrangement to Silacyclobutane
Source: Org Lett. 2026 May 25;28(22):6884–8. doi: 10.1021/acs.orglett.6c01402 (PMC13247965; doi:10.1021/acs.orglett.6c01402)
Supplement: Supplementary file 1 [file ol6c01402_si_001.pdf]

Supporting Information for:

A Crystalline Silaspiropentane and its Rearrangement to a Silacyclobutane

Wenbang Yang[a], Benedek Stadler[a] and Mark R Crimmin*[a]

Corresponding author email: m.crimmin@imperial.ac.uk

[a] Molecular Sciences Research Hub, Imperial College London, 82 Wood Lane, Shepherds Bush, W12 0BZ, UK.

Table of Contents

1) General Experimental **S3**

2) Experimental Methods **S4**

2.1) Preparation of Compounds S4

3) Additional Reactions **S16**

4) Competition reaction **S18**

5) Single Crystal X-ray Diffraction Data **S19**

6) DFT Studies **S28**

6.1) Computational methods S28

6.2) Calculated Reaction Pathway and Optimised Structures S29

6.3) NBO Analysis S34

7) Kinetic Analysis **S43**

7.1) Kinetics Experiments S43

8) NMR Spectroscopy **S46**

8.1) NMR Spectra of Isolated products S46

9) XYZ Coordinates **S58**

10) References **S87**

1) General experimental

Standard Schlenk line and glovebox techniques were used for all manipulations under an inert atmosphere of dinitrogen or argon unless otherwise stated. NMR scale reactions were performed in J. Young NMR tubes. A MBraun Labmaster glovebox was employed, operating at <0.1 ppm O_2 and <0.1 ppm H_2O.

Instruments: ^{1}H, ^{13}C NMR spectra were recorded on BRUKER 400 MHz or 500 MHz machines. All peaks are referenced against residual solvent and values are quoted in ppm. Data were processed using the MestReNova software. The coupling constants (J) are reported in hertz (Hz). The following abbreviations are used to define multiplicities: s (singlet), d (doublet), t (triplet), q (quadruplet), hept (heptet), dd (doublet of doublets), ddd (doublet of doublets of doublets), dt (doublet of triplets), td (triplet of doublets), m (multiplet).

^{1}H NMR yields were determined by using 1,3,5-trimethoxybenzene as internal standard (δ_H = 3.4, 6.1 ppm in C_6D_6) or ferrocene as internal standard (δ_H = 4.0 ppm in C_6D_6).

Infrared spectra were obtained on a Cary630 spectrometer (located in an MBraun glovebox) from crystalline solids or benzene thin films on an ATR cell.

Purifications were carried out by column chromatography on silica gel (tech grade, 60 Å, 230-400 mesh, 40-63 µm particle size, Sigma-Aldrich).

Chemicals: Solvents were dried over activated alumina from a solvent purification system (SPS) based upon the Grubbs design and de-gassed before use. Glassware was dried for >6 h prior to use at 120 °C. Benzene-d_6 and toluene-d_8 were de-gassed using three freeze-pump-thaw cycles and dried over 3 Å molecular sieves before use. All reagents and chemicals were acquired from Sigma Aldrich (Merck), Fluorochem, or VWR and used without further purification unless specified. ^{Me}CAAC (**1a**)[S1-S2], {DippNC(=CH_2)CH(Me)NDipp}Si, (Dipp = 2,6-di-isopropylphenyl) (**1b**)[S3], {DippNC(=CH_2)CH(Me)NDipp}Ge (**1c**)[S4-S5], (MesNCH=CHNMes}Sn, (Mes = 1,3,5-trimethylbenzene) (**1d**)[S6], alkylidene cyclopropane[S7-S8] and benzylidene cyclobutanes[S7] were prepared by literature procedures.

2) Experimental Methods

2.1 Preparation of Compounds

Preparation of 2

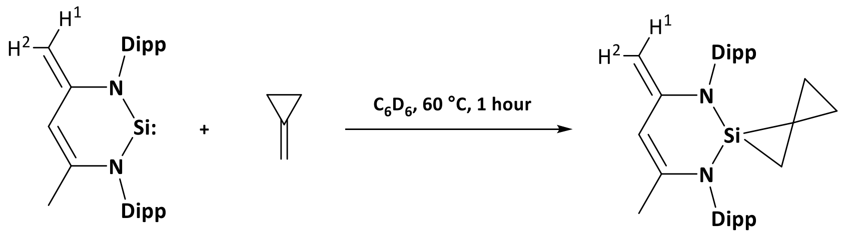

In a N_2 filled glovebox, {DippNC(=CH_2)CH(Me)NDipp}Si (**1a**) (40.0 mg, 0.09 mmol, 1 eq) and methylenecyclopropane (4.9 mg, 0.09 mmol, 1.0 equiv.) were dissolved in C_6D_6 (1.0 mL) and transferred to a J-Young NMR tube. Ferrocene in a sealed glass capillary containing C_6D_6 (δ_H = 4.0 ppm) was added as internal standard. The tube was sealed, removed from the glovebox, and an initial ^{1}H NMR spectra recorded. The reaction mixture was heated to 60 °C in a silicone oil bath for 1 hour. Quantitative ^{1}H NMR spectroscopy revealed that product (**2**) was completely formed (NMR yield = 80 %). The tube was returned to the glovebox; the reaction mixture was diluted with *n*-hexane (1 mL) and the solution decanted into a scintillation vial. The volatiles removed *in vacuo* to yield product (**2**) as a colourless oil. **Yield: 31.4 mg,** 0.06 mmol, 70%.

^{1}H NMR (400 MHz, 298 K, C_6D_6): δ 7.25 – 7.21 (m, 1H, ArC***H***), 7.20 – 7.17 (m, 2H, ArC***H***), 7.11 (m, 1H, ArC***H***), 7.01 (m, 2H, ArC***H***), 5.32 (s, 1H, CH_2CC***H***C(CH_3)), 4.05 (s, 1H, s, 1H, NCC$\boldsymbol{H^1}$$H^2$), 3.67 (hept, $^3J_{H-H}$ = 6.7 Hz, 1H, (C***H***(CH_3)(CH_3)), 3.64 (hept, $^3J_{H-H}$ = 6.7 Hz, 1H, (C***H***(CH_3)(CH_3)), 3.61 (hept, $^3J_{H-H}$ = 6.7 Hz, 1H, (C***H***(CH_3)(CH_3)), 3.57 (hept, $^3J_{H-H}$ = 6.7 Hz, 1H, (C***H***(CH_3)(CH_3)), 3.46 (s, 1H, s, 1H, NCC$H^1$$\boldsymbol{H^2}$), 1.51 (s, 3H, NCC$\boldsymbol{H_3}$), 1.40 (d, $^3J_{H-H}$ = 6.8 Hz, 3H, (CH(CH_3)(C$\boldsymbol{H_3}$)), 1.35 (d, $^3J_{H-H}$ = 6.8 Hz, 3H, (CH(CH_3)(C$\boldsymbol{H_3}$)), 1.28 (d, $^3J_{H-H}$ = 6.9 Hz, 3H, (CH(CH_3)(C$\boldsymbol{H_3}$)), 1.27 (d, $^3J_{H-H}$ = 6.9 Hz, 3H, (CH(CH_3)(C$\boldsymbol{H_3}$)), 1.20 (d, $^3J_{H-H}$ = 6.9 Hz, 3H, (CH(CH_3)(C$\boldsymbol{H_3}$)), 1.19 (d, $^3J_{H-H}$ = 6.9 Hz, 3H, (CH(CH_3)(C$\boldsymbol{H_3}$)), 1.10 (d, $^3J_{H-H}$ = 6.9 Hz, 3H, (CH(CH_3)(C$\boldsymbol{H_3}$)), 1.08 (d, $^3J_{H-H}$ = 6.9 Hz, 3H, (CH(CH_3)(C$\boldsymbol{H_3}$)), 1.05 (m, 2H, SiCC$\boldsymbol{H_2}$), 0.58 (ddd, J_{H-H} = 8.4, 5.8, 3.7 Hz, 1H, CC$\boldsymbol{H^3}$$H^4CH^5$$H^6$), 0.32 (ddd, J_{H-H} = 9.2, 5.8, 3.7 Hz, 1H, CC$H^3$$H^4C\boldsymbol{H^5}$$H^6$), 0.14 (ddd, J_{H-H} = 9.0, 5.8, 3.5 Hz, 1H, CC$H^3$$\boldsymbol{H^4}CH^5$$H^6$), 0.06 (ddd, J_{H-H} = 8.4, 5.8, 3.5 Hz, 1H, CC$H^3$$H^4CH^5$$\boldsymbol{H^6}$).

^{13}C{^{1}H} NMR (101 MHz, 298 K, C_6D_6): δ 148.6 (N***C***CH_2), 148.3 (N***C***(CH_3)), 148.2 (Ar***C***), 148.1 (Ar***C***), 147.6 (Ar***C***), 142.1 (Ar***C***), 136.3 (Ar***C***), 135.6 (Ar***C***), 128.0 (Ar***C***H), 127.8 (Ar***C***H), 124.9 (Ar***C***H), 124.2 (Ar***C***H), 123.8 (Ar***C***H), 123.7 (Ar***C***H), 103.2 (CH_2C***C***HC(CH_3)), 88.0 (NC***C***H_2), 28.9 (***C***H(CH_3)(CH_3)), 28.4 (***C***H(CH_3)(CH_3)), 28.1 (***C***H(CH_3)(CH_3)), 27.2 (***C***H(CH_3)(CH_3)), 26.0 (CH(CH_3)(***C***H_3)), 25.5 (CH(CH_3)(***C***H_3)), 24.7 (CH(CH_3)(***C***H_3)), 24.6 (CH(CH_3)(***C***H_3)), 24.4 (CH(CH_3)(***C***H_3)), 24.0 (CH(CH_3)(***C***H_3)), 23.8 (CH(CH_3)(***C***H_3)), 23.3 (CH(CH_3)(***C***H_3)), 21.6 (NC(***C***H_3)), 14.1 (SiC***C***H_2), 12.9 (C***C***$H^3$$H^4CH^5$$H^6$), 12.7 (CC$H^3$$H^4$***C***$H^5$$H^6$), 1.7 (***C***C$H^3$$H^4CH^5$$H^6$).

^{29}Si{^{1}H} NMR (99 MHz, 298 K, C_6D_6) δ: -39.6 (s, ***Si***CCH_2).

Preparation of 3

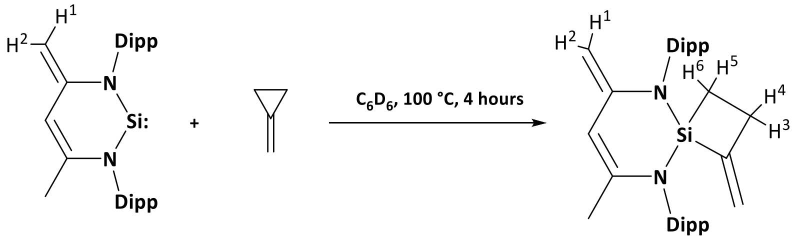

In a N_2 filled glovebox, {DippNC(=CH_2)CH(Me)NDipp}Si (**1a**) (40.0 mg, 0.09 mmol, 1 eq) and methylenecyclopropane (4.9 mg, 0.09 mmol, 1.0 equiv.) were dissolved in C_6D_6 (1.0 mL) and transferred to a J-Young NMR tube. Ferrocene in a sealed glass capillary containing C_6D_6 (δ_H = 4.0 ppm) was added as internal standard. The tube was sealed, removed from the glovebox, and an initial ^{1}H NMR spectra recorded. The reaction mixture was heated to 100 °C in a silicone oil bath for 4 hours. Quantitative ^{1}H NMR spectroscopy revealed that product (**3**) was completely formed (NMR yield = 75 %). The tube was returned to the glovebox; the reaction mixture was diluted with pentane (2 mL) and the solution decanted into a scintillation vial. The volatiles removed *in vacuo* to yield a colourless oil and the crude product was recrystallized in *n*-pentane (0.5 ml) at -35 °C for about 4 days. Colourless crystals (**3**) were successfully obtained. The filtrated crystals were washed with cold n-pentane (3 x 0.5mL) and then dried in vacuo. Yield: 18.0 mg, 0.04 mmol, 40%.

^{1}H NMR (400 MHz, 298 K, C_6D_6): δ 7.27 – 7.23 (m, 1H, ArC***H***), 7.22 – 7.18 (m, 2H, ArC***H***), 7.14 (m, 1H, ArC***H***), 7.06 (ddd, $J_{H\text{-}H}$ = 9.3, 7.7, 1.7 Hz, 2H, ArC***H***), 5.43 – 5.37 (m, 2H, SiC=C$\boldsymbol{H_2}$), 5.32 (s, 1H, CH_2CC***H***C(CH_3)), 3.98 (s, 1H, s, 1H, NCC$\boldsymbol{H^1}$$H^2$), 3.70 (hept, $^3J_{H\text{-}H}$ = 6.7 Hz, 1H, (C***H***(CH_3)(CH_3)), 3.64 (hept, $^3J_{H\text{-}H}$ = 6.7 Hz, 1H, (C***H***(CH_3)(CH_3)), 3.62 (hept, $^3J_{H\text{-}H}$ = 6.7 Hz, 1H, (C***H***(CH_3)(CH_3)), 3.58 (hept, $^3J_{H\text{-}H}$ = 6.7 Hz, 1H, (C***H***(CH_3)(CH_3)), 3.34 (s, 1H, NCCH^1$\boldsymbol{H^2}$), 1.78 – 1.69 (m, 1H, SiCC$\boldsymbol{H^3}$$H^4$), 1.65 – 1.54 (m, 1H, SiCCH^3$\boldsymbol{H^4}$), 1.52 (s, 3H, NCC$\boldsymbol{H_3}$), 1.46 (d, $^3J_{H\text{-}H}$ = 6.7 Hz, 3H, (CH(CH_3)(C$\boldsymbol{H_3}$)), 1.43 (d, $^3J_{H\text{-}H}$ = 6.7 Hz, 3H, (CH(CH_3)(C$\boldsymbol{H_3}$)), 1.31 (d, $^3J_{H\text{-}H}$ = 6.9 Hz, 3H, (CH(CH_3)(C$\boldsymbol{H_3}$)), 1.26 (d, $^3J_{H\text{-}H}$ = 6.7 Hz, 3H, (CH(CH_3)(C$\boldsymbol{H_3}$)), 1.25 (d, $^3J_{H\text{-}H}$ = 6.7 Hz, 3H, (CH(CH_3)(C$\boldsymbol{H_3}$)), 1.24 (d, $^3J_{H\text{-}H}$ = 6.7 Hz, 3H, (CH(CH_3)(C$\boldsymbol{H_3}$)), 1.22 (d, $^3J_{H\text{-}H}$ = 6.7 Hz, 3H, (CH(CH_3)(C$\boldsymbol{H_3}$)), 1.13 (d, $^3J_{H\text{-}H}$ = 6.7 Hz, 3H, (CH(CH_3)(C$\boldsymbol{H_3}$)), 1.11 – 1.04 (m, 1H, SiC$\boldsymbol{H^5}$$H^6$CH^{3}H^4), 0.93 – 0.86 (m, 1H, SiCH^5$\boldsymbol{H^6}$CH^{3}H^4).

^{13}C{^{1}H} NMR (101 MHz, 298 K, C_6D_6): δ 160.3 (N***C***CH_2), 149.1 (N***C***(CH_3)), 148.7 (Ar***C***), 148.6 (Ar***C***), 147.6 (Ar***C***), 147.7 (Ar***C***), 140.7 (Si***C***CH_2), 136.6 (Ar***C***), 135.8 (Ar***C***), 127.9 (ArCH), 125.0 (ArCH), 124.5 (Ar***C***H), 124.2 (Ar***C***H), 123.9 (Ar***C***H), 123.7 (Ar***C***H), 104.1 (CH_2C***C***HC(CH_3)), 86.3 (NC***C***H^{1}H^2), 28.8 (***C***H(CH_3)(CH_3)), 28.7 (***C***H(CH_3)(CH_3)), 27.9 (***C***H(CH_3)(CH_3)), 27.5 (***C***H(CH_3)(CH_3)), 27.5 (SiCH^{5}H^6***C***H^{3}H^4), 26.7 (CH(CH_3)(***C***H$_3$)), 26.5 (CH(CH_3)(***C***H$_3$)), 26.2 (CH(CH_3)(***C***H$_3$)), 25.2 (CH(CH_3)(***C***H$_3$)), 25.0 (CH(CH_3)(***C***H$_3$)), 24.8 (CH(CH_3)(***C***H$_3$)), 24.3 (CH(CH_3)(***C***H$_3$)), 23.5 (CH(CH_3)(***C***H$_3$)), 21.8 (NC(***C***H$_3$)), 14.7 (Si***C***H^{5}H^{6}CH^{3}H^4).

^{29}Si{^{1}H} NMR (99 MHz, 298 K, C_6D_6) δ: -21.9 (s, ***Si***CH^{5}H^{6}CH^{3}H^4).

Anal. Calc. ($C_{33}H_{46}N_2Si$): C, 79.46; H, 9.30; N, 5.62. Found: C, 79.24; H, 9.06; N, 5.31.

General procedure A

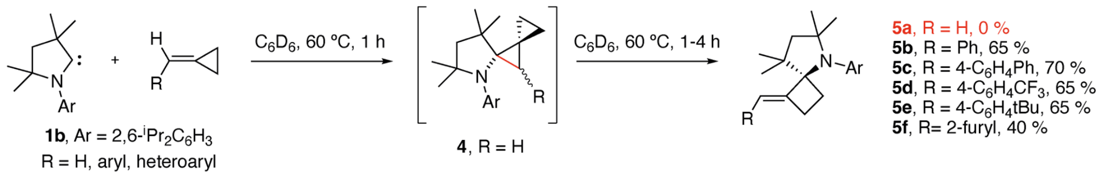

Figure S1. (a) Reaction of carbene analogue **1b** with methylene cyclopropane to form spiropentane **4** and benzylidene cyclopropane to form **5a-f**.

In a N_2 filled glovebox, ^{Me}CAAC (**1b**, 1.0 equiv) and alkylidene cyclopropane (1.0 equiv) were dissolved in C_6D_6 (1.0 mL) and transferred to a J Young NMR tube. A sealed glass capillary containing a solution of ferrocene in C_6D_6 (δ_H = 4.0 ppm) was added as internal standard, and the reaction mixture was analysed by quantitative ^{1}H-NMR spectroscopy. The tube was sealed, removed from the glovebox, and an initial ^{1}H NMR spectra recorded. The tube was heated to 60 °C in a silicone oil bath. After 1-4 hours, complete consumption of the alkylidene cyclopropane and formation of product was observed by ^{1}H NMR spectroscopy. The reaction mixture was diluted with benzene (1 mL) and the solution decanted into a scintillation vial. The volatiles removed *in vacuo* to yield a colourless oil as crude product. The crude was further purified by flash column chromatography under air.

Preparation of 4

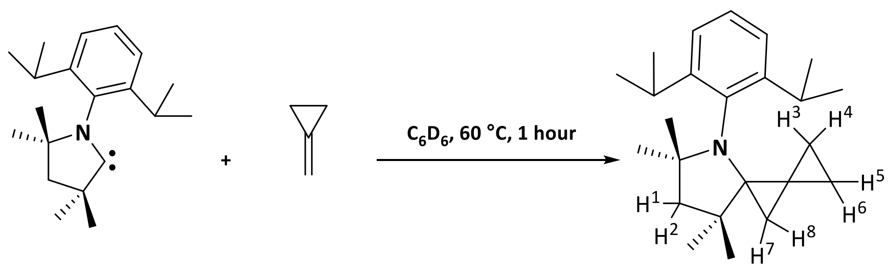

In a N_2 filled glovebox, **1b** (40.0 mg, 0.14 mmol, 1.0 equiv) and methylenecyclopropane (7.6 mg, 0.14 mmol, 1.0 equiv.) were dissolved in C_6D_6 (1.0 mL) and transferred to a J-Young NMR tube. Ferrocene in a sealed glass capillary containing C_6D_6 (δ_H = 4.0 ppm) was added as internal standard. The tube was sealed, removed from the glovebox, and an initial ^1H NMR spectra recorded. The tube was heated to 60 °C in a silicone oil bath for 1 hour. Quantitative ^1H NMR spectroscopy revealed that product (**4**) was completely formed (NMR yield = 70 %). The tube was returned to the glovebox; the reaction mixture was diluted with hexane (1 mL) and decanted into a scintillation vial. The volatiles were removed *in vacuo* to yield a colourless oil which was then recrystallized from *n*-hexane (0.5 ml) at -35 °C for over 4 days. Colourless crystals (**4**) were successfully obtained. The filtrated crystals were washed with cold n-pentane (3 x 0.5mL) and then dried in vacuo. Yield: 23.8 mg, 0.07 mmol, 50%.

^{1}H NMR (400 MHz, 298 K, C_6D_6): δ 7.23 (m, 1H, ArC***H***), 7.18 (m, 2H, ArC***H***), 3.76 (hept, $^3J_{H-H}$ = 6.8 Hz, 1H, C***H***$(CH_3)(CH_3)$), 3.63 (hept, $^3J_{H-H}$ = 6.8 Hz, 1H, C***H***$(CH_3)(CH_3)$), 2.03 (d, $^2J_{H-H}$ = 2.0 Hz, 2H, $(CH_3)(CH_3)C\boldsymbol{CH^1H^2}C(CH_3)(CH_3)$), 1.34 (d, $^3J_{H-H}$ = 6.9 Hz, 3H, $CH(CH_3)(C\boldsymbol{H_3})$), 1.33 (s, 3H, $(C\boldsymbol{H_3})(CH_3)CCH^1H^2C(CH_3)(CH_3)$), 1.33 (s, 3H, $(CH_3)(C\boldsymbol{H_3})CCH^1H^2C(CH_3)(CH_3)$), 1.32 (d, $^3J_{H-H}$ = 6.9 Hz, 3H, $CH(CH_3)(C\boldsymbol{H_3})$), 1.31 (d, $^3J_{H-H}$ = 6.9 Hz, 3H, $CH(CH_3)(C\boldsymbol{H_3})$), 1.30 (s, 3H, $(CH_3)(CH_3)CH^1H^2C(C\boldsymbol{H_3})(CH_3)$), 1.24 (s, 3H, $(CH_3)(CH_3)CCH^1H^2C(CH_3)(C\boldsymbol{H_3})$), 1.20 (d, $^3J_{H-H}$ = 6.8 Hz, 3H, $CH(CH_3)(C\boldsymbol{H_3})$), 1.12 (ddd, J_{H-H} = 9.2, 5.5, 4.1 Hz, 1H,$C\boldsymbol{CH^3}H^4CH^5H^6$), 0.98 (d, $^2J_{H-H}$ = 4.7 Hz, 1H, $C\boldsymbol{CH^7}H^8C$), 0.85 (d, $^2J_{H-H}$ = 4.6 Hz, 1H, $CCH^7\boldsymbol{H^8}C$), 0.71 (ddd, J_{H-H} = 9.1, 5.4, 3.9 Hz, 1H, $CCH^3H^4C\boldsymbol{H^5}H^6$), 0.51 (ddd, J_{H-H} = 9.2, 5.5, 4.1 Hz, 1H,$CCH^3\boldsymbol{H^4}CH^5H^6$), 0.49 (ddd, J_{H-H} = 9.1, 5.4, 3.9 Hz, 1H, $CCH^3H^4CH^5\boldsymbol{H^6}$).

^{13}C{^{1}H} NMR (101 MHz, 298 K, C_6D_6): δ 152.0 (Ar***C***), 138.6 (Ar***C***), 126.9 (Ar***C***H), 124.0 (Ar***C***H), 60.2 ($\boldsymbol{C}CH^7H^8C$), 59.4 ($(CH_3)(CH_3)\boldsymbol{C}CH^1H^2C(CH_3)(CH_3)$), 58.0 ($(CH_3)(CH_3)C\boldsymbol{C}H^1H^2C(CH_3)(CH_3)$), 41.4 ($(CH_3)(CH_3)CCH^1H^2\boldsymbol{C}(CH_3)(CH_3)$), 31.0 ($(\boldsymbol{C}H_3)(CH_3)CCH^1H^2C(CH_3)(CH_3)$), 30.2 ($(CH_3)(\boldsymbol{C}H_3)CCH^1H^2C(CH_3)(CH_3)$), 28.1 ($\boldsymbol{C}H(CH_3)(CH_3)$, 27.7 ($\boldsymbol{C}H(CH_3)(CH_3)$, 27.6 ($CH(\boldsymbol{C}H_3)(CH_3)$, 27.5 ($CH(\boldsymbol{C}H_3)(CH_3)$, 25.6 ($CH(\boldsymbol{C}H_3)(CH_3)$, 25.3 ($CH(\boldsymbol{C}H_3)(CH_3)$, 24.8 ($(CH_3)(CH_3)CCH^1H^2C(\boldsymbol{C}H_3)(CH_3)$), 24.5 ($(CH_3)(CH_3)CCH^1H^2C(CH_3)(\boldsymbol{C}H_3)$), 15.7 ($C\boldsymbol{C}H^7H^8C$) , 14.8 ($C\boldsymbol{C}H^3H^4CH^5H^6$), 6.8 ($CCH^3H^4\boldsymbol{C}H^5H^6$)), 5.8 ($CCH^7H^8\boldsymbol{C}$).

Anal. Calc. ($C_{24}H_{37}N$): C, 84.89; H, 10.98; N, 4.12. Found: C, 85.38; H, 10.77; N, 3.83.

Preparation of 5b

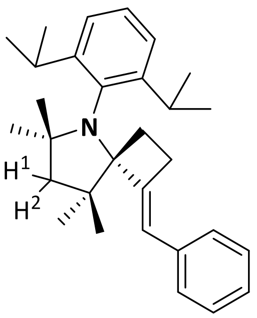

Following the general procedure A, the reaction was performed with **1b** (40.0 mg, 0.14 mmol, 1.0 equiv) and (cyclopropylidenemethyl)benzene (18.3 mg, 0.14 mmol, 1.0 equiv.) in C_6D_6 (1.0 mL) and reacted at 60 °C in a silicone oil bath for 2 hours. Quantitative ^{1}H NMR spectroscopy revealed that the starting material (**1b**) was completely consumed and formation of the product (**5b**) (NMR yield = 75 %). Isolated product (**5b**) was obtained as white powder (37.8 mg, 0.09 mmol, 65 %) after purification by column chromatography (SiO_2, DCM/hexane, v:v, 5:95).

^{1}H NMR (400 MHz, 298 K, C_6D_6): δ 7.27 – 7.22 (m, 2H, ArC***H***), 7.22 – 7.16 (m, 5H, ArC***H***), 7.06 – 6.99 (m, 1H, ArC***H***), 6.55 (t, $^4J_{H-H}$ = 2.8 Hz, 1H, C=C***H***Ph), 4.04 (hept, $^3J_{H-H}$ = 6.7 Hz, 1H, C***H***(CH$_3$)(CH$_3$)), 3.71 (hept, $^3J_{H-H}$ = 6.8 Hz, 1H, C***H***(CH$_3$)(CH$_3$)), 2.42 – 2.20 (m, 2H, CC***H$_2$***CH$_2$C=CHPh), 2.10 (d, $^2J_{H-H}$ = 12.9 Hz, 1H, (CH$_3$)$_2$C***H^1***H^{2}C(CH$_3$)$_2$), 2.03 – 1.95 (m, 2H, CCH$_2$C***H$_2$***C=CHPh), 1.77 (d, $^2J_{H-H}$ = 12.8 Hz, 1H, (CH$_3$)$_2$CCH^1***H^2***C(CH$_3$)$_2$), 1.35 (s, 3H, (C***H$_3$***)(CH$_3$)CCH^{1}H^{2}C(CH$_3$)(CH$_3$)), 1.34 (s, 3H, (CH$_3$)(C***H$_3$***)CCH^{1}H^{2}C(CH$_3$)(CH$_3$)), 1.28 (d, $^3J_{H-H}$ = 6.9 Hz, 3H, CH(C***H$_3$***)(CH$_3$)), 1.27 (d, $^3J_{H-H}$ = 6.9 Hz, 3H, CH(CH$_3$)(C***H$_3$***)), 1.17 (d, $^3J_{H-H}$ = 6.8 Hz, 3H, CH(C***H$_3$***)(CH$_3$)), 1.13 (s, 3H, (CH$_3$)(CH$_3$)CH^{1}H^{2}C(C***H$_3$***)(CH$_3$)), 1.10 (s, 3H, (CH$_3$)(CH$_3$)CH^{1}H^{2}C(CH$_3$)(C***H$_3$***)), 0.89 (d, $^3J_{H-H}$ = 6.6 Hz, 3H, CH(CH$_3$)(C***H$_3$***)).

^{13}C{^{1}H} NMR (101 MHz, 298 K, C_6D_6): δ 154.7 (Ar***C***), 153.8 (CCH$_2$CH$_2$***C***=CHPh), 138.6 (Ar***C***), 136.9 (Ar***C***), 128.7 (Ar***C***H), 128.5 (Ar***C***H), 126.3 (Ar***C***H), 125.2 (Ar***C***H), 124.8 (Ar***C***H), 124.3 (CCH$_2$CH$_2$C=***C***HPh), 81.7 (***C***CH$_2$CH$_2$C=CHPh), 61.5 ((CH$_3$)$_2$***C***CH^{1}H^{2}C(CH$_3$)$_2$), 54.3 ((CH$_3$)$_2$C***C***H^{1}H^{2}C(CH$_3$)$_2$), 42.7 ((CH$_3$)$_2$CCH^{1}H^2***C***(CH$_3$)$_2$), 34.0 ((***C***H$_3$)(CH$_3$)CCH^{1}H^{2}C(CH$_3$)(CH$_3$)), 29.6 (***C***H(CH$_3$)(CH$_3$)), 29.1 (***C***H(CH$_3$)(CH$_3$)), 28.3 (C***C***H$_2$CH$_2$C=CHPh), 27.8 (CH(***C***H$_3$)(CH$_3$)), 27.6 (CH(***C***H$_3$)(CH$_3$)), 27.0 (CH(***C***H$_3$)(CH$_3$)), 27.0 (CH(***C***H$_3$)(CH$_3$)), 26.2 ((***C***H$_3$)(CH$_3$)CCH^{1}H^{2}C(CH$_3$)(CH$_3$)), 25.4 (CCH$_2$***C***H$_2$C=CHPh), 25.0 ((CH$_3$)(CH$_3$)CCH^{1}H^{2}C(CH$_3$)(***C***H$_3$)), 24.3 ((CH$_3$)(CH$_3$)CCH^{1}H^{2}C(***C***H$_3$)(CH$_3$)).

HRMS (APCL) m/z: [M - H]$^+$ Calcd for [$C_{30}H_{41}N$ - H]$^+$: 414.3155; Found: 414.3145.

Preparation of 5c

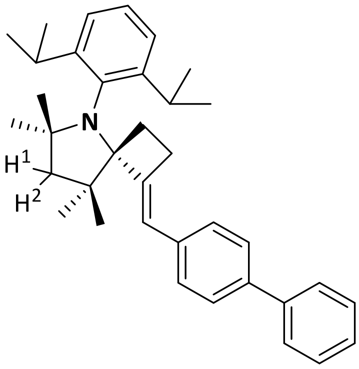

Following the general procedure A, the reaction was performed with **1b** (40.0 mg, 0.14 mmol, 1.0 equiv) and 4-(cyclopropylidenemethyl)-1,1'-biphenyl (28.9 mg, 0.14 mmol, 1.0 equiv.) in C_6D_6 (1.0 mL) and reacted at 60 °C in a silicone oil bath for 2 hours. Quantitative ^{1}H NMR spectroscopy revealed that product (**5c**) was completely formed (NMR yield = 80 %). Isolated product (**5c**) was obtained as white powder (48.2 mg, 0.10 mmol, 70 %) after purification by column chromatography (SiO_2, DCM/hexane, v:v, 5:95).

^{1}H NMR (400 MHz, 298 K, C_6D_6): δ 7.54 – 7.47 (m, 4H, ArC***H***), 7.32 – 7.27 (m, 2H, ArC***H***), 7.26 – 7.20 (m, 2H, ArC***H***), 7.19 (m, 3H, ArC***H***), 7.14 – 7.10 (m, 1H, ArC***H***), 6.60 (t, $^4J_{H\text{-}H}$ = 2.8 Hz, 1H, C=C***H***Ar), 4.07 (hept, $^3J_{H\text{-}H}$ = 6.7 Hz, 1H, C***H***(CH$_3$)(CH$_3$)), 3.73 (hept, $^3J_{H\text{-}H}$ = 6.8 Hz, 1H, C***H***(CH$_3$)(CH$_3$)), 2.43 – 2.26 (m, 2H, CC***H$_2$***CH$_2$C=CHAr), 2.14 (d, $^2J_{H\text{-}H}$ = 13.0 Hz, 1H, (CH$_3$)$_2$C***H^1***H^{2}C(CH$_3$)$_2$), 2.09 – 2.00 (m, 2H, CCH$_2$C***H$_2$***C=CHAr), 1.80 (d, $^2J_{H\text{-}H}$ = 13.0 Hz, 1H, (CH$_3$)$_2$CCH^1***H^2***C(CH$_3$)$_2$), 1.37 (s, 3H, (C***H$_3$***)(CH$_3$)CCH^{1}H^{2}C(CH$_3$)(CH$_3$)), 1.37 (s, 3H, (CH$_3$)(C***H$_3$***)CCH^{1}H^{2}C(CH$_3$)(CH$_3$)), 1.31 (d, $^3J_{H\text{-}H}$ = 6.8 Hz, 3H, CH(C***H$_3$***)(CH$_3$)), 1.29 (d, $^3J_{H\text{-}H}$ = 6.7 Hz, 3H, CH(CH$_3$)(C***H$_3$***)), 1.20 (d, $^3J_{H\text{-}H}$ = 6.7 Hz, 3H, CH(C***H$_3$***)(CH$_3$)), 1.17 (s, 3H, (CH$_3$)(CH$_3$)CH^{1}H^{2}C(C***H$_3$***)(CH$_3$)), 1.12 (, 3H, (CH$_3$)(CH$_3$)CH^{1}H^{2}C(CH$_3$)(C***H$_3$***)), 0.94 (d, $^3J_{H\text{-}H}$ = 6.6 Hz, 3H, CH(CH$_3$)(C***H$_3$***)).

^{13}C{^{1}H} NMR (101 MHz, 298 K, C_6D_6): δ 154.8 (Ar***C***), 153.5 (CCH$_2$CH$_2$***C***=CHAr), 141.0 (Ar***C***), 138.9 (Ar***C***), 137.4 (Ar***C***), 136.6 (Ar***C***), 128.7 (Ar***C***H), 127.3 (Ar***C***H), 127.2 (Ar***C***H), 127.1 (Ar***C***H), 126.9 (Ar***C***H), 124.9 (Ar***C***H), 124.5 (Ar***C***H), 123.6 (CCH$_2$CH$_2$C=***C***HAr), 81.5 (***C***CH$_2$CH$_2$C=CHAr), 61.3 ((CH$_3$)(CH$_3$)***C***CH^{1}H^{2}C(CH$_3$)(CH$_3$)), 54.0 ((CH$_3$)(CH$_3$)C***C***H^{1}H^{2}C(CH$_3$)(CH$_3$)), 42.4 ((CH$_3$)(CH$_3$)CCH^{1}H^2***C***(CH$_3$)(CH$_3$)), 33.7 ((***C***H$_3$)(CH$_3$)CCH^{1}H^{2}C(CH$_3$)(CH$_3$)), 29.4 (***C***H(CH$_3$)(CH$_3$)), 28.8 (***C***H(CH$_3$)(CH$_3$)), 28.1 (C***C***H$_2$CH$_2$C=CHPh), 27.5 (CH(***C***H$_3$)(CH$_3$)), 27.3 (CH(CH$_3$)(***C***H$_3$)), 26.7 (CH(***C***H$_3$)(CH$_3$)), 26.7 (CH(CH$_3$)(***C***H$_3$)), 26.0 ((***C***H$_3$)(CH$_3$)CCH^{1}H^{2}C(CH$_3$)(CH$_3$)), 25.2 (CCH$_2$***C***H$_2$C=CHPh), 24.7 ((CH$_3$)(CH$_3$)CCH^{1}H^{2}C(CH$_3$)(***C***H$_3$)), 24.0 ((CH$_3$)(CH$_3$)CCH^{1}H^{2}C(***C***H$_3$)(CH$_3$)).

HRMS (APCL) m/z: [M - H]$^+$ Calcd for [$C_{36}H_{45}N$ - H]$^+$: 490.3468; Found: 490.3464.

Preparation of 5d

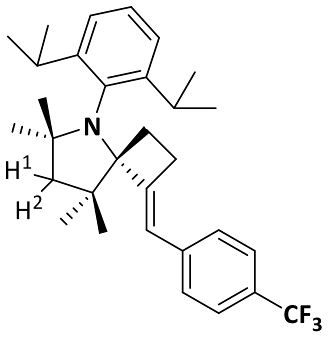

Following the general procedure A, the reaction was performed with **1b** (40.0 mg, 0.14 mmol, 1.0 equiv) and 1-(cyclopropylidenemethyl)-4-(trifluoromethyl)benzene (27.7 mg, 0.14 mmol, 1.0 equiv.) in C_6D_6 (1.0 mL) and reacted at 60 °C in a silicone oil bath for 1 hours. Quantitative ^{1}H NMR spectroscopy revealed that product (**5d**) was completely formed (NMR yield = 70 %). Isolated product (**5d**) was obtained as white powder (44.0 mg, 0.09 mmol, 65 %) after purification by column chromatography (SiO_2, DCM/hexane, v:v, 5:95).

^{1}H NMR (400 MHz, 298 K, C_6D_6): δ 7.36 (d, $^3J_{H-H}$ = 8.1 Hz, 2H, ArC***H***), 7.23 – 7.16 (m, 3H, ArC***H***), 7.02 (d, $^3J_{H-H}$ = 8.1 Hz, 2H, ArC***H***), 6.43 (t, $^4J_{H-H}$ = 2.8 Hz, 1H, C=C***H***PhCF_3), 3.90 (hept, $^3J_{H-H}$ = 6.7 Hz, 1H, C***H***(CH_3)(CH_3)), 3.66 (hept, $^3J_{H-H}$ = 6.7 Hz, 1H, C***H***(CH_3)(CH_3)), 2.26 – 2.10 (m, 2H, C***H₂***CH_2C=CHPhCF_3), 2.04 (d, $^2J_{H-H}$ = 13.0 Hz, 1H, $(CH_3)_2$C$\boldsymbol{H^1}$$H^2C(CH_3)_2$), 2.00 – 1.94 (m, 2H, CCH_2C***H₂***C=CHPhCF_3), 1.77 (d, $^2J_{H-H}$ = 12.8 Hz, 1H, $(CH_3)_2$CC$H^1$$\boldsymbol{H^2}C(CH_3)_2$), 1.34 (s, 3H, (C***H₃***)(CH_3)CCH^1H^2C(CH_3)(CH_3)), 1.31 (s, 3H, (CH_3)(C***H₃***)CCH^1H^2C(CH_3)(CH_3)), 1.27 (d, $^3J_{H-H}$ = 6.5 Hz, 3H, CH(C***H₃***)(CH_3)), 1.25 (d, $^3J_{H-H}$ = 6.5 Hz, 3H, CH(C***H₃***)(CH_3)), 1.18 (d, $^3J_{H-H}$ = 6.7 Hz, 3H, CH(C***H₃***)(CH_3)), 1.09 (s, 3H, (CH_3)(CH_3)CH^1H^2C(C***H₃***)(CH_3)), 1.07 (s, 3H, (CH_3)(CH_3)CH^1H^2C(CH_3)(C***H₃***)), 0.79 (d, $^3J_{H-H}$ = 6.6 Hz, 3H, CH(CH_3)(C***H₃***)).

^{13}C{^{1}H} NMR (101 MHz, 298 K, C_6D_6): δ 158.7 (Ar***C***), 153.7 (CCH_2CH_2***C***=CHPhCF_3), 141.8 (Ar***C***), 136.7(Ar***C***), 127.2 (Ar***C***H), 125.7 (q, $^3J_{C-F}$ = 3.7 Hz, Ar***C***CF_3), 125.1 (q, $^1J_{C-F}$ = 270.4 Hz, ArC***C***F_3), 125.2 (Ar***C***H), 124.9 (Ar***C***H), 123.0 (Ar***C***H), 81.7 (***C***CH_2CH_2C=CHPhCF_3), 61.6 (CH_3)(CH_3)***C***C$H^1$$\boldsymbol{H^2}$C($CH_3$)($CH_3$), 54.3 (($CH_3$)($CH_3$)C***C***$H^1$$\boldsymbol{H^2}$C($CH_3$)($CH_3$)), 42.7 (($CH_3$)($CH_3$)CC$H^1$$\boldsymbol{H^2}$***C***($CH_3$)($CH_3$)), 34.0 ((***C***$H_3$)($CH_3$)CC$H^1$$\boldsymbol{H^2}$C($CH_3$)($CH_3$)), 29.6 (***C***H($CH_3$)($CH_3$)), 29.0 (***C***H($CH_3$)($CH_3$)), 28.2 (C***C***$H_2CH_2$C=CHPh$CF_3$), 27.7 (CH(***C***$H_3$)($CH_3$)), 27.5 (CH(***C***$H_3$)($CH_3$)), 27.0 (CH(***C***$H_3$)($CH_3$)), 27.0 (CH(***C***$H_3$)($CH_3$)), 26.2 ((***C***$H_3$)($CH_3$)CC$H^1$$\boldsymbol{H^2}$C($CH_3$)($CH_3$)), 25.4 ($CCH_2$***C***$H_2$C=CHPh$CF_3$), 25.0 (($CH_3$)($CH_3$)CC$H^1$$\boldsymbol{H^2}$C($CH_3$)(***C***$H_3$)), 24.3 (($CH_3$)($CH_3$)CC$H^1$$\boldsymbol{H^2}$C(***C***$H_3$)($CH_3$)).

^{19}F-NMR (376.5 MHz, 298 K, C_6D_6) δ -61.94 (s, 3F, C***F₃***).

HRMS (APCL) m/z: $[M - H]^+$ Calcd for $[C_{31}H_{40}F_3N - H]^+$: 482.3029; Found: 482.3025.

Preparation of 5e

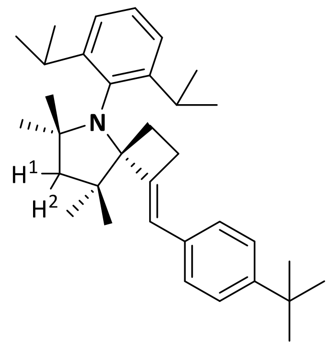

Following the general procedure A, the reaction was performed with **1b** (40.0 mg, 0.14 mmol, 1.0 equiv) and 1-(tert-butyl)-4-(cyclopropylidenemethyl)benzene (26.1 mg, 0.14 mmol, 1.0 equiv.) in C_6D_6 (1.0 mL) and reacted at 60 °C in a silicone oil bath for 4 hours. Quantitative ^{1}H NMR spectroscopy revealed that product (**5e**) was completely formed (NMR yield = 70 %). Isolated product (**5e**) was obtained as white powder (42.9 mg, 0.09 mmol, 65 %) after purification by column chromatography (SiO_2, DCM/hexane, v:v, 10:90).

^{1}H NMR (400 MHz, 298 K, C_6D_6): δ 7.31 – 7.26 (m, 4H, ArC***H***), 7.20 – 7.16 (m, 3H, ArC***H***), 6.60 (t, $^4J_{H-H}$ = 2.8 Hz, 1H, C=C***H***Ph^{t}Bu), 4.09 (hept, $^3J_{H-H}$ = 6.8 Hz, 1H, C***H***(CH_3)(CH_3)), 3.74 (hept, $^3J_{H-H}$ = 6.7 Hz, 1H, C***H***(CH_3)(CH_3)), 2.53 – 2.27 (m, 2H, CC***H₂***CH_2C=CHPh(CH_3)$_3$), 2.13 (d, $^2J_{H-H}$ = 12.8 Hz, 1H, (CH_3)$_2$C***H¹***H²C(CH_3)$_2$), 2.08 – 1.98 (m, 2H, CCH_2C***H₂***C=CHPh(CH_3)$_3$), 1.80 (d, $^2J_{H-H}$ = 12.8 Hz, 1H, (CH_3)$_2$CCH¹***H²***C(CH_3)$_2$), 1.37 (s, 3H, (C***H₃***)(CH_3)CCH¹H²C(CH_3)(CH_3)), 1.35 (s, 3H, (CH_3)(C***H₃***)CCH¹H²C(CH_3)(CH_3)), 1.29 (d, $^3J_{H-H}$ = 6.9 Hz, 3H, CH(C***H₃***)(CH_3)), 1.28 (d, $^3J_{H-H}$ = 6.9 Hz, 3H, CH(CH_3)(C***H₃***)), 1.23 (s, 9H, PhC(CH_3)$_3$), 1.19 (d, $^3J_{H-H}$ = 6.7 Hz, 3H, CH(C***H₃***)(CH_3)), 1.17 (s, 3H, (CH_3)(CH_3)CH¹H²C(C***H₃***)(CH_3)), 1.11 (s, 3H, (CH_3)(CH_3)CH¹H²C(CH_3)(C***H₃***)), 0.93 (d, $^3J_{H-H}$ = 6.6 Hz, 3H, CH(CH_3)(C***H₃***)).

^{13}C{^{1}H} NMR (101 MHz, 298 K, C_6D_6): δ 154.1 (Ar***C***), 153.7 (CCH_2CH_2***C***=CHPh(CH_3)$_3$), 148.9 (Ar***C***), 137.0 (Ar***C***), 136.0 (Ar***C***), 125.7 (Ar***C***H), 125.2 (Ar***C***H), 124.8 (Ar***C***H), 124.2 (CCH_2CH_2C=***C***HPh(CH_3)$_3$), 81.7 (***C***CH_2CH_2C=CHPh(CH_3)$_3$), 61.5 (CH_3)(CH_3)***C***CH¹H²C(CH_3)(CH_3), 54.3 (CH_3)(CH_3)C***C***H¹H²C(CH_3)(CH_3), 42.8 ((CH_3)(CH_3)CCH¹H²***C***(CH_3)(CH_3)), 34.5 ((***C***H₃)(CH_3)CCH¹H²C(CH_3)(CH_3)), 34.0 (***C***(CH_3)$_3$), 31.4 (C(***C***H₃)$_3$), 29.7 (***C***H(CH_3)(CH_3)), 29.1 (***C***H(CH_3)(CH_3)), 28.5 (C***C***H₂CH_2C=CHPh(CH_3)$_3$), 27.8 (CH(***C***H₃)(CH_3)), 27.6 (CH(***C***H₃)(CH_3)), 27.0 (CH(***C***H₃)(CH_3)), 27.0 (CH(***C***H₃)(CH_3)), 26.3 (***C***H₃)(CH_3)CCH¹H²C(CH_3)(CH_3), 25.5 (CCH_2***C***H₂C=CHPh(CH_3)$_3$), 25.0 (CH_3)(CH_3)CCH¹H²C(CH_3)(***C***H₃), 24.3 (CH_3)(CH_3)CCH¹H²C(***C***H₃)(CH_3).

HRMS (APCL) m/z: [M - H]$^+$ Calcd for [$C_{34}H_{49}N$ - H]$^+$: 470.3781; Found: 470.3773.

Preparation of 5f

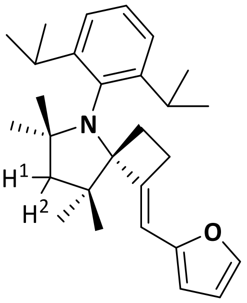

Following the general procedure A, the reaction was performed with **1b** (40.0 mg, 0.14 mmol, 1.0 equiv) and 2-(cyclopropylidenemethyl)furan (16.8 mg, 0.14 mmol, 1.0 equiv.) in C_6D_6 (1.0 mL) and reacted at 60 °C in a silicone oil bath for 3 hours. Quantitative ^{1}H NMR spectroscopy revealed that product (**5f**) was completely formed (NMR yield = 50 %). Isolated product (**5f**) was obtained as white powder (22.7 mg, 0.06 mmol, 40 %) after purification by column chromatography (SiO_2, DCM/hexane, v:v, 5:95).

^{1}H NMR (400 MHz, 298 K, C_6D_6): δ 7.19 – 7.16 (m, 3H, ArC***H***), 7.09 – 7.07 (m, 1H, ArC***H***), 6.53 (t, $^3J_{H-H}$ = 2.8 Hz, 1H, C=C***H***Ar), 6.15 (dd, J_{H-H} = 3.3, 1.9 Hz, 1H, ArC***H***), 5.98 (d, J_{H-H} = 3.3 Hz, 1H, ArC***H***), 3.96 (hept, $^3J_{H-H}$ = 6.7 Hz, 1H, C***H***(CH_3)(CH_3)), 3.68 (hept, $^3J_{H-H}$ = 6.8 Hz, 1H, C***H***(CH_3)(CH_3)), 2.41 – 2.21 (m, 2H, CC***H₂***CH_2C=CHAr), 2.00 – 1.97 (m, 2H, CCH_2C***H₂***C=CHAr), 1.97 (d, $^2J_{H-H}$ = 12.9 Hz, 1H, $(CH_3)_2$C***H¹***H²C(CH_3 ₂)), 1.71 (d, $^2J_{H-H}$ = 12.8 Hz, 1H, $(CH_3)_2$CCH¹***H²***C$(CH_3)_2$), 1.31 (s, 3H, (C***H₃***)(CH_3)CCH¹H²C(CH_3)(CH_3)), 1.28 (d, $^3J_{H-H}$ = 6.9 Hz, 1H), 1.27 (d, $^3J_{H-H}$ = 6.9 Hz, 3H, CH(C***H₃***)(CH_3)), 1.25 (d, $^3J_{H-H}$ = 6.9 Hz, 3H, CH(C***H₃***)(CH_3)), 1.25 (s, 3H, (CH_3)(C***H₃***)CCH¹H²C(CH_3)(CH_3)), 1.17 (d, $^3J_{H-H}$ = 6.8 Hz, 3H, CH(C***H₃***)(CH_3)), 1.08 (s, 3H, (CH_3)(CH_3)CH¹H²C(C***H₃***)(CH_3)), 1.07 (s, 3H, (CH_3)(CH_3)CH¹H²C(CH_3)(C***H₃***)), 0.96 (d, $^3J_{H-H}$ = 6.6 Hz, 3H, CH(CH_3)(C***H₃***)).

^{13}C{^{1}H} NMR (101 MHz, 298 K, C_6D_6): δ 154.3 (Ar***C***), 153.9 (Ar***C***), 153.8 (CCH_2CH_2***C***=CHAr), 141.2 (Ar***C***), 136.9 (Ar***C***H), 125.3 (Ar***C***H), 124.8 (Ar***C***H), 113.6 (CCH_2CH_2C=***C***HAr), 111.5 (Ar***C***H), 106.6 (Ar***C***H), 81.2 (***C***CH_2CH_2C=CHAr), 61.5 (CH_3)(CH_3)***C***CH¹H²C(CH_3)(CH_3), 54.3 (CH_3)(CH_3)C***C***H¹H***²***C(CH_3)(CH_3), 42.5 (CH_3)(CH_3)CCH¹H***²******C***(CH_3)(CH_3), 33.9 ((***C***H₃)(CH_3)CCH¹H***²***C(CH_3)(CH_3)), 29.7 (***C***H(CH_3)(CH_3)), 29.0 (***C***H(CH_3)(CH_3)), 28.1 (C***C***H₂CH_2C=CHPh), 27.6 (CH(***C***H₃)(CH_3)), 27.2 (CH(CH_3)(***C***H₃)), 27.0 (CH(***C***H₃)(CH_3)), 26.9 (CH(CH_3)(***C***H₃)), 25.9 (***C***H₃)(CH_3)CCH¹H***²***C(CH_3)(CH_3), 25.4 (CCH_2***C***H₂C=CHPh), 25.0 (CH_3)(CH_3)CCH¹H***²***C(CH_3)(***C***H₃), 24.4 (CH_3)(CH_3)CCH¹H***²***C(***C***H₃)(CH_3).

HRMS (APCL) m/z: [M - H]$^+$ Calcd for [$C_{28}H_{39}NO$ - H]$^+$: 404.2948; Found: 404.2945.

Preparation of S1

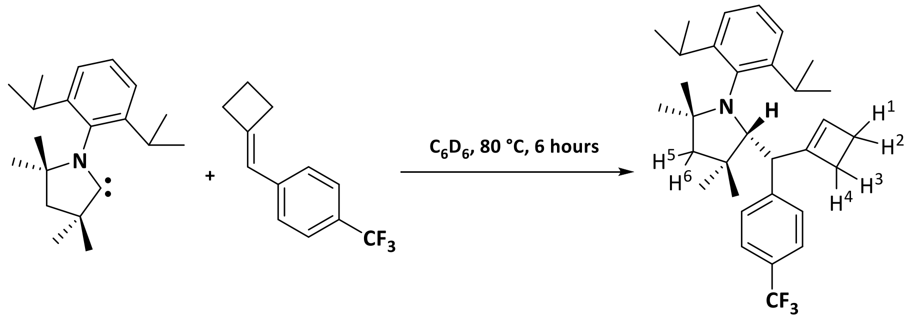

S1

In a N_2 filled glovebox, **1b** (40.0 mg, 0.14 mmol, 1.0 equiv) and 1-(cyclobutylidenemethyl)-4-(trifluoromethyl)benzene (29.7 mg, 0.14 mmol, about 1.0 equiv.) were dissolved in C_6D_6 (1.0 mL) and transferred to a J-Young NMR tube. Ferrocene in a sealed glass capillary containing C_6D_6 (δ_H = 4.0 ppm) was added as internal standard. The tube was sealed, removed from the glovebox, and an initial ^{1}H NMR spectra recorded. The tube was heated to 80 °C in a silicone oil bath for 6 hours. Quantitative ^{1}H NMR spectroscopy revealed that product (**S1**) and (**S2** – See Figure S9 for structure) were formed (NMR yield = **S1**: 40% + **S2**: 10 %). The reaction mixture was diluted with *n*-hexane (1 mL) and the solution decanted into a scintillation vial. The isolated product (**S1**) was obtained as colourless crystals (20.9 mg, 0.04 mmol, 30 %) after purification by column chromatography (SiO_2, DCM/hexane, v:v, 5:95). A small amount of the minor product (**S2**) was successfully crystalized from the remaining concentrated supernatant (DCM/hexane, v:v, 10:90, 1 mL) at –20 °C and subjected to crystallographic analysis. However, NMR characterization was not feasible due to the difficulty in collecting sufficient material.

NMR data of ***S1***:

^{1}H NMR (400 MHz, 298 K, C_6D_6): δ 7.23 (d, $^3J_{H-H}$ = 8.6 Hz, 2H, ArC***H***), 7.14 – 7.09 (m, 2H, ArC***H***), 7.07 – 7.04 (m, 1H, ArC***H***), 7.03 (d, $^3J_{H-H}$ = 8.6 Hz, 2H, ArC***H***), 5.00 (d, $^4J_{H-H}$ = 0.8 Hz, 1H, CHCHArC=C***H***CH^{1}H^{2}CH^{3}H^4), 4.27 (d, $^3J_{H-H}$ = 10.3 Hz, 1H, C***H***CHArC=CHCH$_2$CH$_2$), 3.87 (hept, $^3J_{H-H}$ = 6.7 Hz, 1H, C***H***(CH$_3$)(CH$_3$)), 3.54 (d, $^3J_{H-H}$ = 10.2 Hz, 1H, CHC***H***ArC=CHCH$_2$CH$_2$), 3.25 (hept, $^3J_{H-H}$ = 6.7 Hz, 1H, C***H***(CH$_3$)(CH$_3$)), 2.11 (d, $^2J_{H-H}$ = 12.6 Hz, 1H, (CH$_3$)(CH$_3$)CC***H^5***H^{6}C(CH$_3$)(CH$_3$)), 1.96 (m, 1H, CHCHArC=CHC***H^1***H^{2}CH^{3}H^4), 1.80 (m, 2H, CHCHArC=CHCH^{1}H^{2}C***H^{3}H^4***), 1.61 (d, $^2J_{H-H}$ = 12.6 Hz, 1H, (CH$_3$)(CH$_3$)CCH^5***H^6***C(CH$_3$)(CH$_3$)), 1.54 (s, 3H, (C***H$_3$***)(CH$_3$)CCH^{5}H^{6}C(CH$_3$)(CH$_3$)), 1.50 (d, $^3J_{H-H}$ = 6.7 Hz, 3H, CH(CH$_3$)(C***H$_3$***)), 1.31 (d, $^3J_{H-H}$ = 6.8 Hz, 3H, CH(CH$_3$)(C***H$_3$***)), 1.26 (d, $^3J_{H-H}$ = 6.7 Hz, 3H, CH(CH$_3$)(C***H$_3$***)), 1.25 (d, $^3J_{H-H}$ = 6.7 Hz, 3H, CH(CH$_3$)(C***H$_3$***)), 1.19 (m, 1H, CHCHArC=CHCH^1***H^2***CH^{3}H^4), 1.07 (s, 3H, (CH$_3$)(C***H$_3$***)CCH^{5}H^{6}C(CH$_3$)(CH$_3$)), 0.88 (s, 3H, (CH$_3$)(CH$_3$)CCH^{5}H^{6}C(CH$_3$)(C***H$_3$***)), 0.83 (s, 3H, (CH$_3$)(CH$_3$)CCH^{5}H^{6}C(C***H$_3$***)(CH$_3$)).

^{19}F-NMR (376.5 MHz, 298 K, C_6D_6) δ -61.90 (s, 3F, C***F$_3$***).

^{13}C{^{1}H} NMR (101 MHz, 298 K, C_6D_6): δ 150.8 (CHCHAr***C***=CHCH$_2$CH$_2$), 149.6 (Ar***C***), 147.6 (Ar***C***),

147.3 (Ar***C***), 144.9 (Ar***C***), 129.2 (Ar***C***H), 126.3 (Ar***C***H), 124.8 (Ar***C***H), 124.7 (q, $^{3}J_{C\text{-}F}$ = 3.8 Hz, Ar***C***CF_3), 124.6 (q, $^{1}J_{C\text{-}F}$ = 270.4 Hz, ArC***C***F_3), 123.8 (CHCHArC=***C***HCH_2CH_2), 75.1 (***C***HCHArC=$CHCH^1H^2CH^3H^4$), 61.7 ((CH_3)(CH_3)***C***CH^5H^6C(CH_3)(CH_3)), 61.5 ((CH_3)(CH_3)C***C***H^5H^6C(CH_3)(CH_3)), 55.9 (CH***C***HArC=$CHCH^1H^2CH^3H^4$), 41.2 (CHCHArC=CH***C***$H^1H^2CH^3H^4$), 33.1 (CHCHArC=$CHCH^1H^2$***C***H^3H^4), 32.8 (***C***H(CH_3)(CH_3), 29.1 (***C***H(CH_3)(CH_3), 28.5 (CH(***C***H_3)(CH_3), 28.3 (CH(***C***H_3)(CH_3), 27.5 (CH(***C***H_3)(CH_3), 27.5 (CH(***C***H_3)(CH_3), 27.1 ((CH_3)(***C***H_3)CCH^5H^6C(CH_3)(CH_3)), 26.7 ((***C***H_3)(CH_3)CCH^5H^6C(CH_3)(CH_3)), 25.0 ((CH_3)(CH_3)CCH^5H^6***C***(CH_3)(CH_3)), 24.8 ((CH_3)(CH_3)CCH^5H^6C(CH_3)(***C***H_3)), 23.2 ((CH_3)(CH_3)CCH^5H^6C(***C***H_3)(CH_3)).

HRMS (APCL) m/z: $[M + H]^+$ Calcd for $[C_{32}H_{42}NF_3 + H]^+$: 498.3342; Found: 498.3334.

Preparation of S3

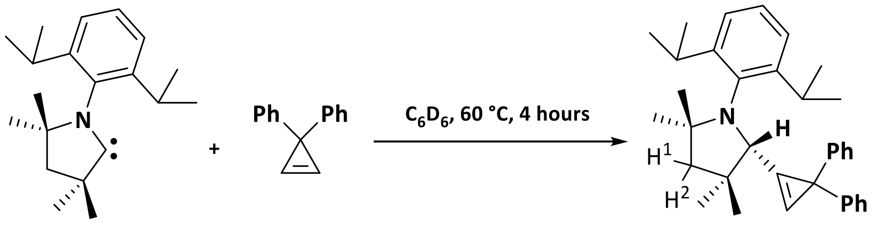

In a N_2 filled glovebox, **1b** (40.0 mg, 0.14 mmol, 1.0 equiv) and cycloprop-2-ene-1,1-diyldibenzene (26.9 mg, 0.14 mmol, about 1.0 equiv.) were dissolved in C_6D_6 (1.0 mL) and transferred to a J-Young NMR tube. Ferrocene in a sealed glass capillary containing C_6D_6 (δ_H = 4.0 ppm) was added as internal standard, and the reaction mixture was analysed by quantitative ^{1}H-NMR spectroscopy. The tube was sealed, removed from the glovebox, and an initial ^{1}H NMR spectra recorded. The reaction mixture was heated to 60 °C in a silicone oil bath for 4 hours. Quantitative ^{1}H-NMR revealed that product (**S3**) was formed (NMR yield = 65 %). The tube was returned to the glovebox, diluted with *n*-hexane (1 mL) and the reaction mixture was decanted into a scintillation vial. The volatiles removed *in vacuo* to yield a colourless oil and then recrystallized in hexane (0.5 ml) at -35 °C over 4 days. Colourless crystals (**S3**) were successfully obtained. The filtrated crystals were washed with cold *n*-pentane (3 x 0.5mL) and then dried in vacuo. Yield: 40.0 mg, 0.08 mmol, 60%.

^{1}H NMR (400 MHz, 298 K, C_6D_6): δ 7.29 – 7.24 (m, 2H, ArC***H***), 7.23 – 7.18 (m, 2H, ArC***H***), 7.18 – 7.15 (m, 2H, ArC***H***), 7.10 – 7.06 (m, 2H, ArC***H***), 7.05 – 6.98 (m, 5H, ArC***H***), 6.39 (d, $^4J_{H-H}$ = 1.5 Hz, 1H, CHC=C***H***CPh$_2$), 4.94 (d, $^4J_{H-H}$ = 1.7 Hz, 1H, C***H***C=CHCPh$_2$), 4.09 (hept, $^3J_{H-H}$ = 6.8 Hz, 1H, C***H***(CH$_3$)(CH$_3$)), 3.23 (hept, $^3J_{H-H}$ = 6.8 Hz, 1H, C***H***(CH$_3$)(CH$_3$)), 1.79 (d, $^3J_{H-H}$ = 12.7 Hz, 1H, (CH$_3$)$_2$ C***H^1***H^{2}C(CH$_3$)$_2$), 1.62 (d, $^3J_{H-H}$ = 12.7 Hz, 1H, (CH$_3$)$_2$CH^1***H^2***C(CH$_3$)$_2$), 1.31 (d, $^3J_{H-H}$ = 6.8 Hz, 3H, CH(CH$_3$)(C***H$_3$***)), 1.28 (d, $^3J_{H-H}$ = 6.7 Hz, 3H, CH(CH$_3$)(C***H$_3$***)), 1.27 (s, 3H, (C***H$_3$***)(CH$_3$)CCH^{1}H^{2}C(CH$_3$)(CH$_3$)), 1.20 (d, $^3J_{H-H}$ = 6.7 Hz, 3H, CH(CH$_3$)(C***H$_3$***)), 1.10 (s, 3H, (CH$_3$)(C***H$_3$***)CCH^{1}H^{2}C(CH$_3$)(CH$_3$)), 1.02 (s, 3H, (CH$_3$)(CH$_3$)CCH^{1}H^{2}C(C***H$_3$***)(CH$_3$)), 0.89 (d, $^3J_{H-H}$ = 6.7 Hz, 3H, CH(CH$_3$)(C***H$_3$***)), 0.84 (s, 3H, (CH$_3$)(CH$_3$)CCH^{1}H^{2}C(CH$_3$)(C***H$_3$***)).

^{13}C{^{1}H} NMR (101 MHz, 298 K, C_6D_6): δ 152.7 (Ar***C***), 151.3 (Ar***C***), 147.8 (CH***C***=CHCPh$_2$), 147.4 (Ar***C***), 138.8 (Ar***C***), 129.1 (Ar**C**H), 128.9 (Ar**C**H), 127.3 (Ar**C**H), 126.3 (Ar**C**H), 125.8 (Ar**C**H), 125.7 (Ar**C**H), 124.9 (Ar**C**H), 124.6 (Ar**C**H), 106.9 (CHC=***C***HCPh$_2$), 72.4 (***C***HC=CHCPh), 62.2 ((CH$_3$)(CH$_3$)***C***CH^{1}H^{2}C(CH$_3$)(CH$_3$)), 57.0 ((CH$_3$)(CH$_3$)C***C***H^{1}H^{2}C(CH$_3$)(CH$_3$)), 41.2 (CHC=CH***C***Ph$_2$), 35.6 ((***C***H$_3$)(CH$_3$)CCH^{1}H^{2}C(CH$_3$)(CH$_3$)), 31.2 ((CH$_3$)(***C***H$_3$)CCH^{1}H^{2}C(CH$_3$)(CH$_3$)), 29.5 (***C***H(CH$_3$)(CH$_3$), 28.8 (***C***H(CH$_3$)(CH$_3$), 28.8 (***C***H(CH$_3$)(CH$_3$), 27.6 (CH(***C***H$_3$)(CH$_3$), 26.6 (CH(***C***H$_3$)(CH$_3$), 26.2 (CH(***C***H$_3$)(CH$_3$), 25.4 ((CH$_3$)(CH$_3$)CCH^{1}H^2***C***(CH$_3$)(CH$_3$)), 25.3 ((CH$_3$)(CH$_3$)CCH^{1}H^{2}C(CH$_3$)(***C***H$_3$)), 25.3 ((CH$_3$)(CH$_3$)CCH^{1}H^{2}C(***C***H$_3$)(CH$_3$)), 24.7 ((CH$_3$)(CH$_3$)CCH^{1}H^2***C***(CH$_3$)(CH$_3$)).

Anal. Calc. ($C_{35}H_{43}N$): C, 88.00; H, 9.07; N, 2.93. Found: C, 88.07; H, 9.49; N, 3.05.

3) Additional Reactions

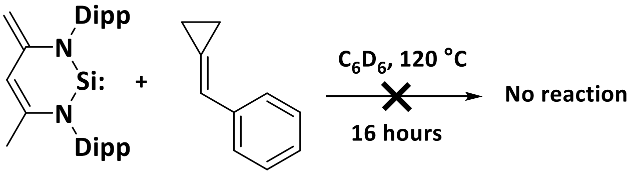

1b

In a N_2 filled glovebox, {DippNC(=CH_2)CH(Me)NDipp}Si (**1a**, 10.0 mg, 0.02 mmol, 1 eq) and (cyclopropylidenemethyl)benzene (2.9 mg, 0.02 mmol, 1.0 equiv.) were dissolved in C_6D_6 (0.6 mL) and transferred to a J-Young NMR tube. Ferrocene in a sealed glass capillary containing C_6D_6 (δ_H = 4.0 ppm) was added as internal standard. The tube was sealed, removed from the glovebox, and an initial ^1H NMR spectra recorded. The reaction mixture was heated to 120 °C in a silicone oil bath for 16 hours and no product formation was detected by ^1H NMR spectroscopy.

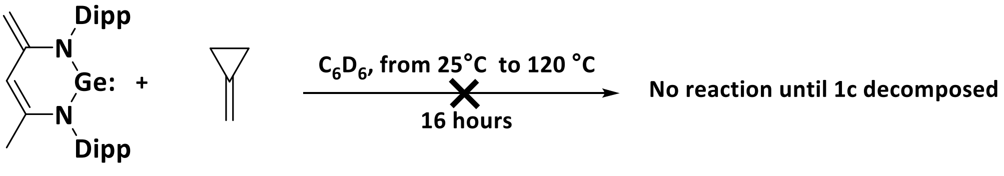

1c

In a N_2 filled glovebox, {DippNC(=CH_2)CH(Me)NDipp}Ge (**1c**, 10.0 mg, 0.02 mmol, 1 eq) and methylenecyclopropane (1.1 mg, 0.02 mmol, 1.0 equiv.) were dissolved in C_6D_6 (0.6 mL) and transferred to a J-Young NMR tube. Ferrocene in a sealed glass capillary containing C_6D_6 (δ_H = 4.0 ppm) was added as internal standard. The tube was sealed, removed from the glovebox, and an initial ^1H NMR spectra recorded. The reaction mixture was heated from 25 °C to 120 °C for in a silicone oil bath 16 hours and no product formation was detected by ^1H NMR spectroscopy.

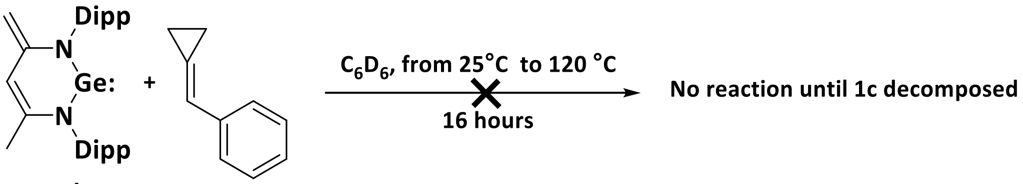

1c

In a N_2 filled glovebox, {DippNC(=CH_2)CH(Me)NDipp}Ge (**1c**, 10.0 mg, 0.02 mmol, 1 eq) and (cyclopropylidenemethyl)benzene (2.6 mg, 0.02 mmol, 1.0 equiv.) were dissolved in C_6D_6 (0.6 mL) and transferred to a J-Young NMR tube. Ferrocene in a sealed glass capillary containing C_6D_6 (δ_H = 4.0 ppm) was added as internal standard. The tube was sealed, removed from the glovebox, and an initial ^1H NMR spectra recorded. The reaction mixture was heated from 25 °C to 120 °C in a silicone oil bath for 16 hours and no product formation was detected by ^1H NMR spectroscopy.

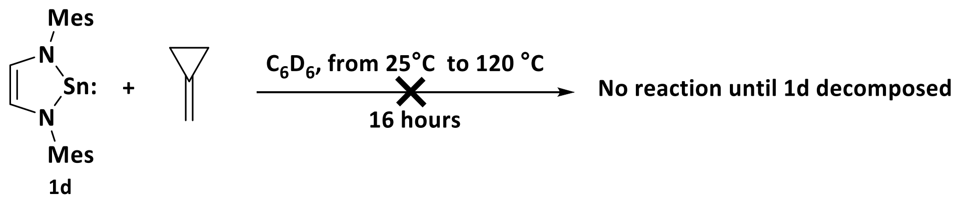

In a N_2 filled glovebox, sicarbine (**1d**, 10.0 mg, 0.02 mmol, 1 eq) and methylenecyclopropane (1.3 mg, 0.02 mmol, 1.0 equiv.) were dissolved in C_6D_6 (0.6 mL) and transferred to a J-Young NMR tube. Ferrocene in a sealed glass capillary containing C_6D_6 (δ_H = 4.0 ppm) was added as internal standard. The tube was sealed, removed from the glovebox, and an initial ^{1}H NMR spectra recorded. The reaction mixture was heated from 25 °C to 120 °C in a silicone oil bath for 16 hours and no product formation was detected by ^{1}H NMR spectroscopy.

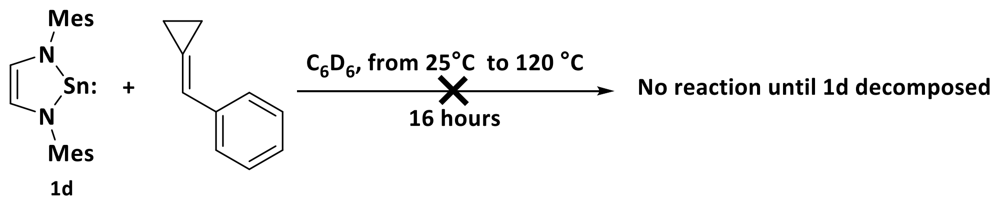

In a N_2 filled glovebox, sicarbine (**1d**, 10.0 mg, 0.02 mmol, 1 eq) and (cyclopropylidenemethyl)benzene (3.2 mg, 0.02 mmol, 1.0 equiv.) were dissolved in C_6D_6 (0.6 mL) and transferred to a J-Young NMR tube. Ferrocene in a sealed glass capillary containing C_6D_6 (δ_H = 4.0 ppm) was added as internal standard. The tube was sealed, removed from the glovebox, and an initial ^{1}H NMR spectra recorded. The reaction mixture was heated from 25 °C to 120 °C in a silicone oil bath for 16 hours and no product formation was detected by ^{1}H NMR spectroscopy.

4) Competition reactions

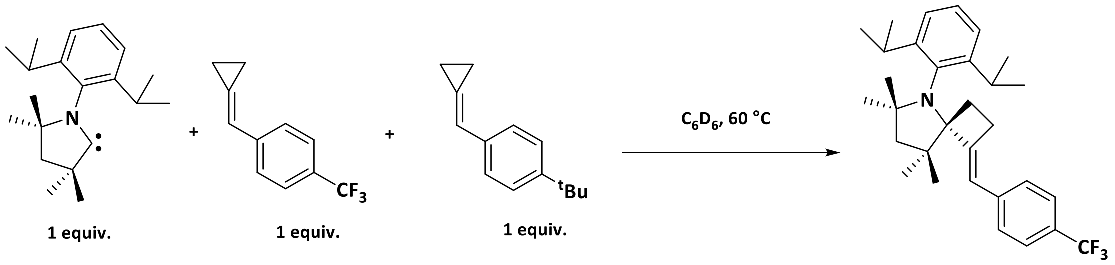

In a N_2 filled glovebox, **1b** (10.0 mg, 0.035 mmol, 1.0 equiv.), 1-(cyclopropylidenemethyl)-4-(trifluoromethyl)benzene (6.9 mg, 0.035 mmol, 1.0 equiv.) and 1-(tert-butyl)-4-(cyclopropylidenemethyl)benzene (6.5 mg, 0.035 mmol, about 1.0 equiv.) were dissolved in C_6D_6 (0.6 mL) and transferred to a J-Young NMR tube. Ferrocene in a sealed glass capillary containing C_6D_6 (δ_H = 4.0 ppm) was added as internal standard. The tube was sealed, removed from the glovebox, and an initial ^{1}H NMR spectra recorded. The reaction mixture was heated to 60 °C in a silicone oil bath for 2 hours. Quantitative ^{1}H NMR spectroscopy revealed that product (**5d**) was formed (NMR yield = 60 %) but no **5e** was detected.

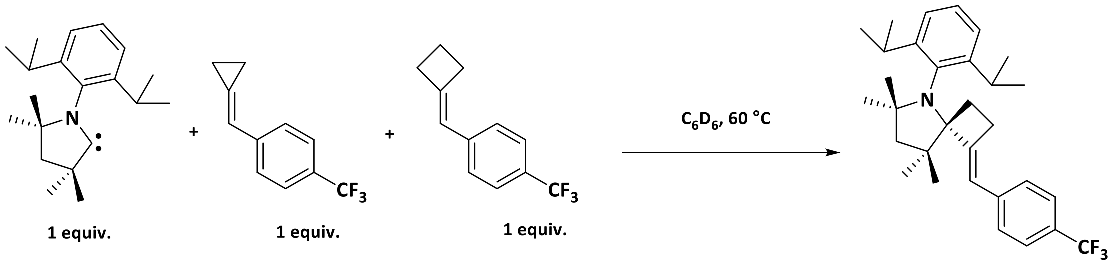

In a N_2 filled glovebox, **1b** (10.0 mg, 0.035 mmol, 1.0 equiv.), 1-(cyclopropylidenemethyl)-4-(trifluoromethyl)benzene (6.9 mg, 0.035 mmol, 1.0 equiv.) and 1-(cyclobutylidenemethyl)-4-(trifluoromethyl)benzene (7.4 mg, 0.035 mmol, about 1.0 equiv.) were dissolved in C_6D_6 (0.6 mL) and transferred to a J-Young NMR tube. Ferrocene in a sealed glass capillary containing C_6D_6 (δ_H = 4.0 ppm) was added as internal standard. The tube was sealed, removed from the glovebox, and an initial ^{1}H NMR spectra recorded. The reaction mixture was heated to 60 °C in a silicone oil bath for 2 hours. Quantitative ^{1}H NMR spectroscopy revealed that only the product (**5d**) was formed (NMR yield = 60 %).

5) Single Crystal X-ray Diffraction Data

	2	3	4
**CCDC number**	2522912	2497350	2486664
**formula**	$C_{33}H_{46}N_2Si$	$C_{33}H_{46}N_2Si$	$C_{24}H_{37}N$
**formula weight (g·mol^{-1})**	498.81	498.81	339.54
**colour, habit**	colourless block	clear colourless plate	colourless shard
**crystal size (mm)**	0.12 x 0.07 x 0.05	0.436 x 0.285 x 0.125	0.332 x 0.324 x 0.216
**crystal system**	monoclinic	triclinic	monoclinic
**space group**	$P2_1/n$ (no. 14)	*P*-1 (no. 2)	*I*2/a (no. 15)
a **(Å)**	8.79380(10)	11.8268(2)	15.76194(12)
b **(Å)**	11.12890(10)	16.0857(5)	8.80317(5)
c **(Å)**	30.9454(3)	16.0878(2)	30.9504(2)
α **(°)**	90	87.089(2)	90.0
β **(°)**	95.9530(10)	89.9550(10)	98.5171(7)
γ **(°)**	90	78.820(2)	90.0
**V (Å^3)**	3012.15(5)	2998.50(11)	4247.17(5)
**Z**	4	4 [b]	8
**T (K)**	173(2)	173(2)	174(4)
**D_c (g·cm^{-3})**	1.100	1.105	1.062
**radiation used, μ (mm^{-1})**	Cu Kα, 0.838	Cu Kα, 0.842	Cu Kα, 0.446
**F(000)**	1088	1088	1504
**absorption correction**	analytical	analytical	analytical
**min-max transmission**	0.770 - 1.000	0.802 - 0.912	0.890 - 0.924
**Θ range for data collection (°)**	2.871 - 68.347	3.810 - 73.684	5.228 - 73.611
**no. of unique reflns measured, obs [F > 4σ(F)]**	5498, 4871	18283, 14073	4259, 4033
**R_{int}, R_{sigma}**	0.0378, 0.0244	0.0598, 0.0405	0.0187, 0.0090
**completeness to Θ(full) (°)**	1.000 to 67.684	0.974 to 67.684	0.999 to 67.684
**no. of parameters, restraints**	334, 0	668, 0	256, 227
**R_1, wR_2 [F > 4σ(F)] [a]**	0.0366, 0.1002	0.0584, 0.1518	0.0419, 0.1086
**R_1, wR_2 [all data] [a]**	0.0411, 0.1030	0.0782, 0.1668	0.0436, 0.1100
**GooF**	1.056	1.045	1.025
**largest diff. Fourier peak, hole (eÅ^{-3})**	0.203, -0.249	0.36/ -0.34	0.251, −0.220

Table S1. Crystal Data, Data Collection and Refinement Parameters. Data were collected using Agilent Xcalibur 3 E Agilent (Mo radiation, compounds **3b**, **3c**, **3f**, and **S3**), Xcalibur PX Ultra A (Cu radiation, compounds **2a**, **5**, **S1**, and **S2**), and Rigaku XtaLAB Synergy-*i* (Cu radiation, compound **4**) diffractometers. Raw frame data was reduced using CrysAlisPro [S9], and the structures were solved and refined using the OLEX2,[S10] SHELXTL[S11] and SHELX-2018[S12] program systems. [a] $R_1 = \Sigma||F_o| - |F_c||/\Sigma|F_o|$; $wR_2 = \{\Sigma[w(F_o^2 - F_c^2)^2] / \Sigma[w(F_o^2)^2]\}^{1/2}$; $w^{-1} = \sigma_2(F_o^2) + (aP)^2 + bP$. [b] The asymmetric unit contains two crystallographically independent molecules.

data	5b	5c	5f
**CCDC number**	2486661	2486662	2486663
**formula**	$C_{30}H_{41}N$	$C_{36}H_{45}N$	$C_{28}H_{39}NO$
**formula weight (g·mol^{-1})**	415.64	491.73	405.60
**colour, habit**	colourless block	colourless block	clear colourless blocky
**crystal size (mm)**	0.802 x 0.511 x 0.385	0.554 x 0.389 x 0.155	0.521 x 0.298 x 0.181
**crystal system**	monoclinic	monoclinic	triclinic
**space group**	$P2_1/n$ (no. 14)	*C*2/c (no. 15)	*P*-1 (no. 2)
a **(Å)**	9.85131(12)	34.3981(4)	9.6843(4)
b **(Å)**	10.89568(13)	11.19474(12)	10.5771(4)
c **(Å)**	23.7670(3)	15.6646(2)	12.2670(4)
α **(°)**	90.0	90.0	77.947(3)
β **(°)**	96.0969(12)	99.0713(11)	76.510(3)
γ **(°)**	90.0	90.0	88.055(3)
**V (Å^3)**	2536.64(5)	5956.65(12)	1194.79(8)
**Z**	4	8	2
**T (K)**	172.95(10)	172.95(10)	173.05(10)
**D_c (g·cm^{-3})**	1.088	1.097	1.127
**radiation used, μ (mm^{-1})**	Mo Kα, 0.062	Mo Kα, 0.062	Mo Kα, 0.067
**F(000)**	912	2144	444
**absorption correction**	analytical	analytical	analytical
**min-max transmission**	0.966 - 0.983	0.976 - 0.992	0.973 - 0.991
**Θ range for data collection (°)**	2.543 - 28.393	2.377 - 28.320	2.445 - 28.328
**no. of unique reflns measured,obs [F > 4σ(F)]**	5949, 5180	7025, 5793	5158, 3938
**R_{int}, R_{sigma}**	0.0230, 0.0109	0.0274, 0.0124	0.0340, 0.0353
**completeness to Θ(full) (°)**	0.999 to 25.242	0.999 to 25.242	0.999 to 25.242
**no. of parameters, restraints**	288, 0	342, 0	297, 133
**R_1, wR_2 [F > 4σ(F)] [a]**	0.0424, 0.1090	0.0439, 0.1136	0.0465, 0.1019
**R_1, wR_2 [all data] [a]**	0.0495, 0.1140	0.0558, 0.1234	0.0665, 0.1133
**GooF**	1.033	1.025	1.030
**largest diff. Fourier peak, hole (eÅ^{-3})**	0.271, −0.214	0.292, −0.264	0.208, −0.187

Table S1 Continued. Crystal Data, Data Collection and Refinement Parameters.

data	S1	S2	S3
**CCDC number**	2486665	2486666	2486667
**formula**	$C_{32}H_{42}F_3N$	$C_{52}H_{73}F_3N_2$	$C_{35}H_{43}N$
**formula weight (g·mol^{-1})**	497.66	783.12	477.70
**colour, habit**	colourless tablet	clear colourless tablet	colourless block
**crystal size (mm)**	0.21 x 0.16 x 0.09	0.225 x 0.152 x 0.103	0.611 x 0.481 x 0.225
**crystal system**	triclinic	triclinic	monoclinic
**space group**	*P*-1 (no. 2)	*P*-1 (no. 2)	$P2_1/n$ (no. 14)
***a* (Å)**	9.5732(5)	9.9659(7)	12.1459(2)
***b* (Å)**	16.3090(11)	13.8216(9)	12.9541(2)
***c* (Å)**	18.8000(12)	17.9313(13)	18.1913(3)
***α* (°)**	102.698(6)	95.344(6)	90.0
***β* (°)**	98.715(5)	104.475(6)	94.7543(16)
***γ* (°)**	99.092(5)	106.268(6)	90.0
**V (Å^3)**	2774.1(3)	2260.1(3)	2852.37(9)
**Z**	4 [b]	2	4
**T (K)**	176(8)	173	173.50(10)
**D_c (g·cm^{-3})**	1.192	1.151	1.112
**radiation used, μ (mm^{-1})**	Cu Kα, 0.659	Cu Kα, 0.581	Mo Kα, 0.063
**F(000)**	1072	852	1040
**absorption correction**	analytical	analytical	analytical
**min-max transmission**	0.906 - 0.953	0.912 - 0.952	0.972 - 0.988
**Θ range for data collection (°)**	3.254 - 73.704	3.383 - 73.854	2.500 - 28.177
**no. of unique reflns measured, obs [F > 4σ(F)]**	10532, 7221	17000, 10483	6441, 5141
**R_{int}, R_{sigma}**	0.0349, 0.0579	0.0637, 0.0647	0.0520, 0.0292
**completeness to Θ(full) (°)**	0.979 to 67.684	0.998 to 67.684	0.999 to 25.242
**no. of parameters, restraints**	665, 0	562, 230	349, 89
**R_1, wR_2 [F > 4σ(F)] [a]**	0.0498, 0.1190	0.0596, 0.1547	0.0509, 0.1211
**R_1, wR_2 [all data] [a]**	0.0786, 0.1363	0.0960, 0.1710	0.0673, 0.1322
**GooF**	1.009	0.969	1.023
**largest diff. Fourier peak, hole (eÅ^{-3})**	0.365, −0.318	0.263, −0.304	0.326, −0.218

Table S1 Continued. Crystal Data, Data Collection and Refinement Parameters.

The X-ray structure of 2

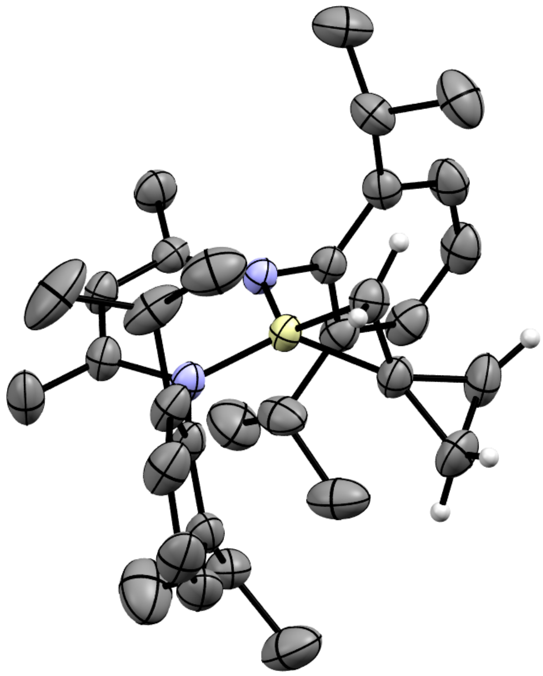

Figure S2. X-ray structure of **2**. Most hydrogen atoms and atoms are hidden for clarity, thermal ellipsoids are at the 50 % probability level.

The X-ray crystal structure of 3

The crystal was found to be a two-component non-merohedral twin at a ratio of 0.51:0.49 with the two components related by the approximate twin law of [-1.00 0.00 0.00 0.53 -1.00 0.00 0.00 0.00 -1.00].

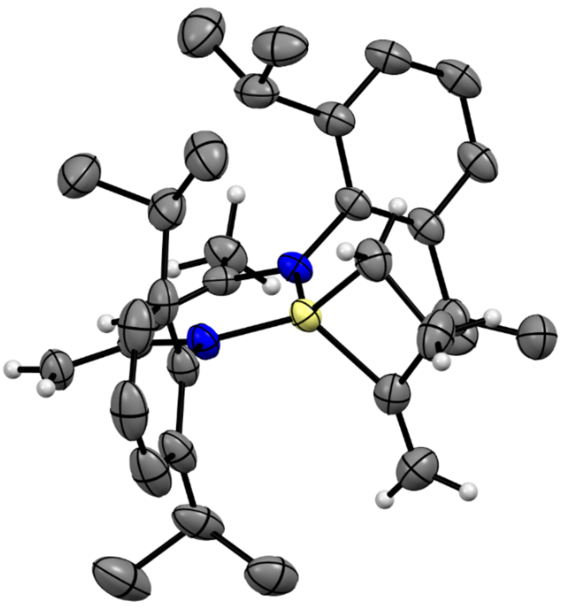

Figure S3. X-ray structure of **3**, showing one of the crystallographically independent molecules in the asymmetric unit. Most hydrogen atoms and atoms are hidden for clarity, thermal ellipsoids are at the 50 % probability level.

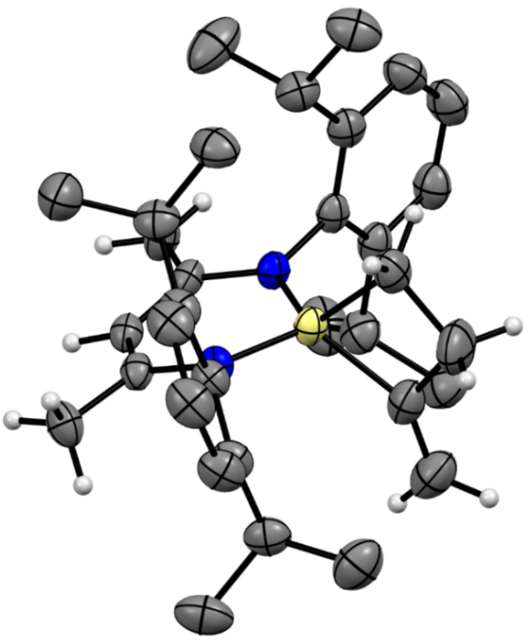

Figure S4. X-ray structure of **3**, showing the second crystallographically independent molecules in the asymmetric unit. Most hydrogen atoms and atoms are hidden for clarity, thermal ellipsoids are at the 50 % probability level.

The X-ray crystal structure of 4

The two fused cyclopropane rings were found to be disordered together. They were modelled in two orientations at a ratio of ca. 0.87:0.13, including the C1 of the CAAC. The two orientations were restrained to be similar and only the non-hydrogen atoms of the major orientations were modelled anisotropically, the rest were modelled isotropically.

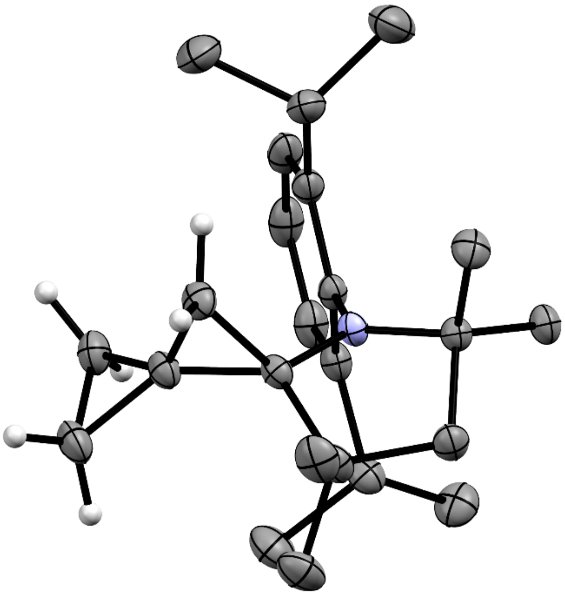

Figure S5. X-ray structure of **4**. Most hydrogen atoms and atoms of the minor occupancy orientation are hidden for clarity, thermal ellipsoids are at the 50 % probability level.

X-ray crystal structure of 5b

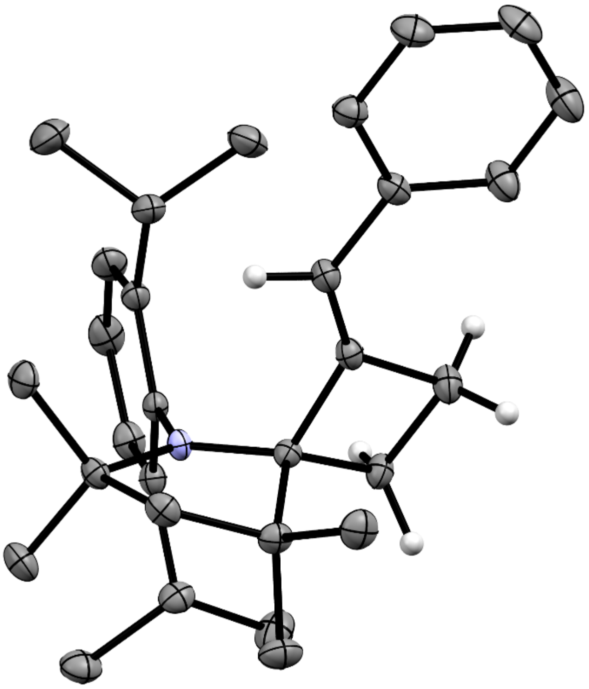

Figure S6. X-ray structure of **5b**. Most hydrogen atoms are hidden for clarity, thermal ellipsoids are at the 50 % probability level.

The X-ray crystal structure of 5c

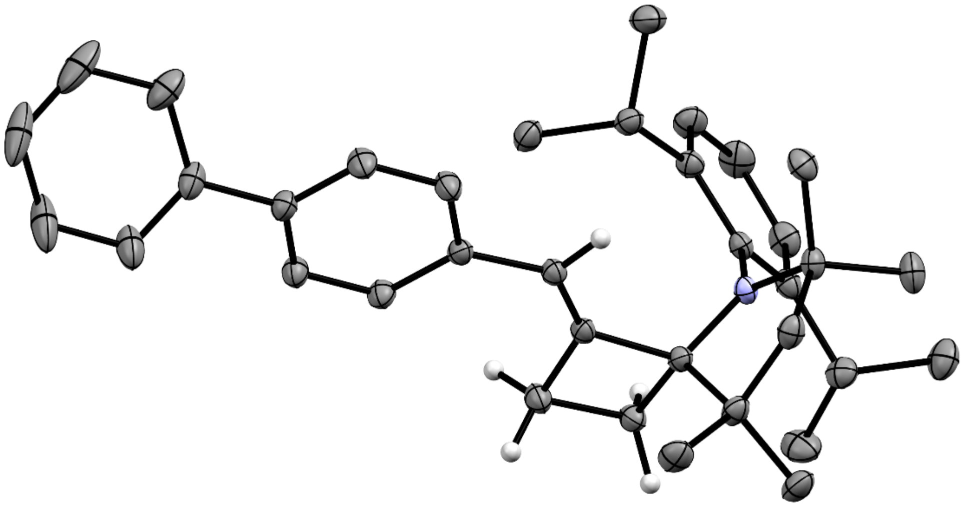

Figure S7. X-ray structure of **5c**. Most hydrogen atoms are hidden for clarity, thermal ellipsoids are at the 50 % probability level.

The X-ray crystal structure of 5d

The furan fragment was found to be disordered. It was modelled in two orientations at a ratio of ca. 0.74:0.26. The two orientations were restrained to be similar and only the non-hydrogen atoms of the major orientations were modelled anisotropically, the rest were modelled isotropically.

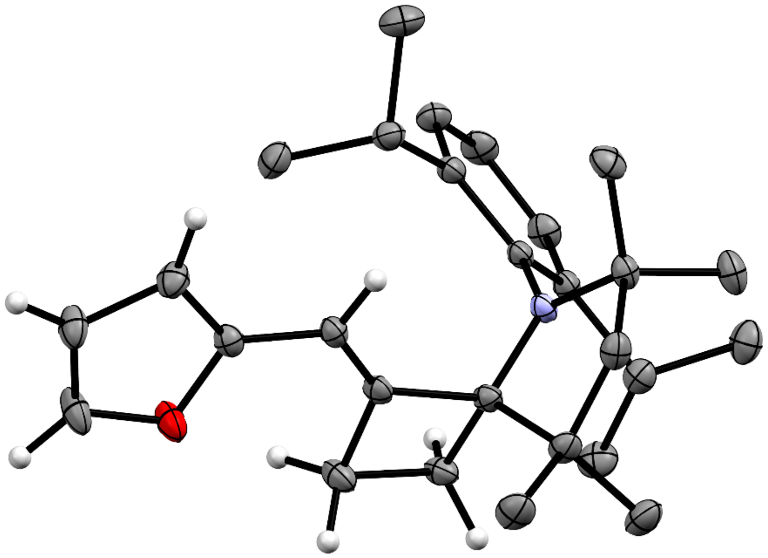

Figure S8. X-ray structure of **5d**. Most hydrogen atoms and atoms of the minor occupancy orientation are hidden for clarity, thermal ellipsoids are at the 50 % probability level

The X-ray crystal structure of S1

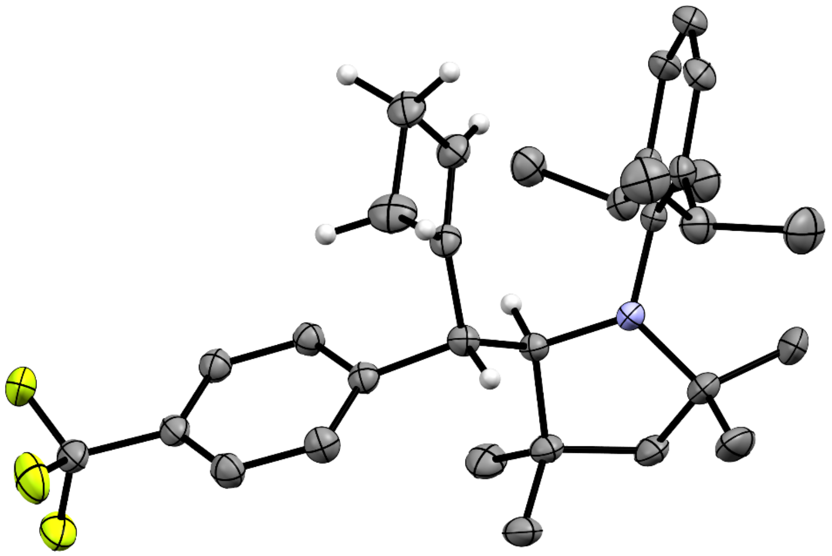

Figure S9. X-ray structure of **S1**, showing one of the crystallographically independent molecules in the asymmetric unit. Most hydrogen atoms and atoms and the second independent molecule in the asymmetric unit are hidden for clarity, thermal ellipsoids are at the 50 % probability level.

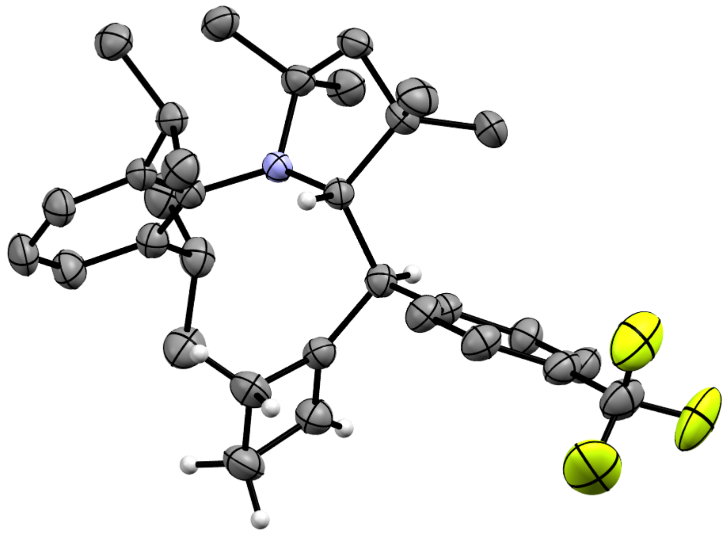

Figure S10. X-ray structure of **S1**. Most hydrogen atoms and atoms and the second independent molecule in the asymmetric unit are hidden for clarity, thermal ellipsoids are at the 50 % probability level.

The X-ray crystal structure of S2

The crystal was found to be a two-component non-merohedral twin at a ratio of ca. 0.53:0.47 with the two components related by the approximate twin law of [-1.00 0.01 0.01 0.00 -1.00 0.01 0.10 0.48 0.99]. The C42-based $C(CH_3)_2$ fragment one the C26-based CAAC was found to be disordered. It was modelled in two orientations at a ratio of ca. 0.94:0.06, including C42. The C54-based CF_3 fragment was found to be disordered. It was modelled in two orientations at a ratio of ca. 0.82:0.18, including the C54. For both of the above disorders, the two orientations were restrained to be similar and only the non-hydrogen atoms of the major orientations were modelled anisotropically, the rest were modelled isotropically.

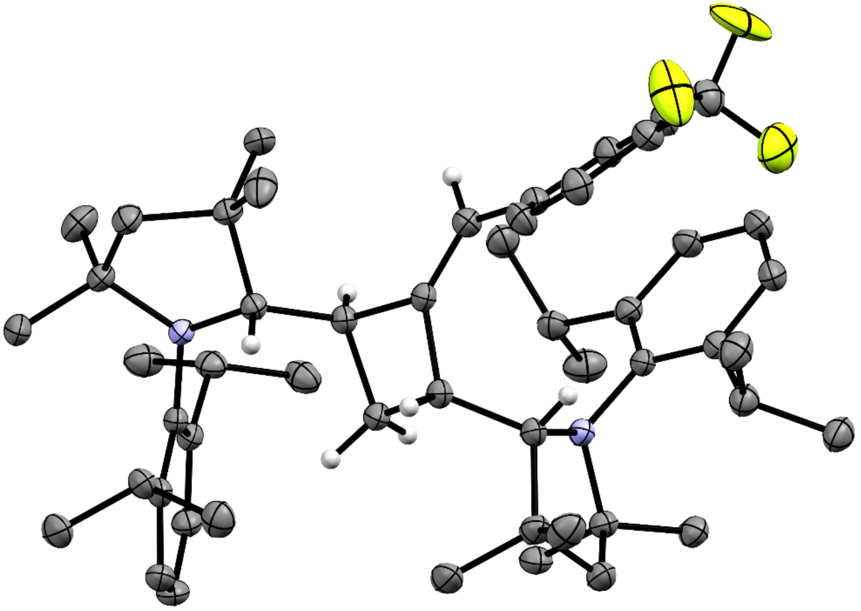

Figure S11. X-ray structure of **S2**, showing the second crystallographically independent molecules in the asymmetric unit. Most hydrogen atoms and atoms and atoms of the minor occupancy orientations are hidden for clarity, thermal ellipsoids are at the 50 % probability level.

The X-ray crystal structure of S3

The C30-based $C(CH_3)_2$ fragment one the CAAC was found to be disordered, including C30. It was modelled in two orientations at a ratio of ca. 0.73:0.27. The two orientations were restrained to be similar and only the non-hydrogen atoms of the major orientations were modelled anisotropically, the rest were modelled isotropically.

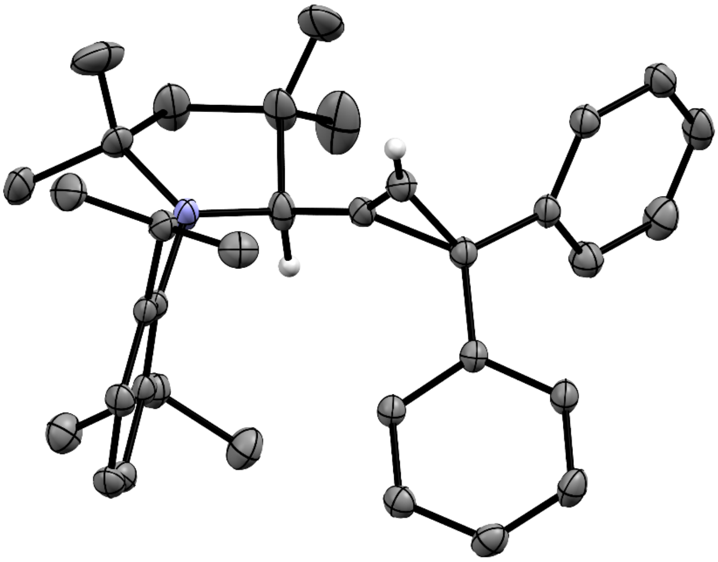

Figure S12. X-ray structure of **S3**. Most hydrogen atoms and atoms and atoms of the minor occupancy orientations are hidden for clarity, thermal ellipsoids are at the 50 % probability level.

6) DFT Studies

6.1. Computational methods

DFT calculations were performed using Gaussian 16 (Revision C.01) using an ultrafine integration grid (int=ultrafine).[S13] Geometry optimisations and frequency calculations were performed using the BP86[S14-S15] density functionals including solvent (CPCM, benzene, ε = 2.2706) and dispersion corrections (D3) using the def2-SVP (C, H) and def2-TZVPP (N, F, Si) hybrid basis set.[S16-S19] Frequency analyses for all stationary points were performed using the enhanced criteria to confirm the nature of the structures as either minima (no imaginary frequency) or transition states (only one imaginary frequency). The electronic energies of the optimised geometries were calculated using the BP86 functional including dispersion corrections (D3) with def2-TZVPP basis sets for all atoms with solvent corrections (CPCM, benzene, ε = 2.2706). [S17-S20]

Intrinsic reaction coordinate (IRC) [S21] calculations were used to connect transition states and minima located on the potential energy surface allowing a full energy profile (calculated at 298.15 K, 1 atm.) of the reaction to be constructed. Temperature (373 K for **2** to **3**; 333 K for **4** to **5a**;) and concentration (0.14 M) corrections were performed on the reaction pathway using GoodVibes v3.2. [S22]

NBO analysis was performed at the BP86/def2-TZVPP level for all atoms with dispersion corrections (D3) and solvent corrections (CPCM, benzene, ε = 2.2706) by using NBO 7.0.[S23-S24]

6.2. Calculated Reaction Pathway and Optimised Structures

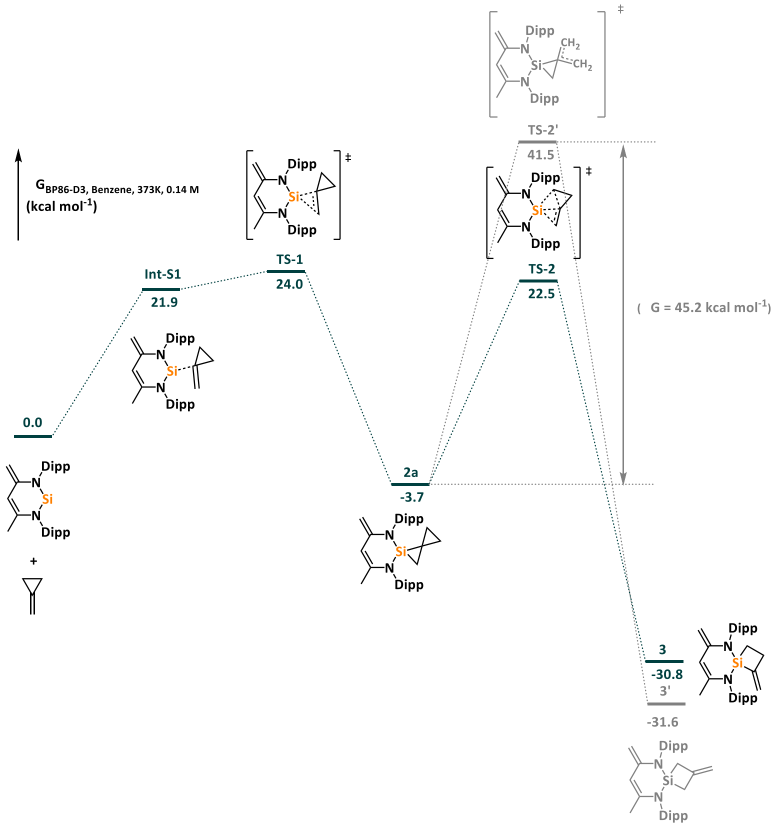

Figure S13. Proposed pathway for reversible ligand coordination based on DFT calculations G16: BP86-D3 / def2-TZVPP / CPCM (benzene) // BP86-D3 / def2-SVP (C, H) / def2-TZVPP (N, Si) / CPCM (benzene). Gibbs energies, values in kcal mol^{-1}. Temperature (373 K) and concentration correction (0.14 M) were performed on the reaction pathway using GoodVibes v3.2.

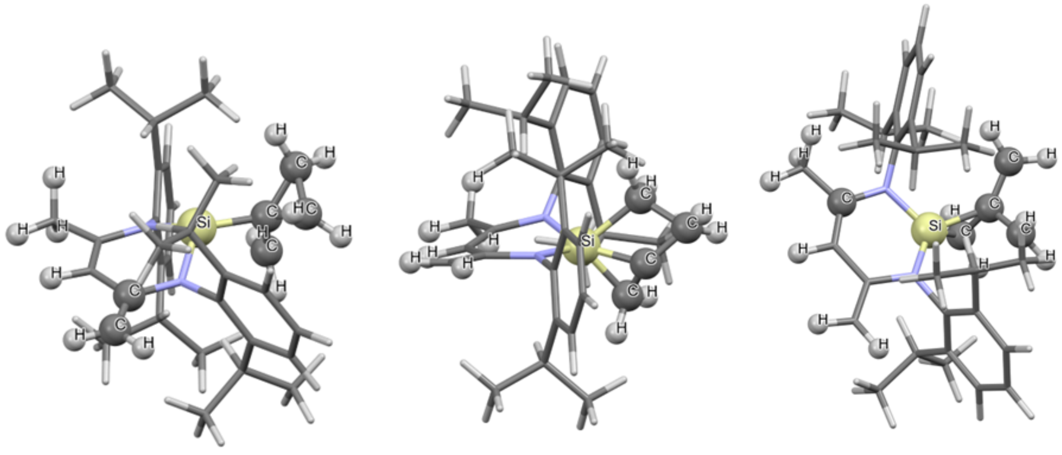

TS-1 TS-2 TS-2'

Figure S14. Optimised structures of key transition states and intermediates.

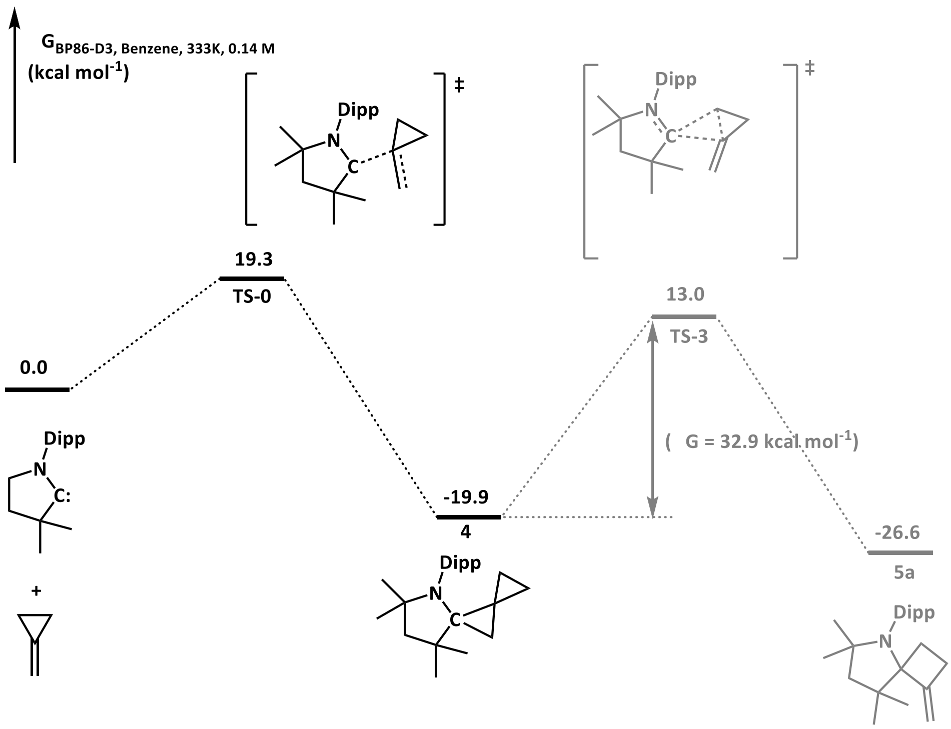

Figure S15. Proposed pathway for reversible ligand coordination based on DFT calculations G16: BP86-D3 / def2-TZVPP / CPCM (benzene) // BP86-D3 / def2-SVP (C, H) / def2-TZVPP (N) / CPCM (benzene). Gibbs energies, values in kcal mol^{-1}. Temperature (333 K) and concentration correction (0.14 M) were performed on the reaction pathway using GoodVibes v3.2.

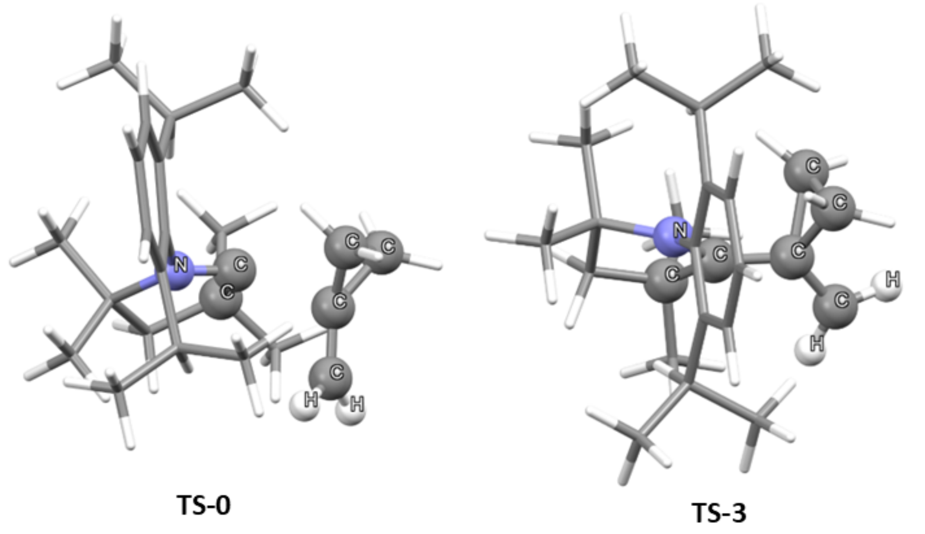

Figure S16. Optimised structures of key transition states and intermediates.

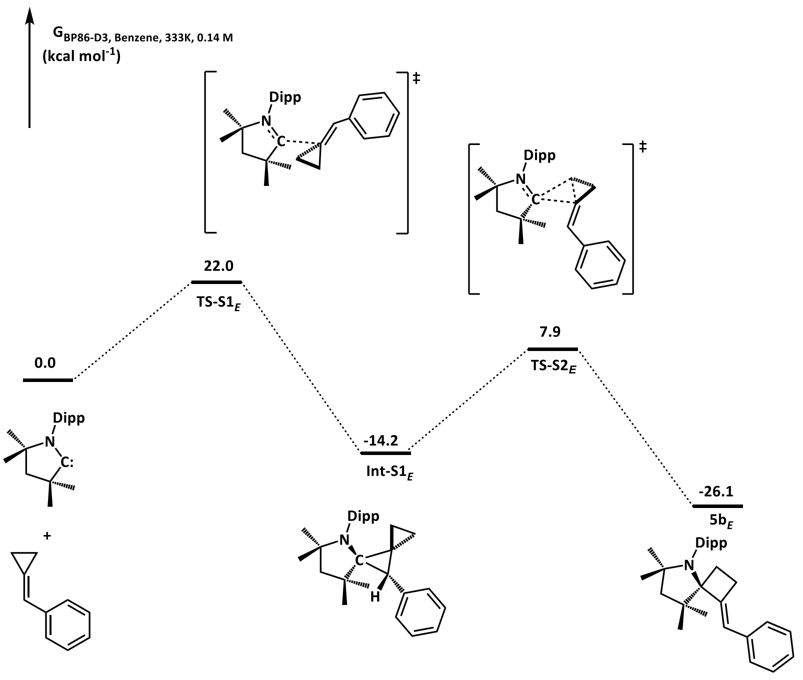

Figure S17. Proposed pathway for reversible ligand coordination based on DFT calculations G16: BP86-D3 / def2-TZVPP/ CPCM (benzene) // BP86-D3 / def2-SVP (C, H) / def2-TZVPP (N) / CPCM (benzene). Gibbs energies, values in kcal mol^{-1}. Temperature (333 K) and concentration correction (0.14 M) were performed on the reaction pathway using GoodVibes v3.2.

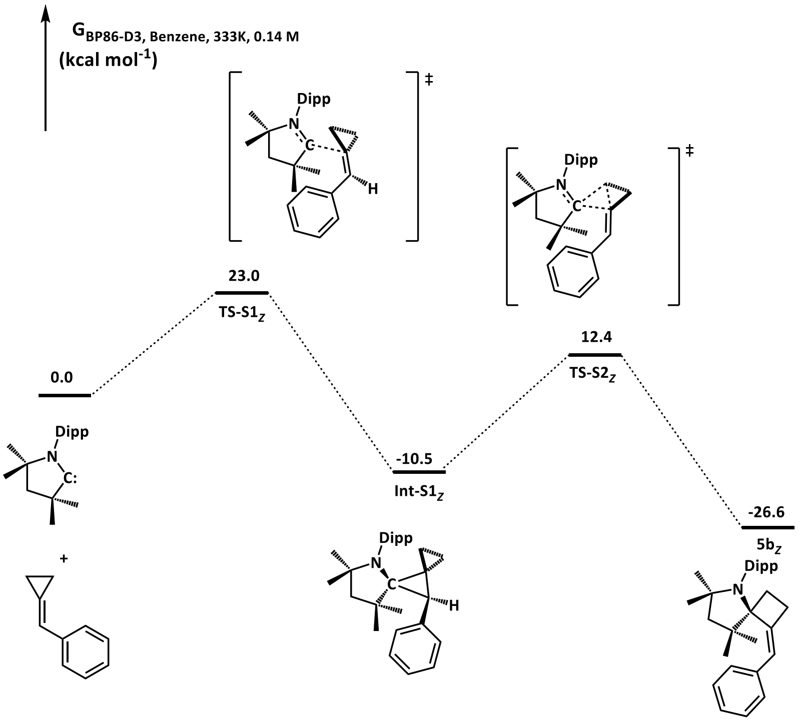

Figure S18. Proposed pathway for reversible ligand coordination based on DFT calculations G16: BP86-D3 / def2-TZVPP/ CPCM (benzene) // BP86-D3 / def2-SVP (C, H) / def2-TZVPP (N) / CPCM (benzene). Gibbs energies, values in kcal mol^{-1}. Temperature (333 K) and concentration correction (0.14 M) were performed on the reaction pathway using GoodVibes v3.2.

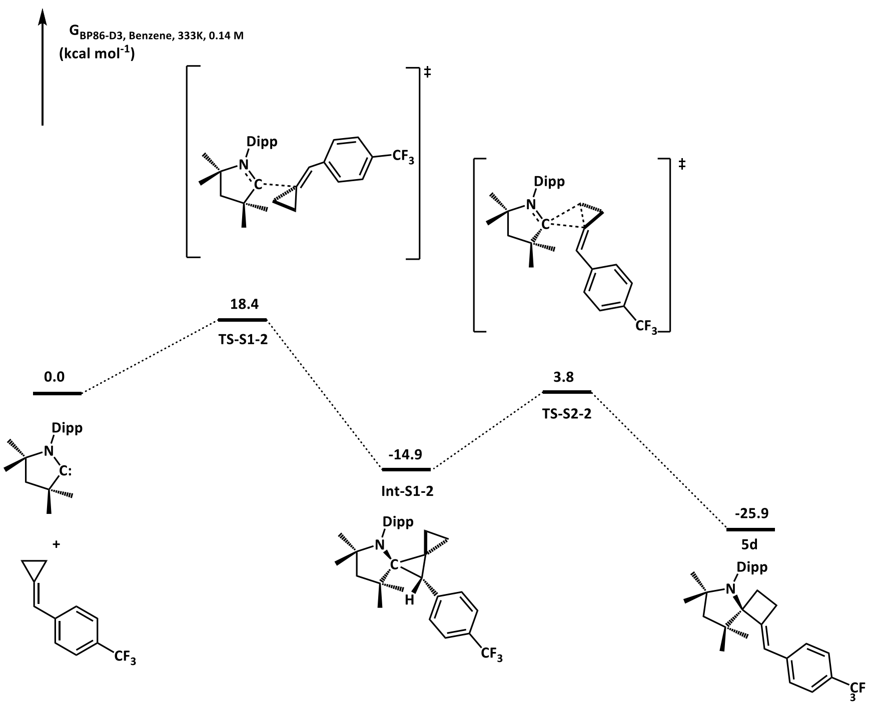

Figure S19. Proposed pathway for reversible ligand coordination based on DFT calculations G16: BP86-D3 / def2-TZVPP/ CPCM (benzene) // BP86-D3 / def2-SVP (C, H) / def2-TZVPP (N, F) / CPCM (benzene). Gibbs energies, values in kcal mol^{-1}. Temperature (333 K) and concentration correction (0.14 M) were performed on the reaction pathway using GoodVibes v3.2.

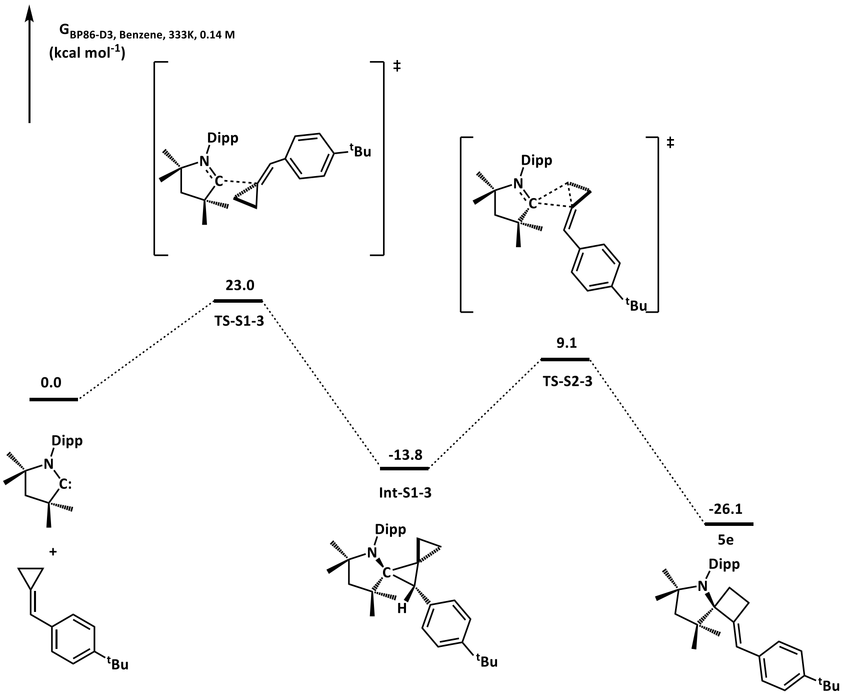

Figure S20. Proposed pathway for reversible ligand coordination based on DFT calculations G16: BP86-D3 / def2-TZVPP / CPCM (benzene) // BP86-D3 / def2-SVP (C, H) / def2-TZVPP (N) / CPCM (benzene). Gibbs energies, values in kcal mol^{-1}. Temperature (333 K) and concentration correction (0.14 M) were performed on the reaction pathway using GoodVibes v3.2.

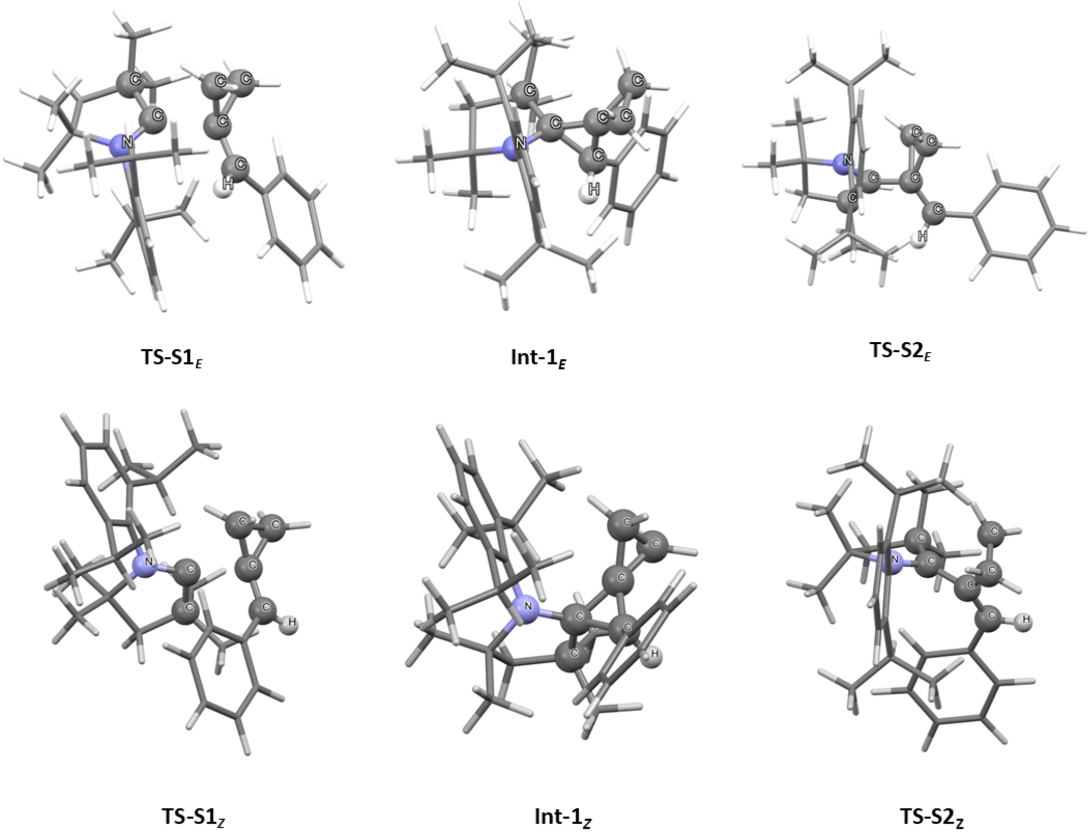

Figure S21. Optimised structures of key transition states and intermediates.

6.3. NBO Analysis

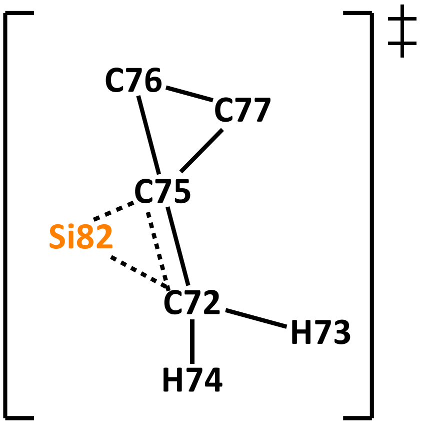

TS-1

Atom	Charge
Si82	1.64
C72	-0.53
C75	-0.50
C76	-0.40
C77	-0.42
H73	0.22
H74	0.21

Table S2. NPA change data for **TS-1**.

Wiberg bond index	
Si82 - C72	0.620
Si82 - C75	0.695
C75 - C76	0.945
C75 - C77	0.944
C75 - C72	1.202
C76 - C77	0.991
C72 - H73	0.918
C72 - H74	0.919

Table S3. WBI data for **TS-1**.

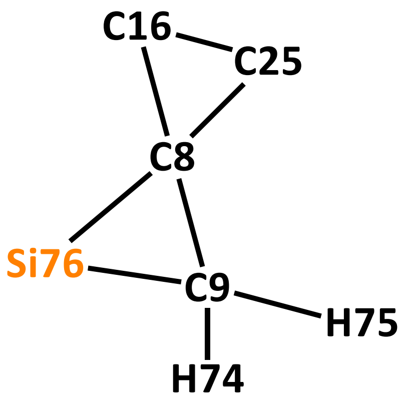

2

Atom	Charge
Si76	1.96
C8	-0.53
C9	-0.89
C16	-0.41
C25	-0.40
H74	0.25
H75	0.24

Table S4. NPA change data for **2**.

Wiberg bond index	
Si76 - C8	0.748
Si76 - C9	0.743
Si76 - C25	0.020
C8 - C9	1.011
C8 - C16	0.964
C8 - C25	0.960
C16 - C25	1.002
C9 - H74	0.900
C9 - H75	0.903

Table S5. WBI data for **2**.

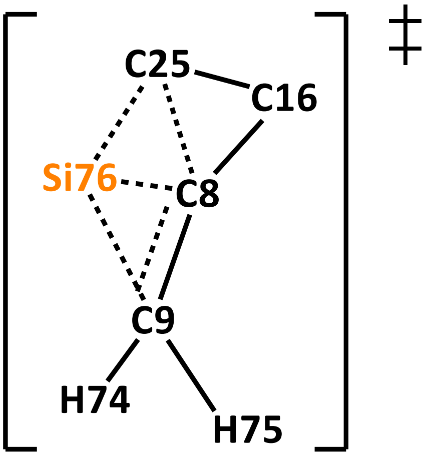

TS-2

Atom	Charge
Si76	1.95
C8	-0.24
C9	-0.89
C16	-0.44
C25	-0.67
H74	0.25
H75	0.21

Table S6. NPA change data for **TS-2**.

Wiberg bond index	
Si76 - C8	0.528
Si76 - C9	0.633
Si76 - C25	0.453
C8 - C9	1.138
C8 - C16	1.090
C8 - C25	0.521
C16 - C25	0.911
C9 - H74	0.879
C9 - H75	0.891

Table S7. WBI data for **TS-2**.

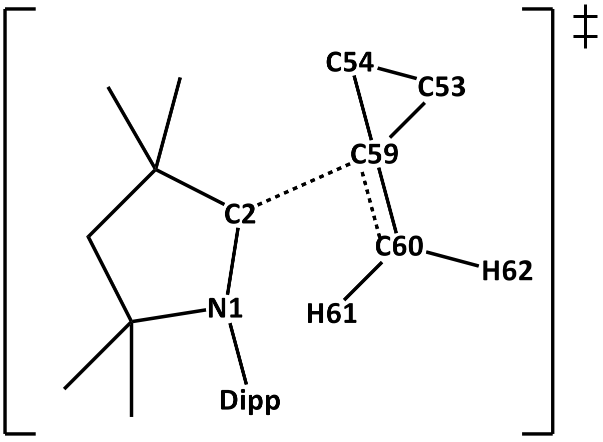

TS-0

Atom	Charge
N1	-0.46
C2	0.27
C53	-0.46
C54	-0.46
C59	0.01
C60	-0.48
H61	0.20
H62	0.20

Table S8. NPA change data for **TS-0**.

Wiberg bond index	
C2 - C59	0.27
C53 - C54	0.96
C53 - C59	1.00
C54 - C59	1.00
C59 - C60	1.68
C60 - H61	0.92
C60 - H62	0.92

Table S9. WBI data for **TS-0**.

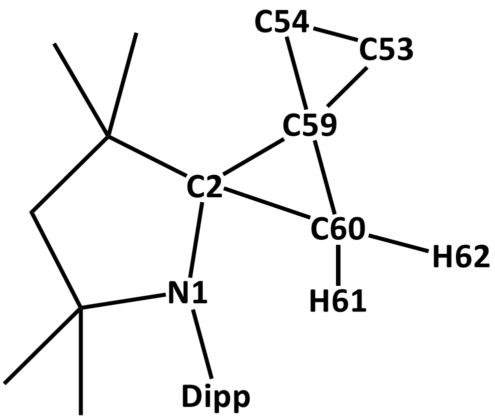

4

Atom	Charge
N1	-0.48
C2	0.18
C53	-0.42
C54	-0.41
C59	-0.11
C60	-0.45
H61	0.22
H62	0.22

Table S10. NPA change data for **4**.

Wiberg bond index	
C2 - C59	0.97
C53 - C54	0.98
C53 - C59	0.98
C54 - C59	0.98
C59 - C60	0.97
C60 - H61	0.92
C60 - H62	0.91

Table S11. WBI data for **4**.

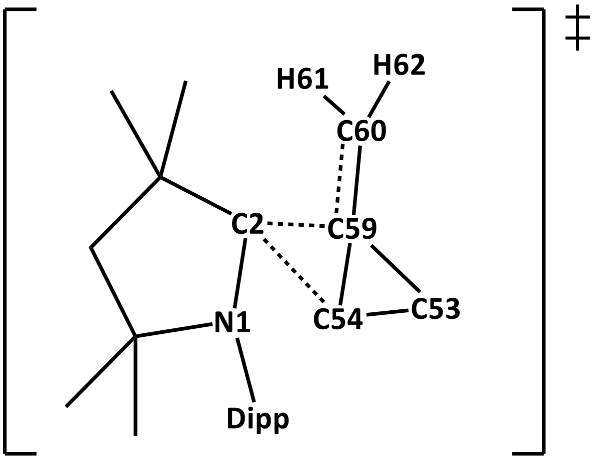

TS-3

Atom	Charge
N1	-0.41
C2	0.36
C53	-0.43
C54	-0.32
C59	-0.14
C60	-0.73
H61	0.20
H62	0.19

Table S12. NPA change data for **TS-3**.

Wiberg bond index	
C2 - C59	1.086
C53 - C54	1.040
C53 - C59	0.875
C54 - C59	0.739
C59 - C60	1.186
C60 - H61	0.935
C60 - H62	0.941

Table S13. WBI data for **TS-3**.

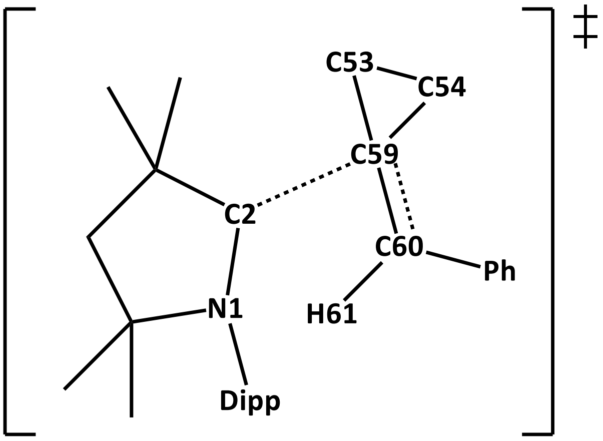

TS-3

Atom	Charge
N1	-0.43
C2	0.46
C53	-0.46
C54	-0.45
C59	0.04
C60	-0.40
H61	0.20

Table S14. NPA change data for **TS-3**.

Wiberg bond index	
C2 - C59	0.45
C2 - C60	0.20
C53 - C54	0.97
C53 - C59	0.99
C54 - C59	0.98
C59 - C60	1.41
C60 - H61	0.90

Table S15. WBI data for **TS-3**.

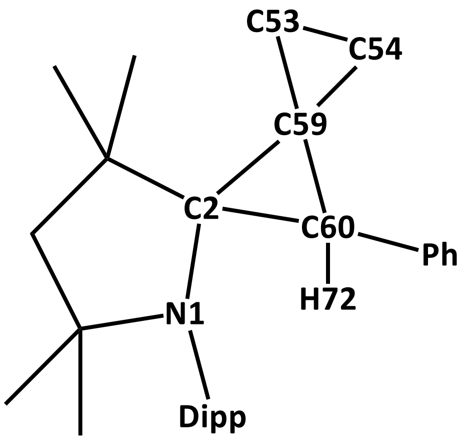

Int-S1$_E$

Atom	Charge
N1	-0.48
C2	0.22
C53	-0.41
C54	-0.42
C59	-0.11
C60	-0.27
H72	0.23

Table S16. NPA change data for **Int-1$_E$**.

Wiberg bond index	
C2 - C59	0.97
C2 - C60	0.82
C53 - C54	0.98
C53 - C59	0.967
C54 - C59	0.97
C59 - C60	0.95
C60 - H72	0.89

Table S17. WBI data for **Int-1$_E$**.

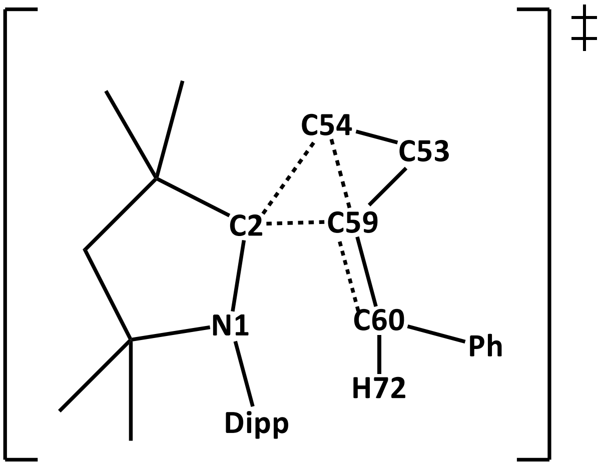

TS-S2$_E$

Atom	Charge
N1	-0.43
C2	0.41
C53	-0.48
C54	-0.33
C59	-0.07
C60	-0.42
H72	0.22

Table S18. NPA change data for **TS-S2$_E$**.

Wiberg bond index	
C2 - C59	1.09
C2 - C54	0.27
C53 - C54	1.01
C53 - C59	0.94
C54 - C59	0.53
C59 - C60	1.27
C60 - H72	0.90

Table S19. WBI data for **TS-S2$_E$**.

7) Kinetic Analysis

7.1. Kinetics Experiments

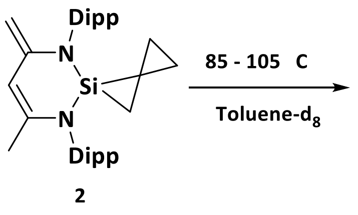

Reactions were monitored in situ in a Bruker 500 MHz machine. The formation of **3** was monitored by quantitative ^{1}H-NMR spectroscopy in the presence of an internal standard (internal standard: 1,3,5-trimethoxybenzene in d_8-toluene, concentration: 0.01 M).

In a N_2 filled glovebox, a 0.024 M stock solution of **2** (30.0 mg, 0.06 mmol) was prepared in toluene-d_8 (2.50 mL), and aliquoted into five J Young NMR tubes with 1,3,5-trimethoxybenzene (3.0 mg, 0.02 mmol) as the internal standard. After that, the NMR tubes were inserted into a preheated ^{1}H NMR spectrometer (358 – 378 K). (ln([**2**]/[**2**]$_0$ of the test data is selected for analysis and calculation.) Five rate constants were determined in the temperature range (358 – 378K) using the initial rates method, by determining the gradient in the linear section of a concentration vs time plot for each temperature (data was plotted to 90 % conversion). A plot of ln([**2**]/[**2**]$_0$) against 1/T allowed calculation of the thermodynamic parameters using the Eyring equation.

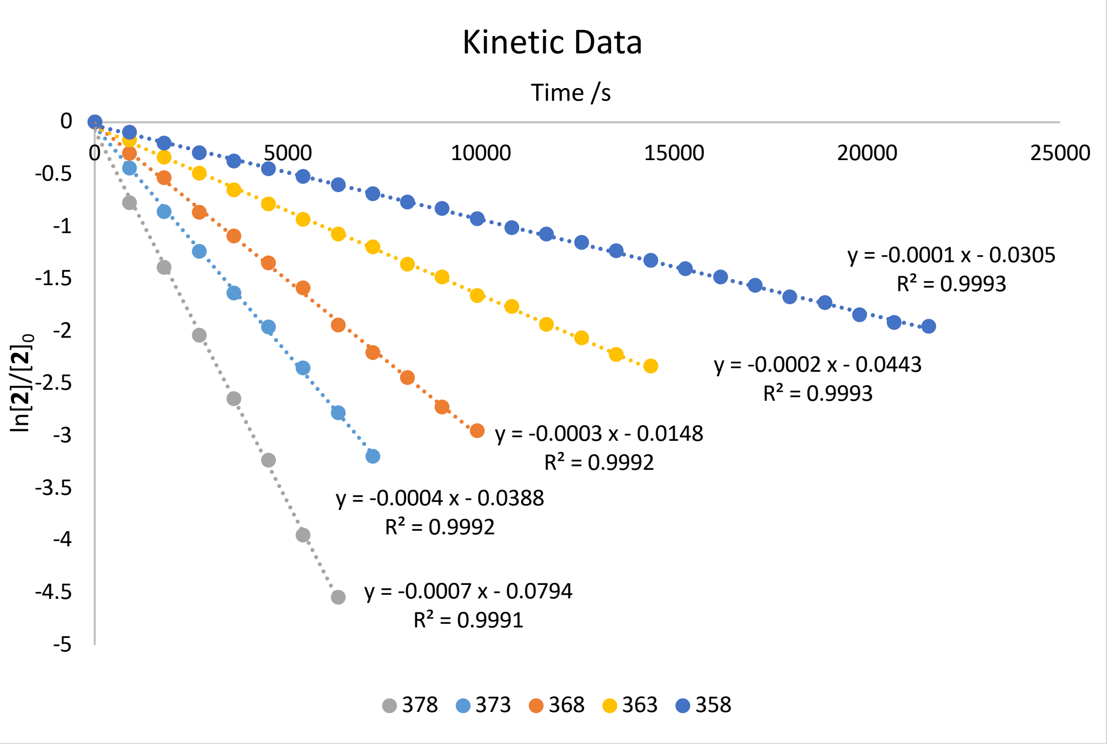

Figure S22. Kinetic data for the transformation of **2** to **3** at temperatures 358 – 378 K (5K intervals).

Plotting ln[**2**] (concentration determined by the integration of the internal 1,3,5-trimethoxybenzene standard) against time showed a linear relationship indicating that the reaction is first order in [**2**]. Four rate constants were determined in the temperature range of 358 – 378 K at 5°C intervals and a plot of $\ln(k_{obs})$ against 1/T allowed calculation of the thermodynamic parameters using the Eyring equation. The activation parameters for the reaction were found to be: $\Delta H^‡$ = +26.9 kcal mol^{-1}, $\Delta S^‡$ = –2.2 cal K^{-1} mol^{-1} and an associated $\Delta G^‡_{298K}$ = 27.6 kcal mol^{-1} ($\Delta G^‡_{373K}$ = 27.7 kcal mol^{-1}).

**T/K**	**1/T**	**K_{obs} (s^{-1})**	**ln(k/T)**
358	2.79E-03	9.01E-05	-1.520E+01
363	2.75E-03	1.61E-04	-1.463E+01
368	2.72E-03	3.00E-04	-1.402E+01
373	2.68E-03	4.35E-04	-1.366E+01
378	2.65E-03	7.12E-04	-1.318E+01

Table S20. Table of constants derived from kinetic experiments.

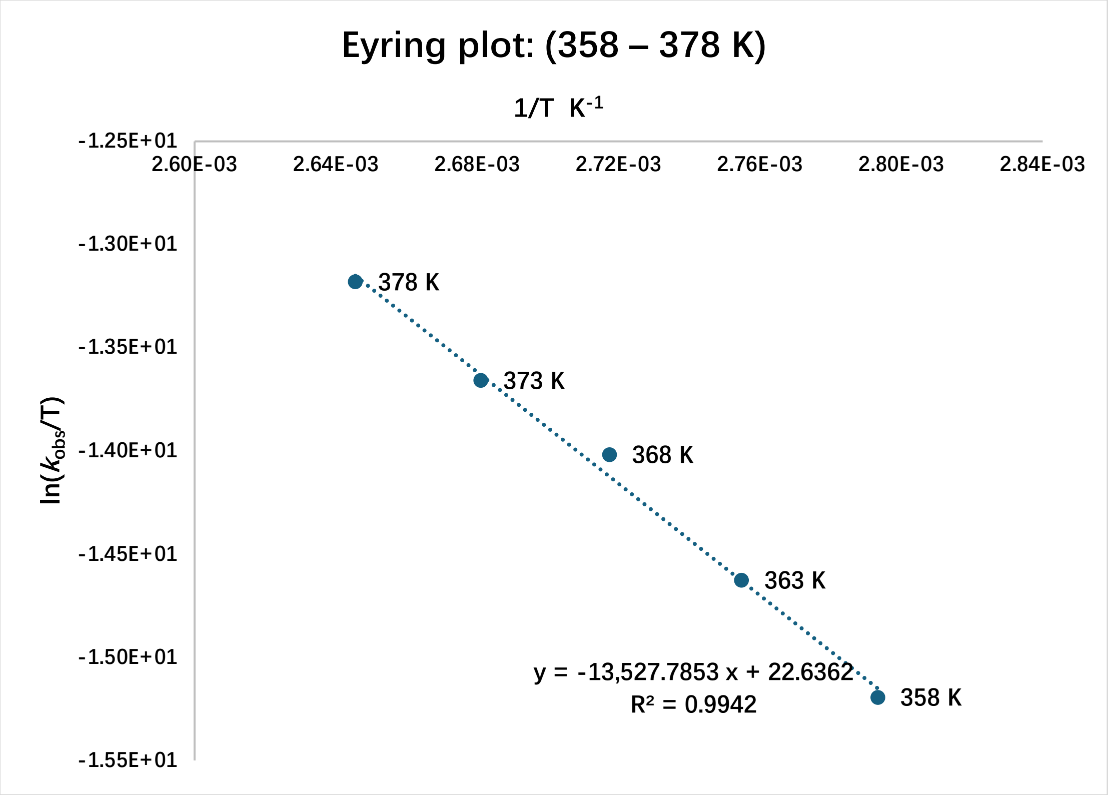

Figure S23. Eyring plot of data from Table S20

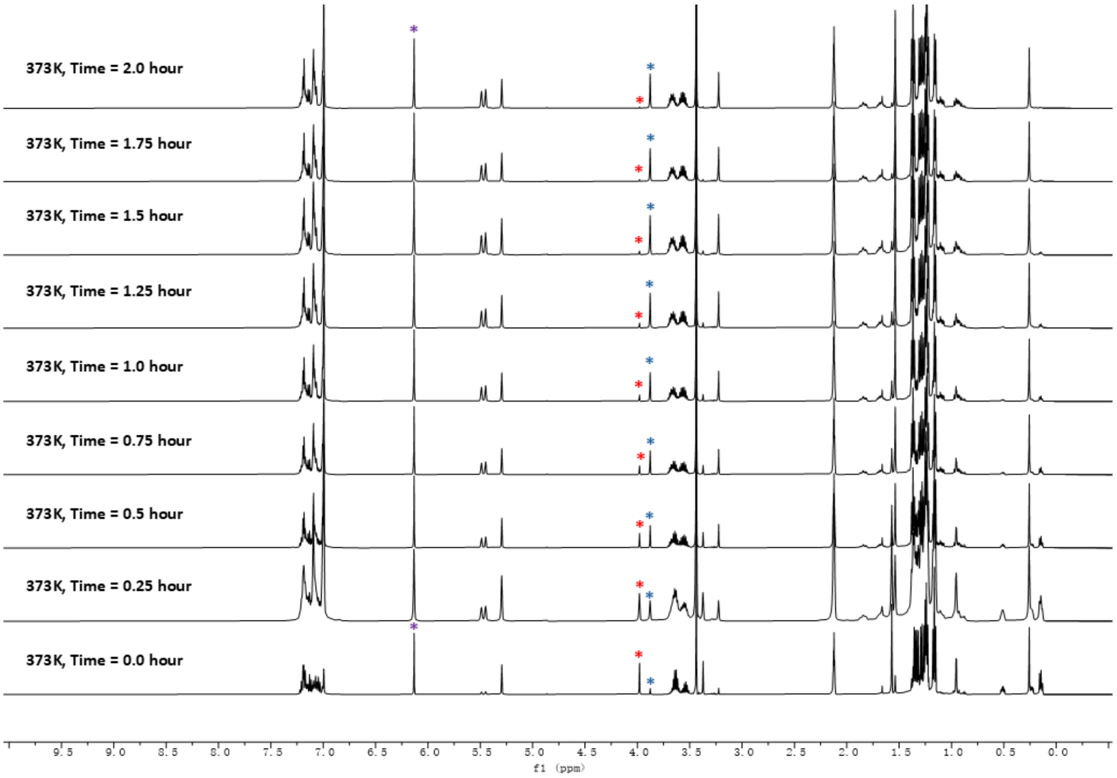

Figure S24. Selected ^{1}H NMR spectra from kinetic experiments at 373K. ("*" is the peak of internal 1,3,5-trimethoxybenzene standard, "*" is the peak of starting material **2**, "*" is the peak of product **3**.)

8) NMR Spectroscopy

8.1 NMR Spectra of Isolated products

^{1}H NMR (400 MHz, 298 K, C_6D_6) of **2**:

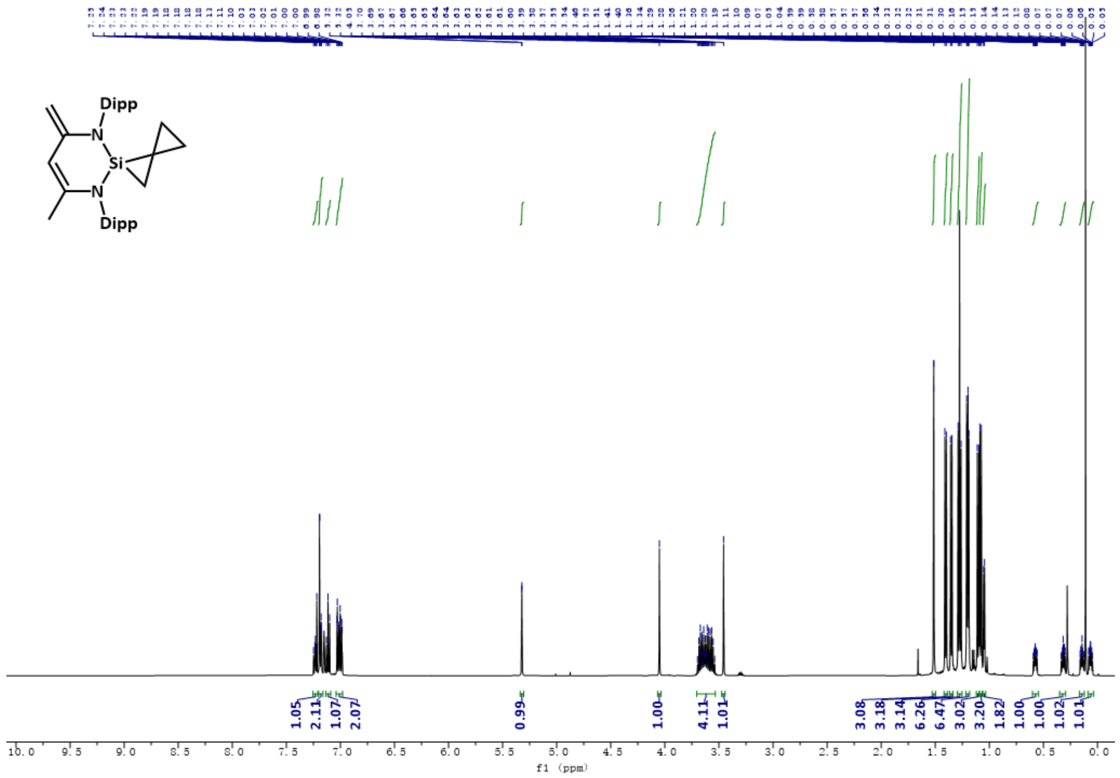

^{29}Si{^{1}H} NMR (99 MHz, 298K, C_6D_6) of **2**:

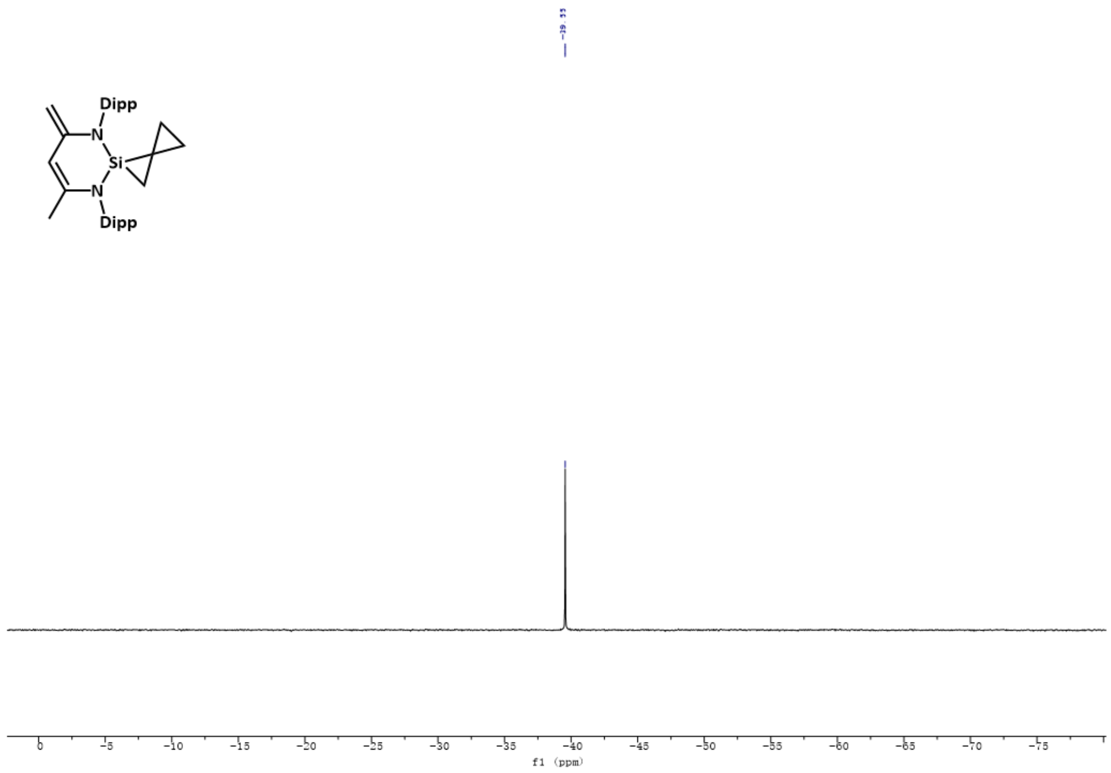

$^{13}C\{^1H\}$ **NMR** (101 MHz, 298 K, C_6D_6) of **2**:

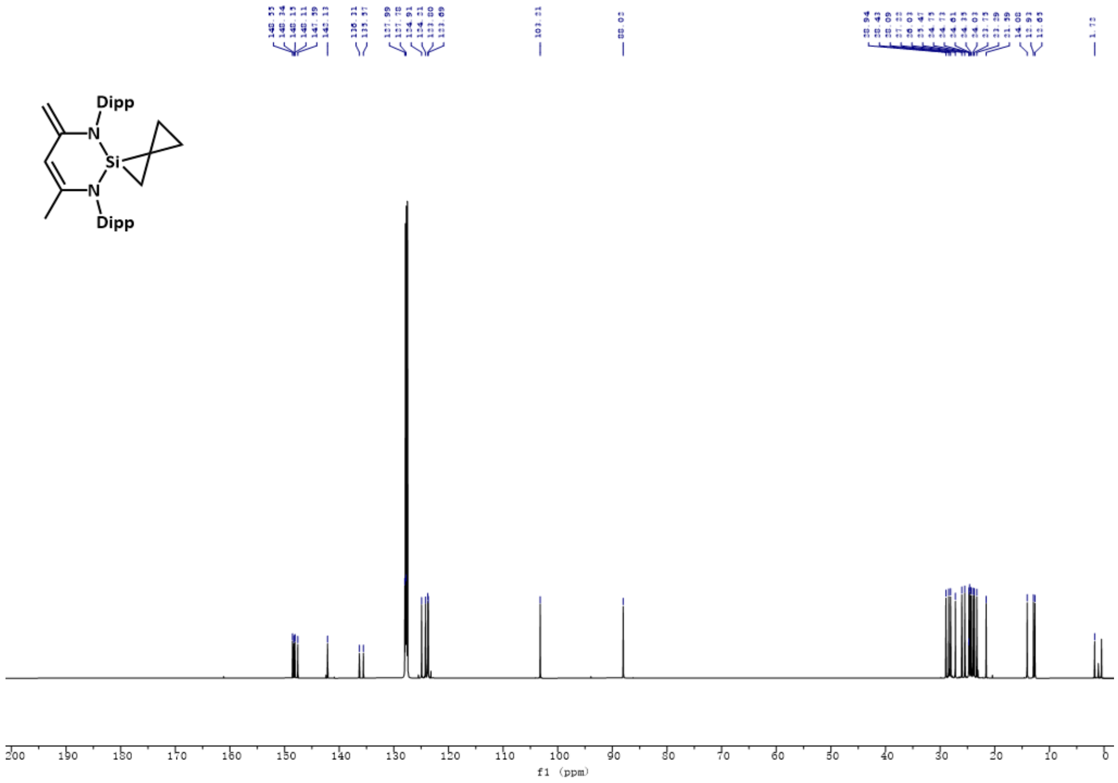

^1H **NMR** (400 MHz, 298 K, C_6D_6) of **3**:

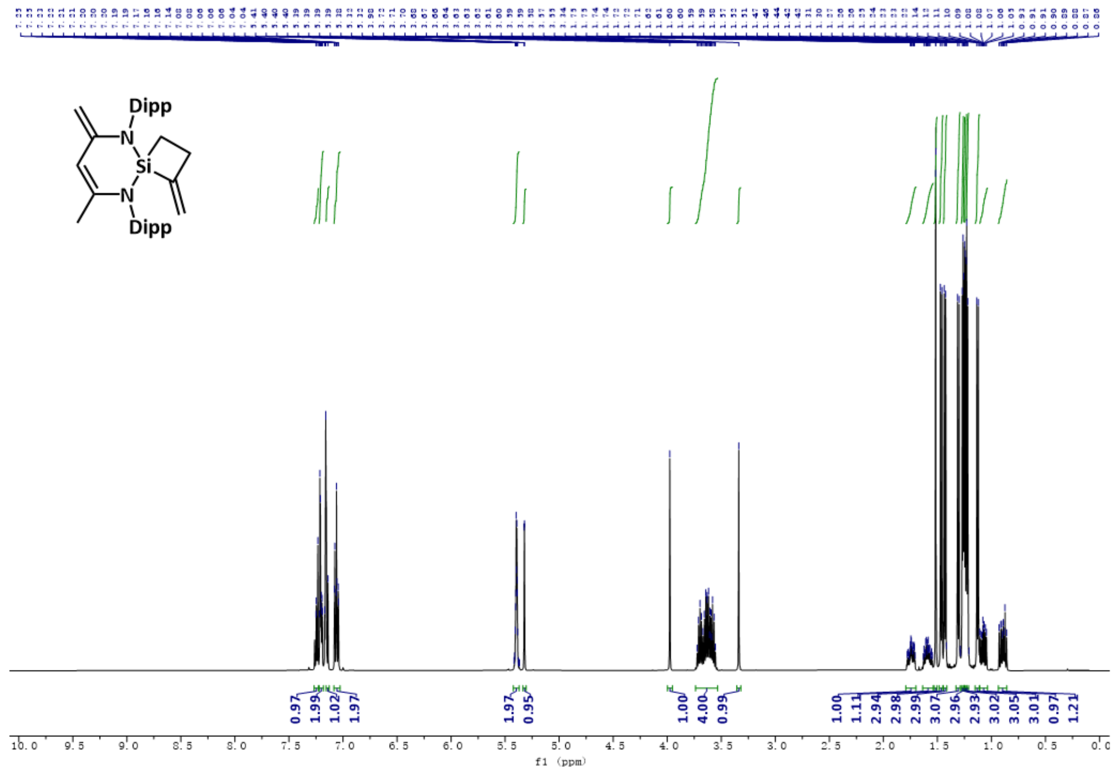

$^{29}Si\{^1H\}$ NMR (99 MHz, 298K, C_6D_6) of **3**:

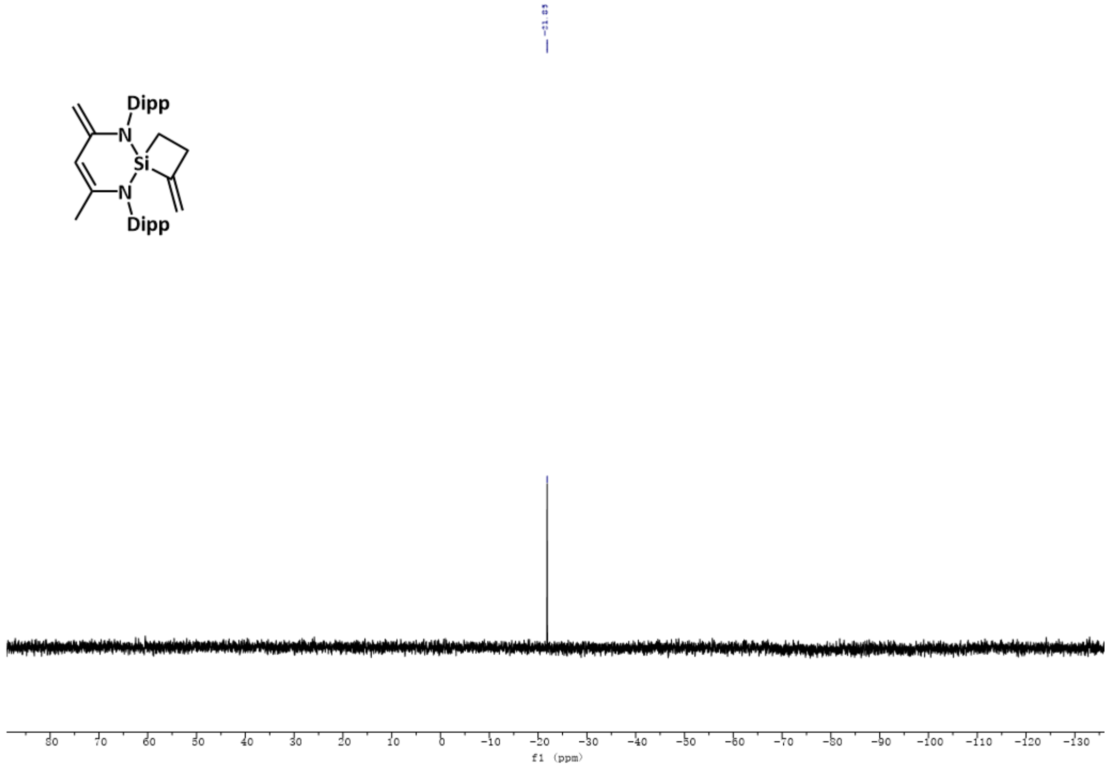

$^{13}C\{^1H\}$ NMR (101 MHz, 298 K, C_6D_6) of **3**:

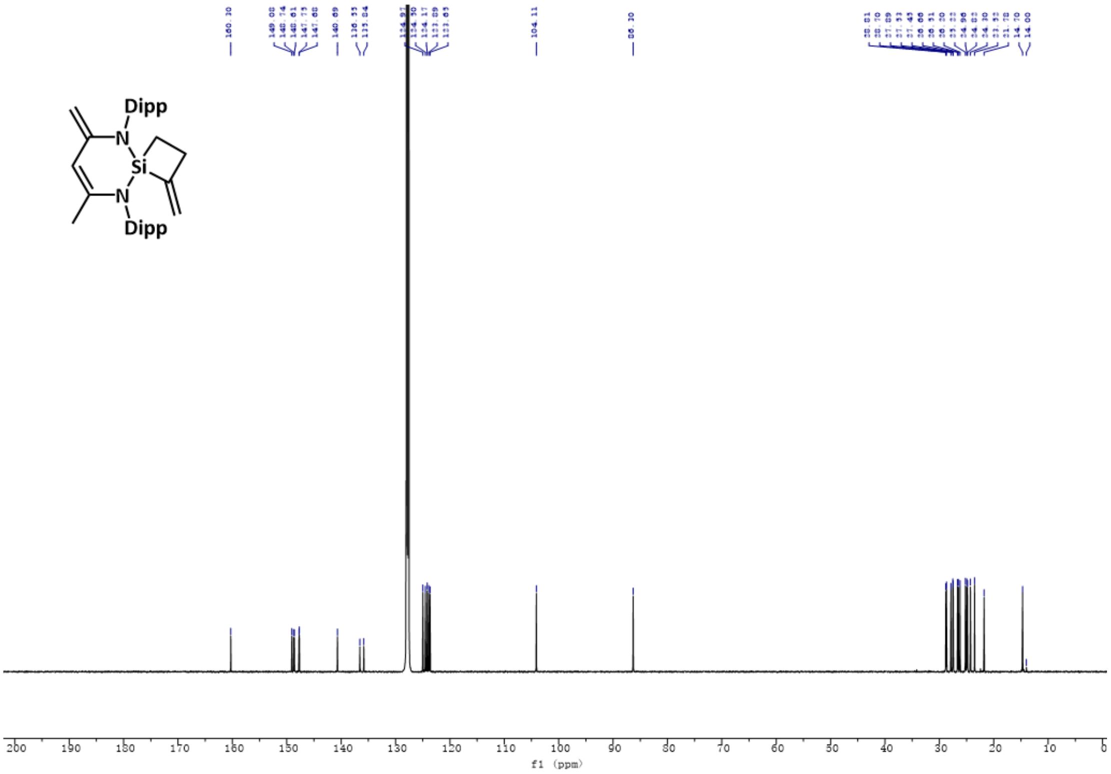

^{1}H NMR (400 MHz, 298 K, C_6D_6) of **4**:

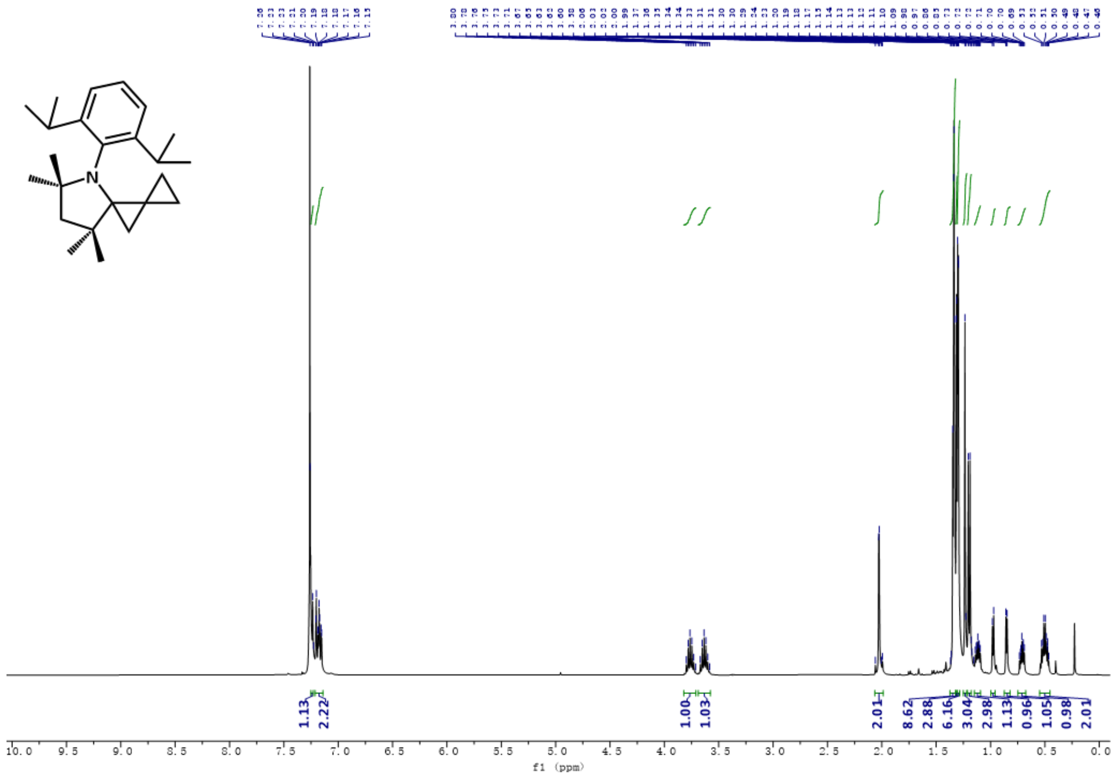

^{13}C{^{1}H} NMR (101 MHz, 298 K, C_6D_6) of **4**:

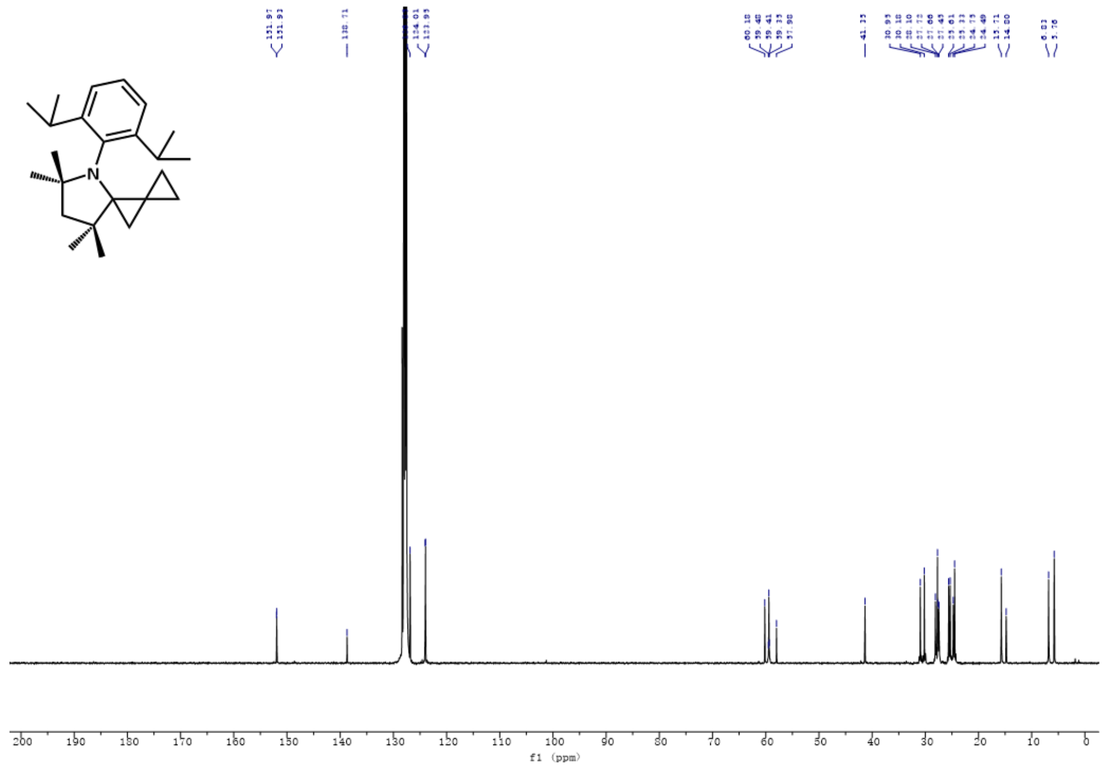

^{1}H NMR (400 MHz, 298 K, C_6D_6) of **5b**:

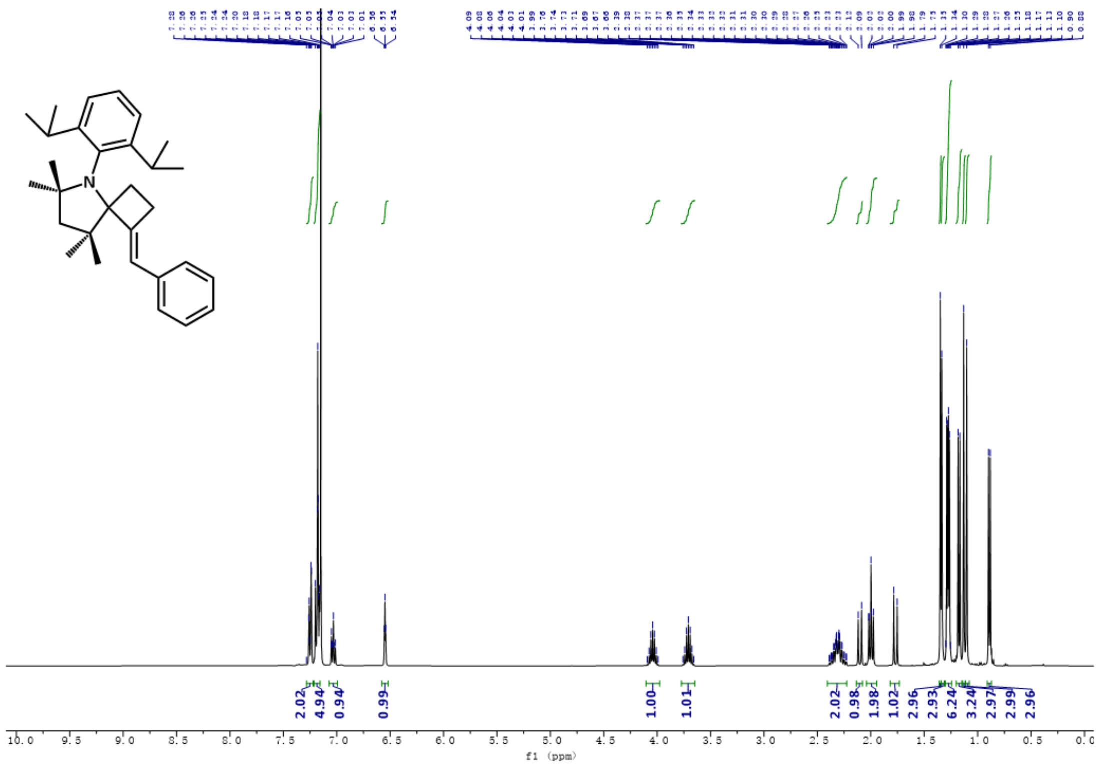

^{13}C{^{1}H} NMR (101 MHz, 298 K, C_6D_6) of **5b**:

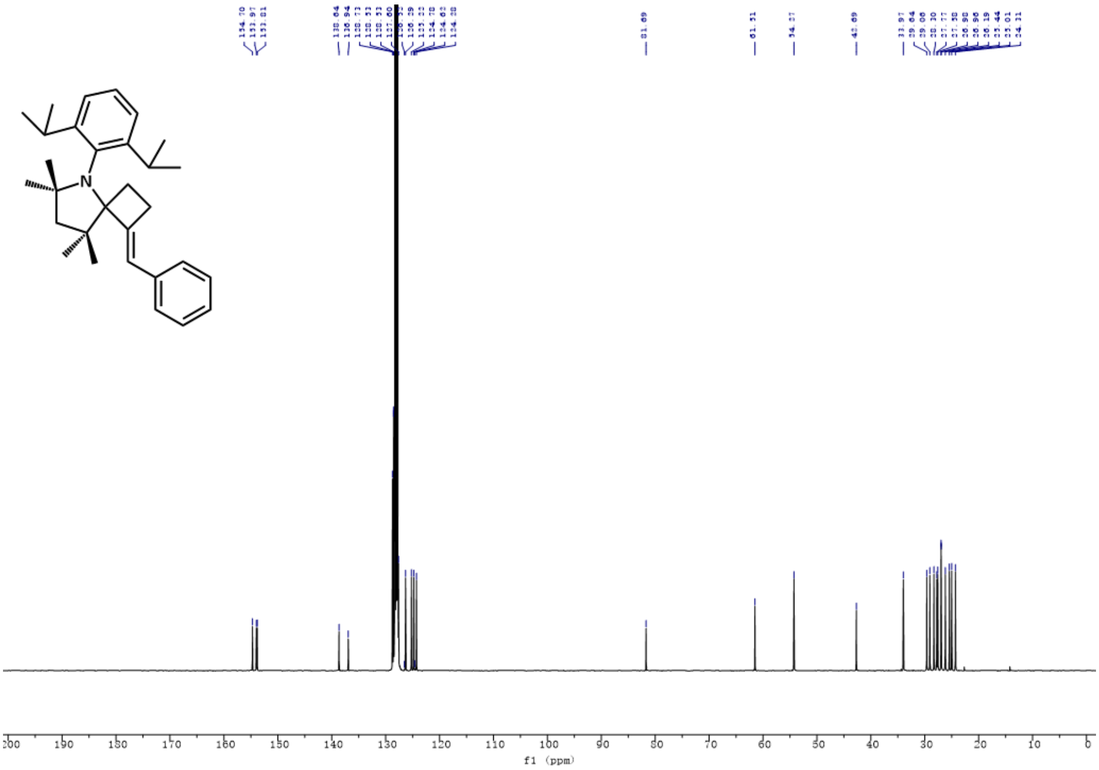

^{1}H NMR (400 MHz, 298 K, C_6D_6) of **5c**:

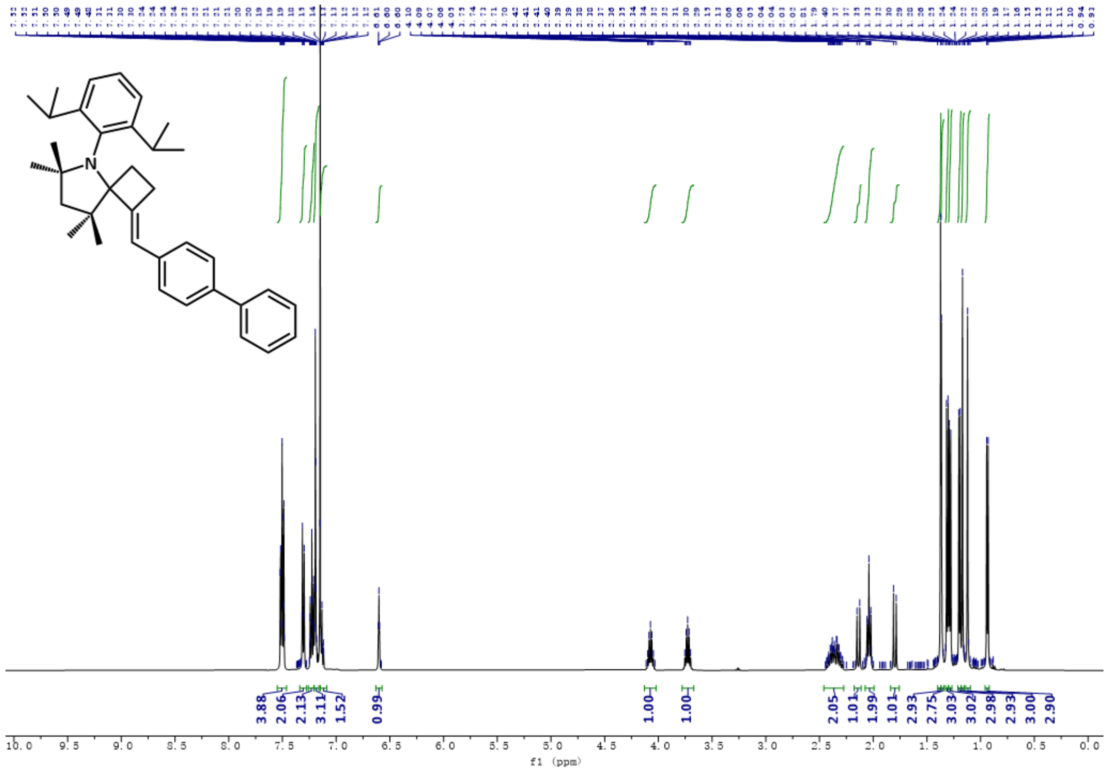

$^{13}C\{^{1}H\}$ NMR (101 MHz, 298 K, C_6D_6) of **5c**:

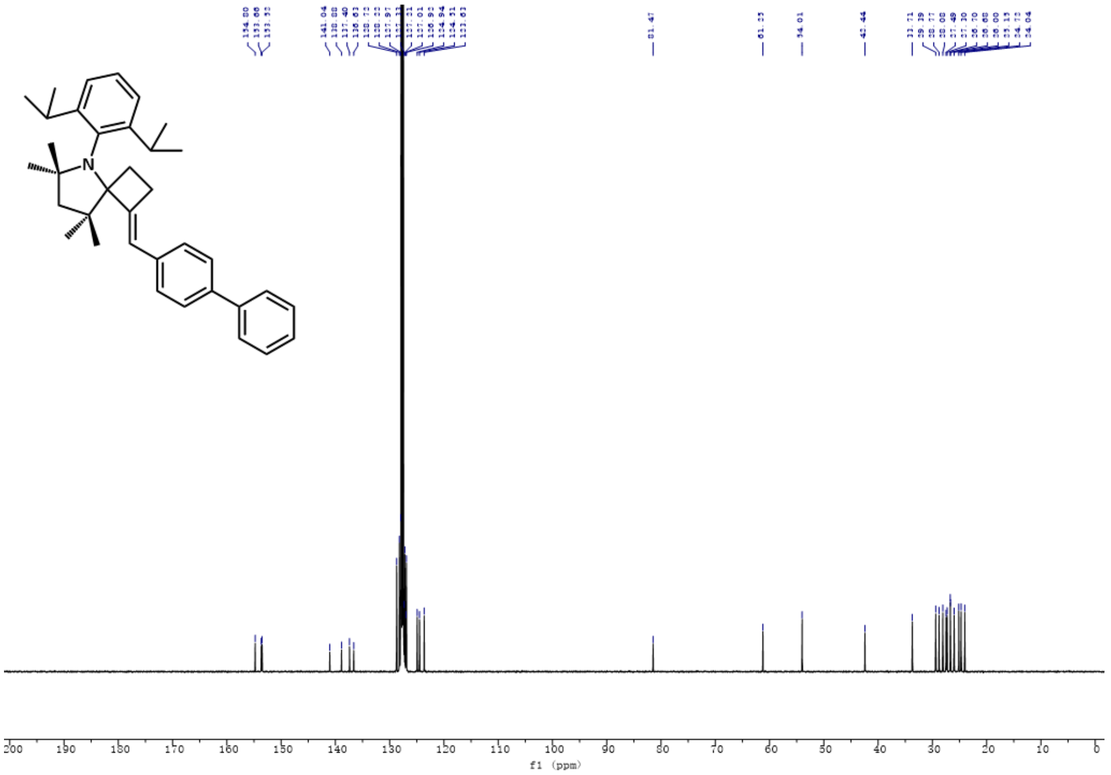

^{1}H NMR (400 MHz, 298 K, C_6D_6) of **5d**:

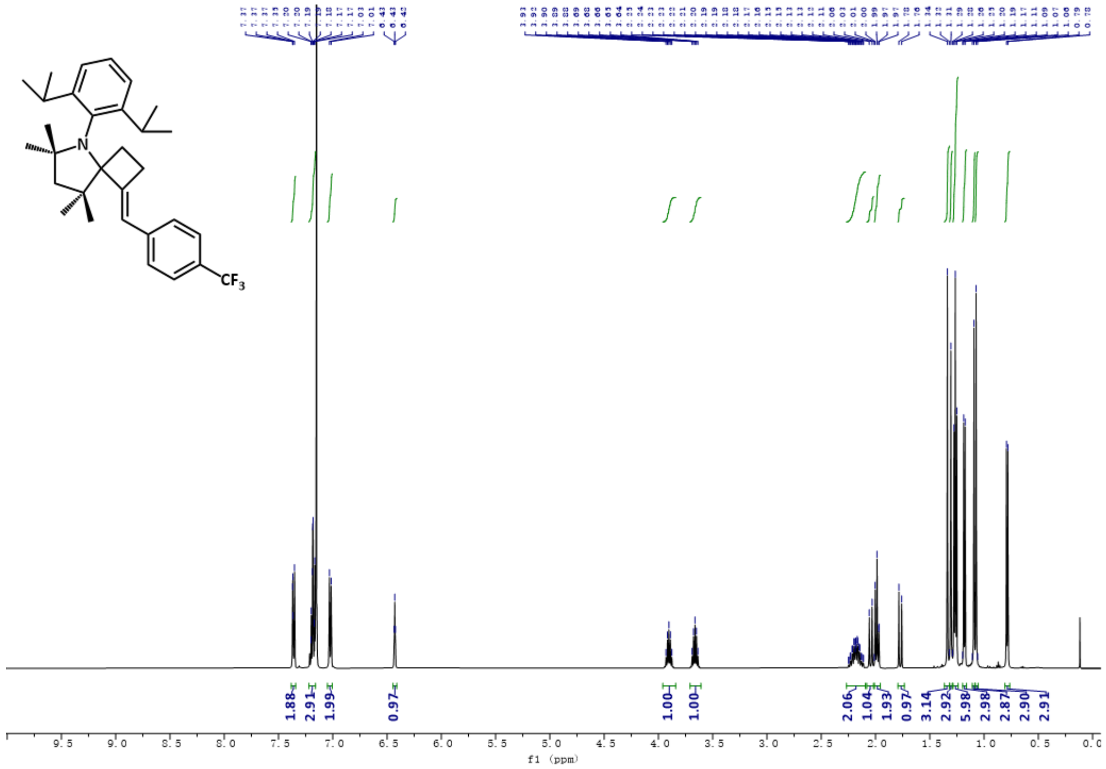

^{19}F NMR (376 MHz, 298 K, C_6D_6) of **5d**:

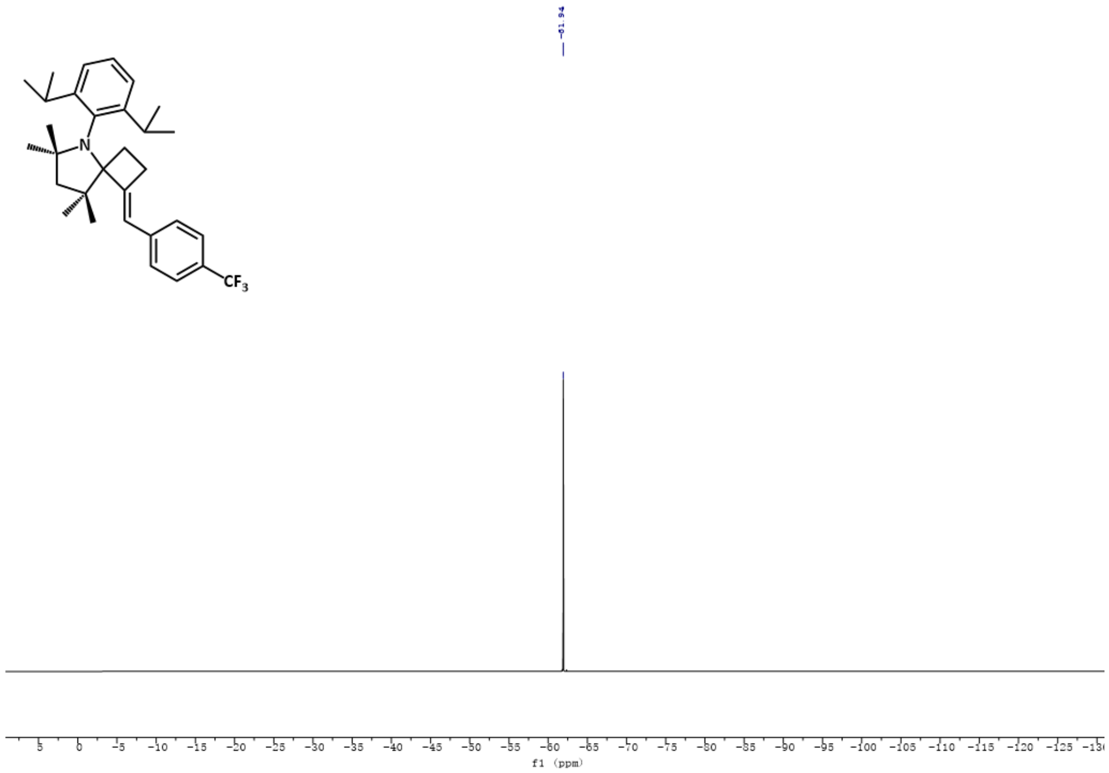

$^{13}C\{^1H\}$ NMR (101 MHz, 298 K, C_6D_6) of **5d**:

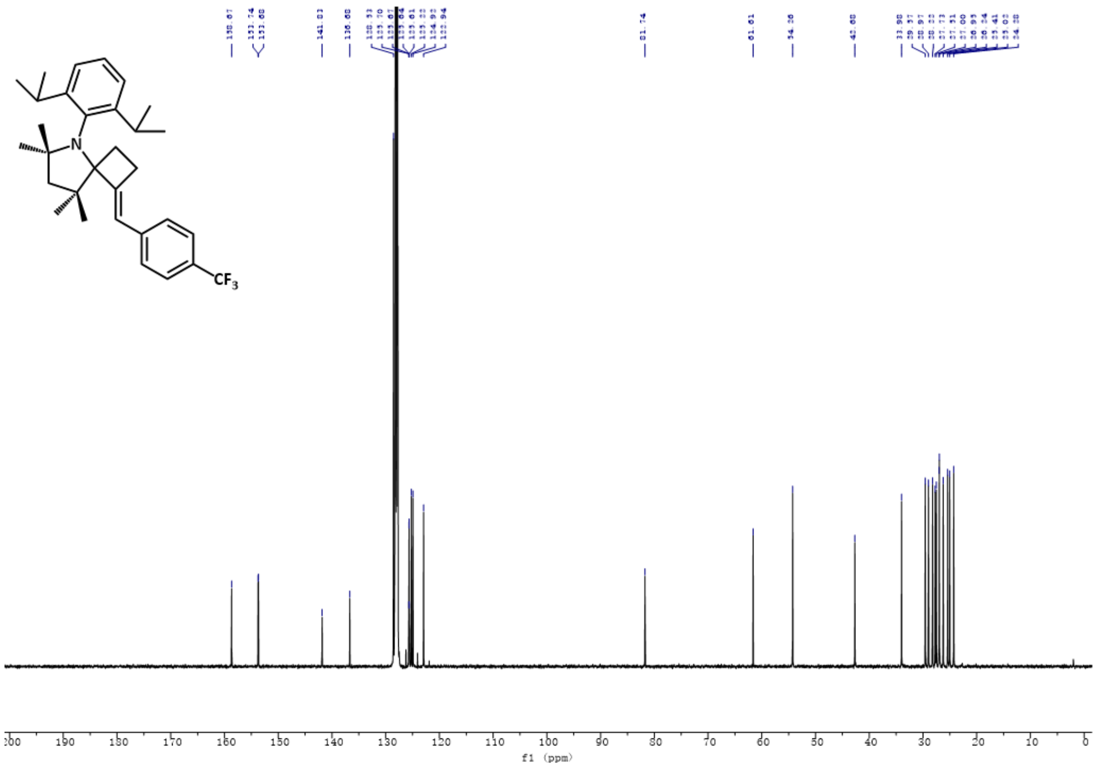

^1H NMR (400 MHz, 298 K, C_6D_6) of **5e**:

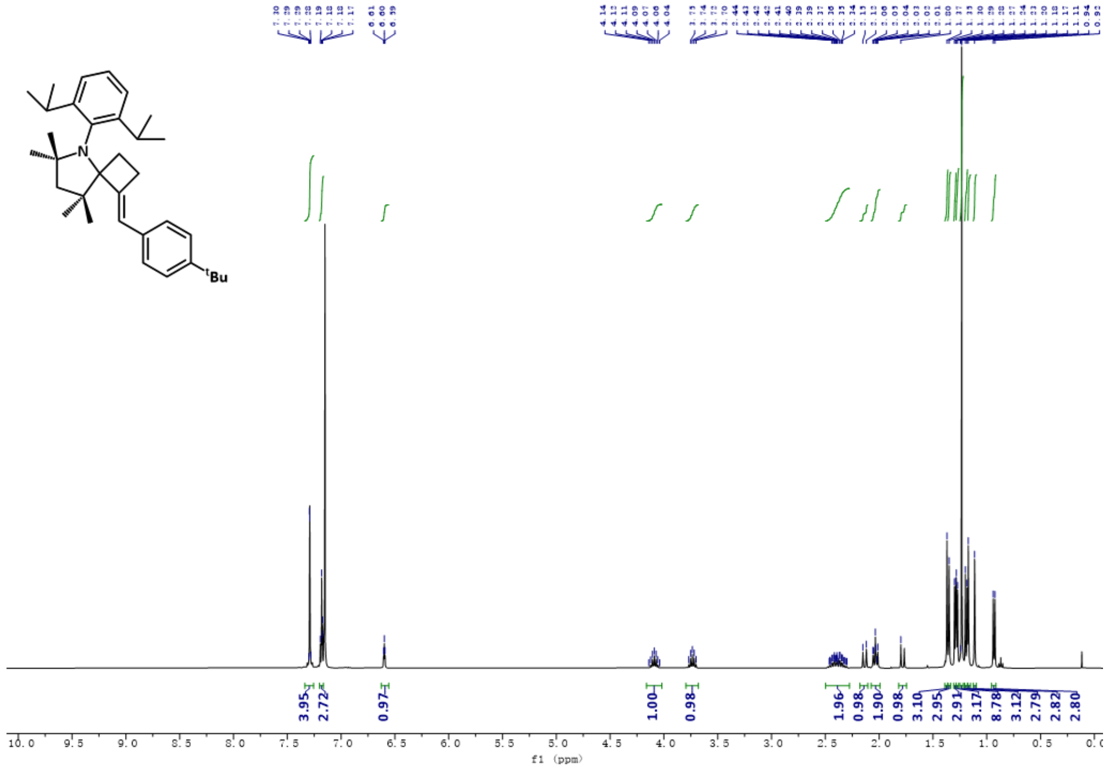

^{13}C{^{1}H} NMR (101 MHz, 298 K, C_6D_6) of **5e**:

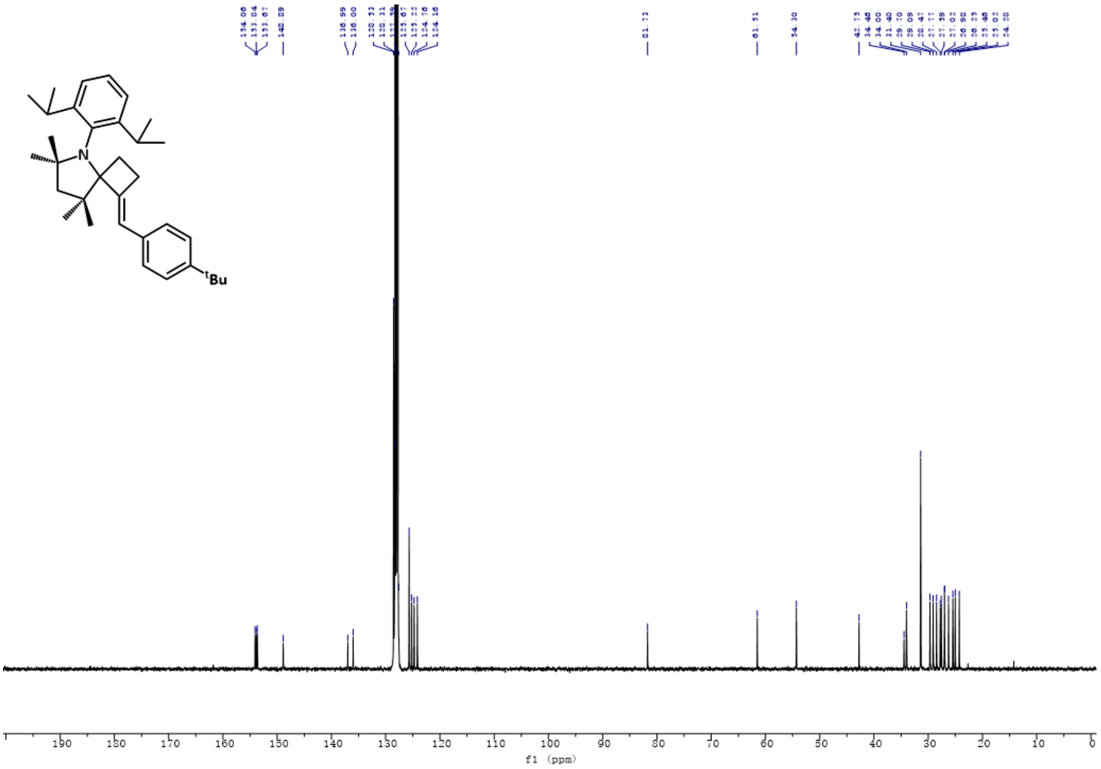

^{1}H NMR (400 MHz, 298 K, C_6D_6) of **5f**:

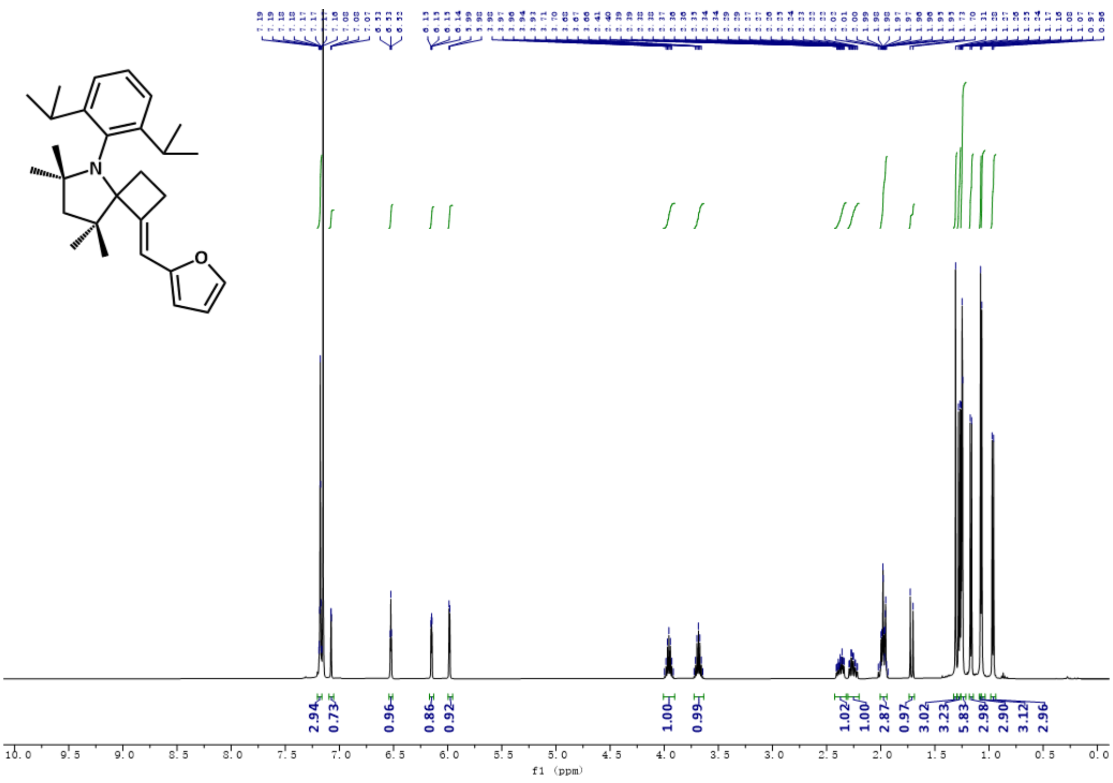

$^{13}C\{^{1}H\}$ **NMR** (101 MHz, 298 K, C_6D_6) of **5f**:

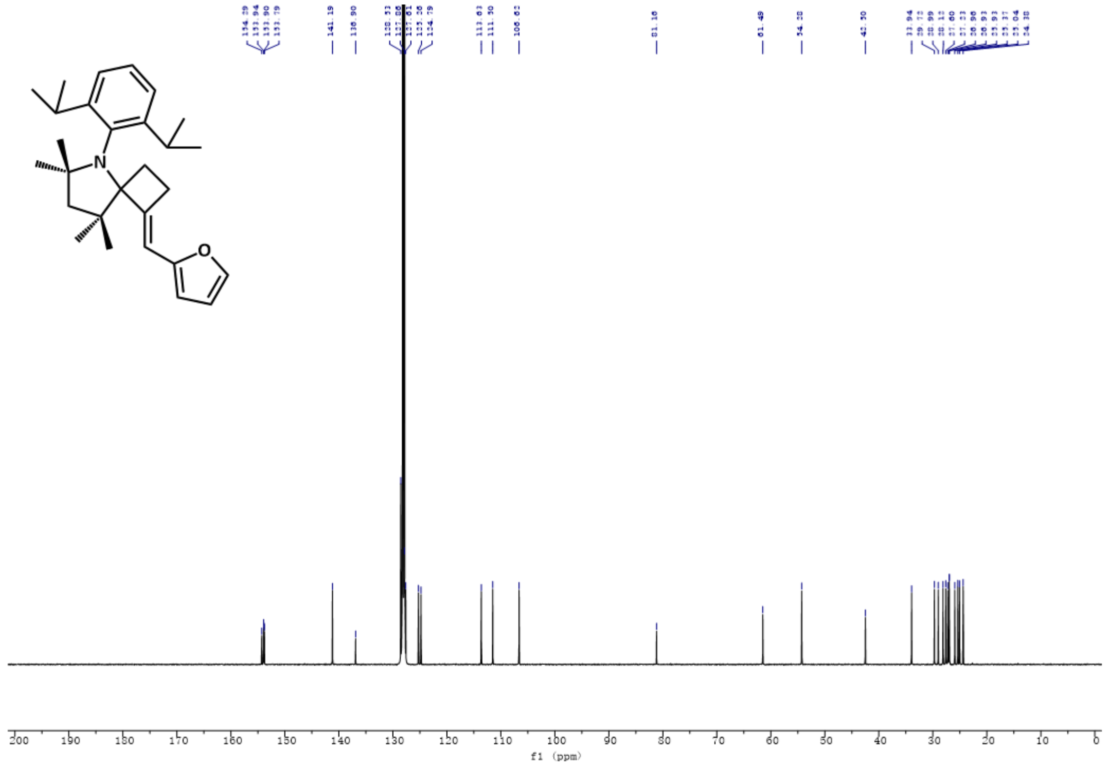

^{1}H **NMR** (400 MHz, 298 K, C_6D_6) of **S1**:

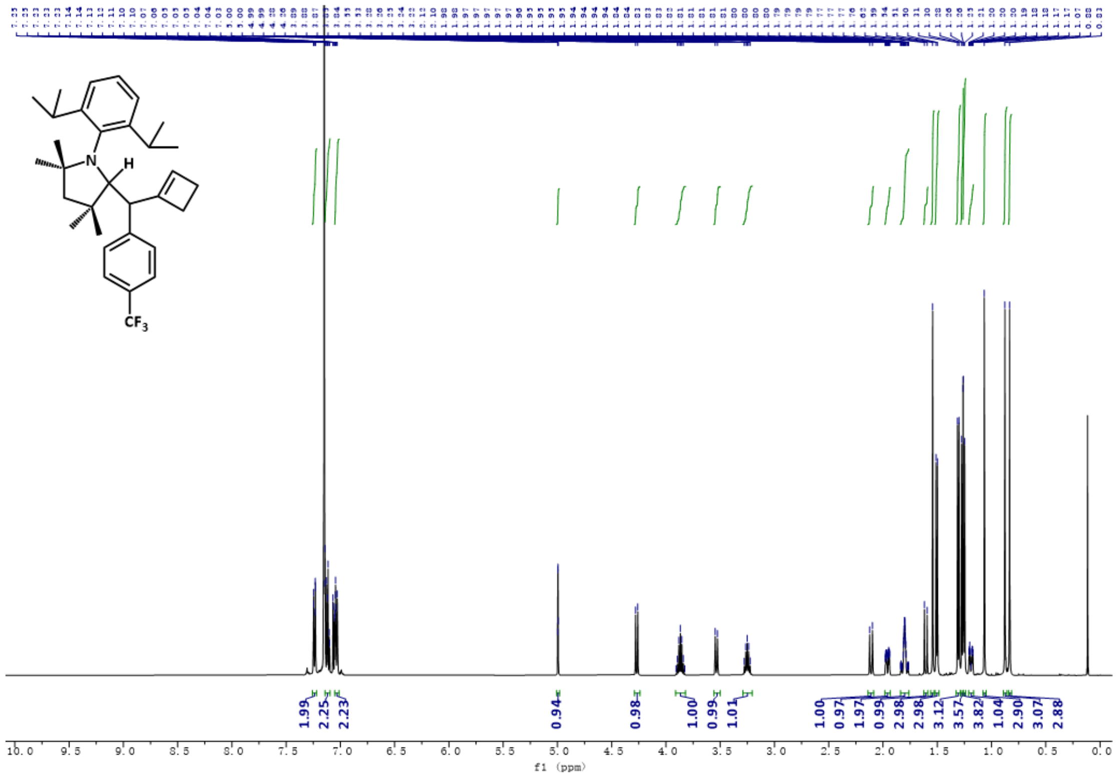

^{19}F NMR (376 MHz, 298 K, C_6D_6) of **S1**:

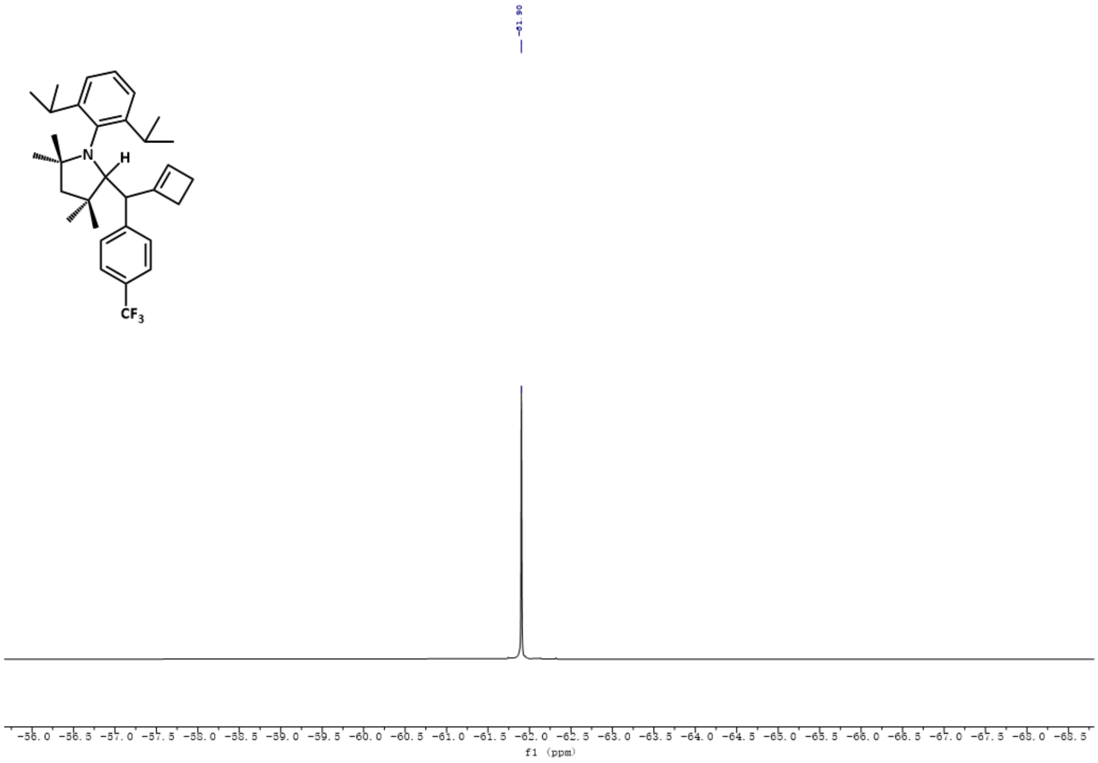

^{13}C{^{1}H} NMR (101 MHz, 298 K, C_6D_6) of **S1**:

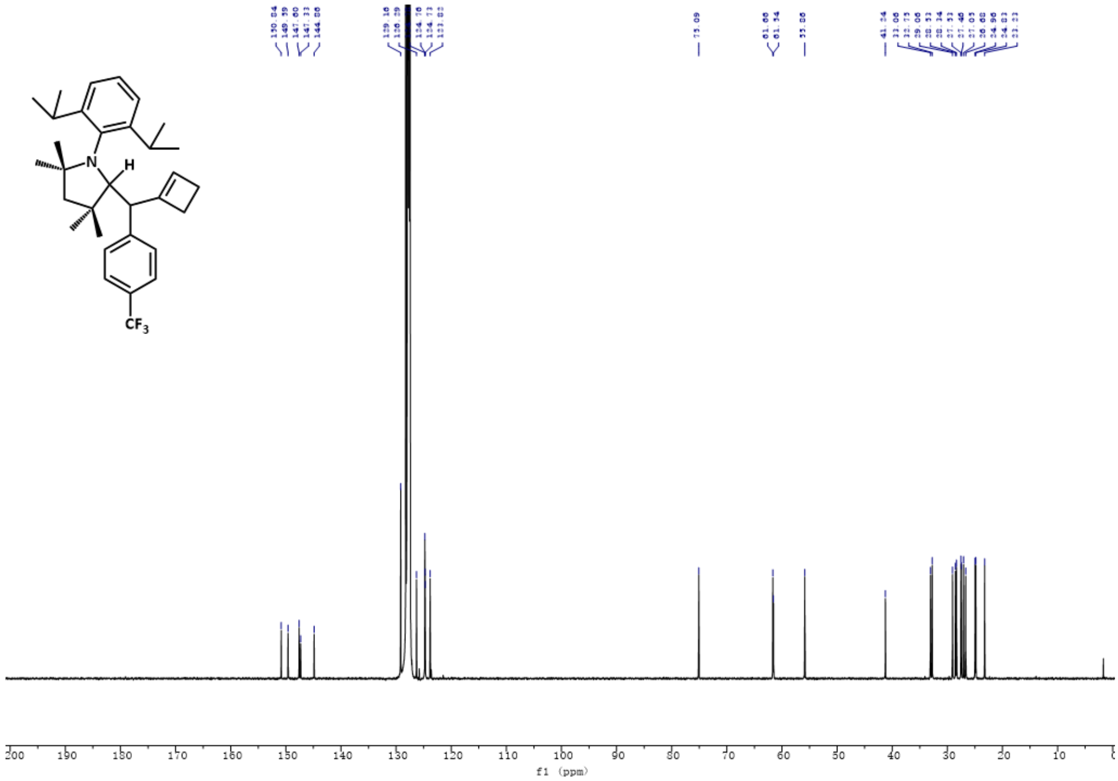

^{1}H NMR (400 MHz, 298 K, C_6D_6) of **S3**:

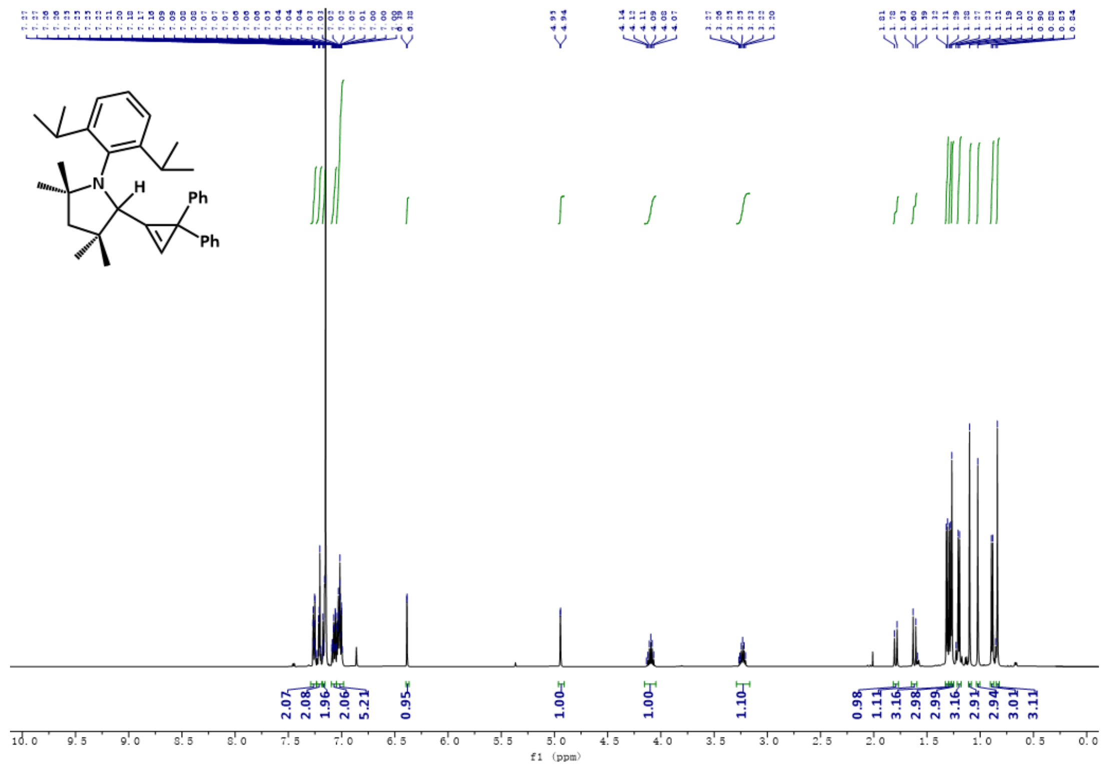

^{13}C{^{1}H} NMR (101 MHz, 298 K, C_6D_6) of **S3**:

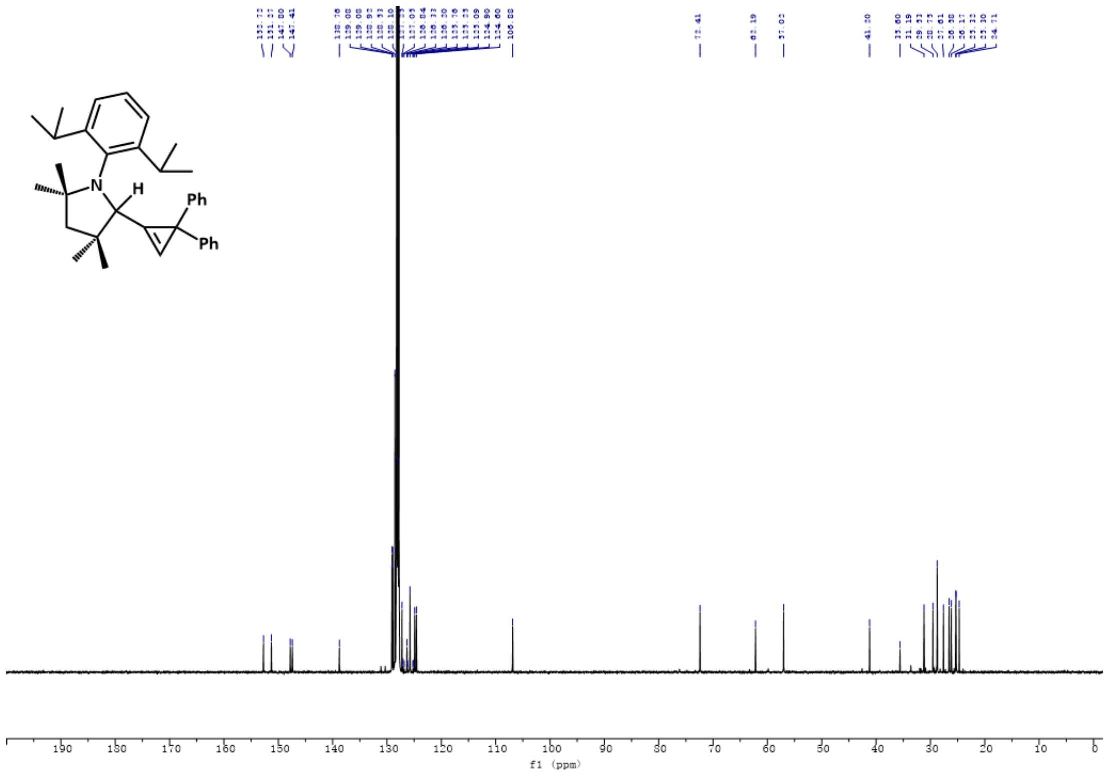

9) XYZ Coordinates

2

SCF (BP86-D3) = -1683.444660

E(SCF)+ZPE(0 K)= -1682.752296

H(298 K)= -1682.711993

G(298 K)= -1682.820040

Lowest Frequency = 31.7520 cm-1

N	-1.618340	-0.980765	-1.017951
N	1.072192	-1.498271	-0.606460
C	2.329591	-1.324754	0.079892
C	-2.904028	-0.968444	-0.355123
C	3.310787	-0.450782	-0.468341
C	-1.447348	-1.768602	-2.176991
C	-3.242337	-2.063281	0.490131
C	-0.487531	0.215952	1.550054
C	0.089295	1.242611	0.513297
C	-3.782999	0.131685	-0.530242
C	2.547402	-2.018146	1.306402
C	-3.402458	1.344465	-1.374716
H	-2.391957	1.148825	-1.790949
C	3.091745	0.316417	-1.772693
H	2.055358	0.114407	-2.112488
C	-0.278516	0.125445	3.037460
H	-0.112592	-0.871890	3.477123
H	0.298668	0.936567	3.514019
C	-2.671555	-1.950857	-3.036793
H	-3.504601	-2.411346	-2.465362
H	-2.451728	-2.590297	-3.912019
H	-3.045370	-0.968995	-3.396729
C	3.782381	-1.831681	1.960542
H	3.982544	-2.354655	2.907143
C	-1.648673	0.460029	2.489376
H	-2.454311	-0.292302	2.532016
H	-1.995155	1.503552	2.587223
C	-2.273501	-3.219502	0.737060
H	-1.275835	-2.916913	0.355028
C	4.530926	-0.305524	0.224886
H	5.307700	0.360824	-0.183509
C	-5.032221	0.097746	0.125651
H	-5.730559	0.940973	0.001683
C	1.481894	-2.952929	1.881506
H	0.500562	-2.462067	1.706428
C	4.769225	-0.989960	1.423886
H	5.728832	-0.863169	1.950222
C	-4.505549	-2.055995	1.114882
H	-4.794202	-2.898117	1.763564
C	-5.398501	-0.988566	0.931813
H	-6.381171	-1.000307	1.429686
C	4.047812	-0.174957	-2.880483
H	5.108447	0.016199	-2.609581
H	3.843420	0.356493	-3.833989
H	3.922498	-1.261459	-3.058098
C	-2.106954	-3.522232	2.238227
H	-1.796611	-2.617124	2.799090
H	-1.330500	-4.299425	2.389240
H	-3.044740	-3.902202	2.694844
C	3.226683	1.840304	-1.573356
H	2.535873	2.212096	-0.790499
H	2.992158	2.375201	-2.517701
H	4.259306	2.124792	-1.279198
C	-3.317073	2.614444	-0.502466
H	-4.316810	2.905895	-0.115611
H	-2.920731	3.469746	-1.088966
H	-2.655579	2.456380	0.372706
C	1.460246	-4.303400	1.133689
H	1.282657	-4.171270	0.049080
H	0.662907	-4.961427	1.539198
H	2.430963	-4.829565	1.257813
C	-2.686137	-4.478169	-0.052743
H	-3.687266	-4.838791	0.267028
H	-1.955583	-5.297884	0.111595
H	-2.725750	-4.272659	-1.141427
C	1.609717	-3.181714	3.397008
H	2.489850	-3.811281	3.648422
H	0.711951	-3.712556	3.775124
H	1.703931	-2.228088	3.955981
C	-4.370315	1.554854	-2.557032
H	-4.438508	0.650583	-3.195884
H	-4.032877	2.400168	-3.192804
H	-5.396356	1.791459	-2.203360
H	1.150090	1.507481	0.685567
H	-0.534559	2.122068	0.271300
Si	-0.231689	-0.472334	-0.100668
C	2.086551	-3.051233	-2.187299

H	3.031667	-3.070647	-1.628371
C	-0.241862	-2.323064	-2.520428
H	-0.208639	-2.883735	-3.465455
H	2.003412	-3.666538	-3.093537
C	1.016351	-2.302924	-1.773624

3

SCF (BP86-D3) = -1683.484792

E(SCF)+ZPE(0 K)= -1682.792832

H(298 K)= -1682.752244

G(298 K)= -1682.862104

Lowest Frequency = 16.5557 cm-1

N	-1.694058	-1.087570	-0.837124
N	1.005690	-1.492648	-0.506499
C	2.242233	-1.398459	0.232241
C	-2.982526	-1.112943	-0.187253
C	3.261302	-0.500567	-0.198322
C	-1.516640	-1.793902	-2.045173
C	-3.336467	-2.270115	0.564386
C	0.024580	1.411694	-0.046136
C	0.015382	2.448815	-0.902640
C	-3.870124	-0.009184	-0.303453
C	2.415800	-2.213353	1.390182
C	-3.494150	1.257182	-1.066558
H	-2.500691	1.081985	-1.527408
C	3.107582	0.396318	-1.425805
H	2.055203	0.320245	-1.766309
C	0.163251	1.404524	1.485763
H	1.240675	1.445119	1.766429
H	-0.343649	2.253808	1.995032
C	-2.741123	-1.947000	-2.911786
H	-3.560998	-2.465344	-2.372239
H	-2.510782	-2.517960	-3.830718
H	-3.139920	-0.951080	-3.200178
C	3.634657	-2.123042	2.092610
H	3.793493	-2.744994	2.986234
C	-0.431966	-0.013702	1.816683
H	0.117932	-0.601714	2.574266
H	-1.497915	0.047973	2.119148
C	-2.375598	-3.447262	0.724778
H	-1.380098	-3.122771	0.356577
C	4.464578	-0.455432	0.537986
H	5.267754	0.226188	0.215452
C	-5.129514	-0.096047	0.327739
H	-5.831156	0.749935	0.249562
C	1.334620	-3.194259	1.842733
H	0.358911	-2.772692	1.526101
C	4.655509	-1.258770	1.669212
H	5.603066	-1.208903	2.229256
C	-4.612196	-2.315493	1.161939
H	-4.910602	-3.207826	1.734984
C	-5.505767	-1.240061	1.044751
H	-6.497940	-1.292410	1.520807
C	4.011262	-0.073413	-2.585973
H	5.084533	-0.024499	-2.301422
H	3.868465	0.575363	-3.476210
H	3.773462	-1.115917	-2.876406
C	-2.207648	-3.850926	2.201386
H	-1.878186	-2.989136	2.817512
H	-1.446416	-4.652057	2.295660
H	-3.151214	-4.241912	2.636709
C	3.382351	1.875598	-1.086457
H	2.781884	2.209481	-0.217342
H	3.118480	2.523859	-1.948152
H	4.454830	2.050681	-0.855394
C	-3.352730	2.451655	-0.100662
H	-4.328721	2.711445	0.362514
H	-2.976387	3.347588	-0.637383
H	-2.636039	2.224845	0.713686
C	1.487473	-4.550731	1.121712
H	1.460863	-4.423940	0.021107
H	0.670375	-5.244234	1.413755
H	2.454373	-5.028221	1.390281
C	-2.803723	-4.648833	-0.142251
H	-3.805102	-5.023376	0.160521
H	-2.078602	-5.482985	-0.036666
H	-2.850073	-4.374835	-1.215327
C	1.282911	-3.380929	3.368941
H	2.175676	-3.918118	3.753331
H	0.397353	-3.985066	3.651165
H	1.217951	-2.408457	3.900431
C	-4.491083	1.574400	-2.199074
H	-4.601398	0.718912	-2.897015
H	-4.147151	2.452977	-2.784344
H	-5.500427	1.814171	-1.802228
H	-0.121146	2.293864	-1.986474

H	0.141204	3.494605	-0.565637
Si	-0.296031	-0.436577	-0.014755
C	2.039452	-3.001066	-2.122655
H	2.988878	-3.019700	-1.571007
C	-0.296643	-2.285199	-2.433685
H	-0.253017	-2.783885	-3.412540
H	1.954093	-3.608829	-3.033921
C	0.963549	-2.270021	-1.689039

3'

SCF (BP86-D3) = -1683.485969
E(SCF)+ZPE(0 K)= -1682.794387
H(298 K)= -1682.753941
G(298 K)= -1682.863263
Lowest Frequency = 17.8488 cm-1

N	-1.727798	-1.014089	-0.853911
N	0.981455	-1.433280	-0.490878
C	2.224934	-1.346781	0.240144
C	-3.017579	-1.067418	-0.203670
C	3.247337	-0.462609	-0.209091
C	-1.537205	-1.736317	-2.052800
C	-3.342098	-2.226514	0.559494
C	-3.936681	0.009215	-0.332757
C	2.407496	-2.158530	1.397673
C	-3.608562	1.272478	-1.123689
H	-2.590311	1.139471	-1.545592
C	3.091515	0.417413	-1.448187
H	2.034722	0.346080	-1.777771
C	-2.748654	-1.890028	-2.936938
H	-3.593511	-2.363900	-2.395005
H	-2.516316	-2.502548	-3.828124
H	-3.111978	-0.895981	-3.274040
C	3.629754	-2.061944	2.093803
H	3.793572	-2.679566	2.989655
C	-0.505447	-0.087753	1.872574
H	-0.124784	-0.750949	2.669614
H	-1.575935	0.145671	2.074965
C	-2.356787	-3.383198	0.719459
H	-1.362050	-3.029952	0.375907
C	4.453792	-0.411183	0.519530
H	5.257293	0.264159	0.185128
C	-5.193499	-0.105091	0.299637
H	-5.919745	0.718633	0.209660
C	1.340913	-3.150928	1.858856
H	0.361811	-2.765357	1.511334
C	4.647606	-1.199936	1.660721
H	5.596687	-1.143403	2.217466
C	-4.614978	-2.298581	1.160016
H	-4.889315	-3.192462	1.742280
C	-5.537339	-1.249152	1.031977
H	-6.527168	-1.322552	1.509977
C	3.972823	-0.082691	-2.613074
H	5.049409	-0.048954	-2.339282
H	3.831839	0.557226	-3.509988
H	3.713569	-1.124512	-2.886477
C	-2.203880	-3.814892	2.189276
H	-1.909698	-2.958835	2.830518
H	-1.421929	-4.596272	2.278494
H	-3.142703	-4.243204	2.598796
C	3.397765	1.897727	-1.136435
H	2.858374	2.244713	-0.231934
H	3.104238	2.544769	-1.989432
H	4.481739	2.060997	-0.958043
C	-3.591739	2.512010	-0.203633
H	-4.608738	2.741614	0.179308
H	-3.229824	3.406516	-0.752582
H	-2.936258	2.358754	0.678411
C	1.542131	-4.524427	1.183027
H	1.525125	-4.430952	0.078804
H	0.741084	-5.231859	1.486443
H	2.518455	-4.965874	1.477941
C	-2.740814	-4.576053	-0.180148
H	-3.738924	-4.978236	0.096798
H	-1.998500	-5.395202	-0.077209
H	-2.773019	-4.281009	-1.247953
C	1.266674	-3.292967	3.389409
H	2.161598	-3.803695	3.803508
H	0.386181	-3.902291	3.676521
H	1.180441	-2.306317	3.890893
C	-4.579600	1.484931	-2.303610
H	-4.605143	0.603049	-2.975604
H	-4.275455	2.367199	-2.905030
H	-5.616564	1.663941	-1.948344
Si	-0.337607	-0.409755	0.018267
C	2.002602	-2.984185	-2.074783
H	2.948755	-3.007339	-1.517969

C	-0.318181	-2.244278	-2.417792
H	-0.267589	-2.756623	-3.389237
H	1.917071	-3.597792	-2.982032
C	0.933552	-2.233363	-1.659654
C	-0.002661	1.456516	0.108965
H	-0.966492	1.995600	-0.010127
H	0.773309	1.986963	-0.469651
C	0.291932	1.187796	1.586036
C	1.216842	1.776525	2.370674
H	1.419959	1.407403	3.389264
H	1.809183	2.633688	2.010907

Int-S1

SCF (BP86-D3) = -1683.397868

E(SCF)+ZPE(0 K)= -1682.707458

H(298 K)= -1682.666349

G(298 K)= -1682.776480

Lowest Frequency = 27.0980 cm-1

N	2.913194	2.961136	4.401988
C	1.477733	4.906478	4.738142
H	1.202161	5.934223	4.463269
H	0.896437	4.403429	5.522448
N	4.588085	3.094493	2.266359
C	2.517501	4.287855	4.093066
C	3.304816	4.994988	3.083124
H	3.114758	6.075106	3.005590
C	4.224640	4.457364	2.220229
C	4.955571	5.308800	1.211147
H	4.576529	6.348214	1.215437
H	4.858400	4.903056	0.182843
H	6.043294	5.324297	1.438658
C	2.233334	2.289036	5.481073
C	2.636978	2.523438	6.824098
C	1.996323	1.793663	7.848107
H	2.303711	1.947757	8.895033
C	0.976372	0.877686	7.555402
H	0.487913	0.317478	8.368708
C	0.574430	0.677261	6.225412
H	-0.233613	-0.037827	6.007428
C	1.191250	1.372263	5.166278
C	0.728971	1.169265	3.725869
H	1.571873	1.475445	3.072065
C	-0.452169	2.106032	3.392048
H	-1.331878	1.869770	4.028805
H	-0.756602	1.988402	2.330221
H	-0.176654	3.166373	3.559633
C	0.384709	-0.294263	3.397657
H	1.201963	-0.983374	3.693992
H	0.215388	-0.410807	2.307235
H	-0.543719	-0.627242	3.908045
C	3.740246	3.518288	7.178684
H	4.048677	4.026389	6.242131
C	3.230421	4.611301	8.139129
H	2.351835	5.133017	7.707806
H	4.023431	5.366175	8.324275
H	2.934910	4.190368	9.123884
C	4.980972	2.795379	7.742407
H	4.744248	2.256279	8.684516
H	5.793247	3.520327	7.961481
H	5.372667	2.053891	7.014051
C	4.991243	2.456230	1.041973
C	6.358129	2.175115	0.787320
C	6.692660	1.527279	-0.422722
H	7.747883	1.290640	-0.634246
C	5.709598	1.195068	-1.363321
H	5.991539	0.699014	-2.305764
C	4.361749	1.496567	-1.103154
H	3.595763	1.230730	-1.848324
C	3.972615	2.115365	0.099006
C	2.495086	2.391715	0.383925
H	2.395060	2.521285	1.482522
C	1.575389	1.228081	-0.035595
H	1.490382	1.144082	-1.139648
H	0.550996	1.393499	0.357549
H	1.937507	0.253709	0.348527
C	2.016826	3.702003	-0.278692
H	2.582272	4.580099	0.086171
H	0.943697	3.878741	-0.054922
H	2.133374	3.647355	-1.382404
C	7.459701	2.550460	1.774915
H	6.984641	3.136989	2.590042
C	8.081711	1.291333	2.411811
H	8.598702	0.670312	1.649214
H	7.303851	0.661593	2.887858
H	8.827159	1.566585	3.187563
C	8.537115	3.443523	1.127659

H	9.097462	2.902055	0.336090
H	9.275234	3.775849	1.887571
H	8.089605	4.346647	0.663523
C	3.917055	-0.101149	2.444819
H	4.586885	-0.255801	1.584172
H	2.840035	-0.031863	2.237325
C	4.432577	0.250060	3.706552
C	4.105198	-0.376409	5.055174
C	5.509101	-0.474853	4.481930
H	3.447951	-1.259639	5.021391
H	3.950832	0.271703	5.932490
H	6.296615	0.114106	4.979205
H	5.838541	-1.439335	4.061780
Si	4.449082	2.264895	3.834710

TS-1

SCF (BP86-D3) = -1683.395554

E(SCF)+ZPE(0 K)= -1682.705443

H(298 K)= -1682.664938

G(298 K)= -1682.773915

Lowest Frequency = -234.7249 cm-1

N	2.831341	2.900849	4.345910
C	1.518143	4.911651	4.757845
H	1.308867	5.966235	4.531638
H	0.896947	4.406014	5.509355
N	4.653556	3.077094	2.273081
C	2.528208	4.262246	4.097604
C	3.364590	4.967707	3.124641
H	3.196583	6.052774	3.068930
C	4.294471	4.442339	2.262683
C	5.024680	5.310313	1.267913
H	4.660375	6.354056	1.307760
H	4.900328	4.935230	0.230681
H	6.116321	5.303652	1.474241
C	2.166881	2.220191	5.428384
C	2.605752	2.432086	6.765265
C	1.990057	1.684616	7.791086
H	2.322705	1.819902	8.832934
C	0.962383	0.774001	7.507247
H	0.496075	0.197655	8.322284
C	0.520522	0.603154	6.186017
H	-0.298481	-0.102098	5.978305
C	1.107749	1.320221	5.123616
C	0.587170	1.174930	3.695227
H	1.428646	1.429905	3.018795
C	-0.533162	2.199442	3.413143
H	-1.407030	2.018970	4.075694
H	-0.878639	2.117760	2.360396
H	-0.177643	3.235425	3.581732
C	0.124576	-0.252622	3.354407
H	0.890025	-1.010148	3.623512
H	-0.075470	-0.338846	2.266710
H	-0.815479	-0.521848	3.881041
C	3.703654	3.436861	7.116343
H	4.001742	3.954045	6.181471
C	3.178756	4.520067	8.081020
H	2.289898	5.027334	7.654082
H	3.959420	5.288246	8.263845
H	2.894863	4.091204	9.065734
C	4.961120	2.742350	7.678179
H	4.734052	2.168722	8.601899
H	5.741825	3.491210	7.928687
H	5.395592	2.039533	6.936523
C	5.030910	2.459562	1.027239
C	6.396662	2.213569	0.736513
C	6.717156	1.619029	-0.504486
H	7.771897	1.414018	-0.748626
C	5.718679	1.295578	-1.431917
H	5.988991	0.841247	-2.398506
C	4.370027	1.547834	-1.126524
H	3.591063	1.285897	-1.859837
C	3.997678	2.119850	0.104121
C	2.525511	2.361981	0.435653
H	2.456735	2.506250	1.534116
C	1.629167	1.161956	0.077035
H	1.544878	1.017112	-1.020788
H	0.602077	1.327757	0.463407
H	2.013831	0.221040	0.518179
C	2.000419	3.651211	-0.230962
H	2.577743	4.540213	0.089466
H	0.938077	3.823814	0.042127
H	2.063793	3.577172	-1.337926
C	7.507521	2.560771	1.723767
H	7.036556	3.108874	2.567281
C	8.145653	1.279689	2.298496
H	8.659152	0.697511	1.503634

H	7.377693	0.623480	2.753951
H	8.897436	1.526175	3.077887
C	8.572372	3.485772	1.101424
H	9.128039	2.978611	0.284281
H	9.316158	3.793251	1.866222
H	8.115757	4.403327	0.676252
C	3.871973	0.026931	2.736068
H	4.389059	-0.033283	1.766823
H	2.786336	-0.135403	2.762658
C	4.583991	0.335144	3.939436
C	4.413335	-0.328931	5.295221
C	5.759602	-0.353130	4.602288
H	3.795427	-1.241133	5.284358
H	4.289633	0.296458	6.191850
H	6.565670	0.267986	5.023895
H	6.087461	-1.292757	4.127798
Si	4.347091	2.207987	3.774035

TS-2

SCF (BP86-D3) = -1683.400185

E(SCF)+ZPE(0 K)= -1682.709628

H(298 K)= -1682.669759

G(298 K)= -1682.776922

Lowest Frequency = -252.7800 cm-1

N	-1.656649	-0.909921	-0.859315
N	1.036102	-1.370497	-0.547714
C	2.316794	-1.336097	0.122003
C	-2.950371	-1.005727	-0.226156
C	3.374051	-0.561074	-0.430250
C	-1.493065	-1.601522	-2.080769
C	-3.271995	-2.203213	0.476939
C	0.468928	0.995299	1.178604
C	0.023929	1.626750	-0.107862
C	-3.884099	0.063071	-0.316234
C	2.515477	-2.121924	1.288473
C	-3.557602	1.370376	-1.032585
H	-2.535991	1.266307	-1.453264
C	3.195463	0.278379	-1.693068
H	2.122967	0.233739	-1.973645
C	-0.126635	1.152973	2.497387
H	0.459527	0.826255	3.376376
H	-0.725686	2.065352	2.697068
C	-2.700072	-1.668808	-2.984108
H	-3.567439	-2.139887	-2.476316
H	-2.477156	-2.245747	-3.901312
H	-3.023532	-0.647634	-3.276785
C	3.786757	-2.109823	1.899442
H	3.958112	-2.716558	2.803005
C	-0.943267	0.013574	1.815224
H	-0.805237	-0.975790	2.287230
H	-2.000072	0.309958	1.673425
C	-2.271615	-3.351042	0.600742
H	-1.274783	-2.957248	0.312089
C	4.627326	-0.582395	0.214799
H	5.456892	0.013372	-0.198936
C	-5.147824	-0.097124	0.291355
H	-5.883579	0.720808	0.229438
C	1.411625	-3.003008	1.865811
H	0.469585	-2.735505	1.345457
C	4.838049	-1.347972	1.370796
H	5.825444	-1.352489	1.859613
C	-4.551776	-2.320378	1.055684
H	-4.821057	-3.243953	1.592100
C	-5.486913	-1.278718	0.964204
H	-6.482101	-1.387317	1.424068
C	4.001541	-0.302456	-2.874077
H	5.092249	-0.283540	-2.661479
H	3.827298	0.290763	-3.796917
H	3.703548	-1.351519	-3.072989
C	-2.160156	-3.874866	2.044557
H	-1.952620	-3.052233	2.759602
H	-1.336515	-4.613265	2.122657
H	-3.089435	-4.383429	2.376897
C	3.560147	1.757378	-1.448048
H	3.035755	2.163465	-0.558650
H	3.287649	2.378264	-2.327377
H	4.650244	1.885770	-1.277711
C	-3.549121	2.554404	-0.042392
H	-4.564743	2.745373	0.364923
H	-3.206557	3.484699	-0.542460
H	-2.877209	2.364092	0.819419
C	1.684614	-4.489751	1.555737
H	1.765297	-4.651399	0.461679
H	0.866852	-5.133519	1.943763
H	2.632747	-4.826282	2.027511
C	-2.602066	-4.493119	-0.382376

H	-3.601214	-4.927560	-0.163763
H	-1.848198	-5.304303	-0.303638
H	-2.602640	-4.133162	-1.430268
C	1.200514	-2.765647	3.372991
H	2.075595	-3.098792	3.969971
H	0.319117	-3.332299	3.737498
H	1.038168	-1.690208	3.594848
C	-4.525387	1.648763	-2.201179
H	-4.550893	0.804741	-2.920040
H	-4.217709	2.561630	-2.753096
H	-5.563111	1.811010	-1.839937
H	0.893927	1.914422	-0.725340
H	-0.804109	2.367651	-0.150043
Si	-0.213716	-0.249160	-0.066927
C	1.914018	-3.134620	-1.996020
H	2.849305	-3.213586	-1.426847
C	-0.305913	-2.177351	-2.443618
H	-0.271608	-2.681268	-3.420222
H	1.781364	-3.783818	-2.872833
C	0.920475	-2.255250	-1.652877

TS-2'

SCF (BP86-D3) = -1683.364725

E(SCF)+ZPE(0 K)= -1682.676872

H(298 K)= -1682.636195

G(298 K)= -1682.745389

Lowest Frequency = -545.3912 cm-1

N	-1.641334	-0.952654	-1.002879
N	1.050661	-1.448186	-0.536639
C	2.311052	-1.321548	0.158482
C	-2.934911	-0.954441	-0.351468
C	3.284625	-0.399457	-0.317777
C	-1.459629	-1.785138	-2.130487
C	-3.262938	-2.050039	0.496152
C	-0.567506	0.581202	1.468375
C	-0.082254	1.411976	0.210637
C	-3.843954	0.114892	-0.564207
C	2.561203	-2.154337	1.289212
C	-3.482742	1.338405	-1.400499
H	-2.451145	1.182858	-1.780258
C	3.047817	0.496478	-1.533458
H	1.973857	0.421967	-1.802923
C	0.421212	0.072412	2.403326
H	0.220514	-0.882001	2.906727
H	1.271166	0.678013	2.780080
C	-2.680457	-2.022702	-2.981095
H	-3.094790	-1.060453	-3.348786
H	-3.491544	-2.510173	-2.400730
H	-2.438879	-2.662920	-3.849954
C	3.818444	-2.055633	1.917233
H	4.040628	-2.687615	2.789813
C	-1.754744	0.699102	2.290732
H	-2.691098	0.151834	2.086958
H	-1.641880	1.146364	3.293473
C	-2.266845	-3.169838	0.792235
H	-1.280259	-2.868883	0.382036
C	4.526282	-0.340872	0.350845
H	5.297973	0.360129	-0.004879
C	-5.110252	0.045311	0.055367
H	-5.830961	0.864352	-0.097570
C	1.506823	-3.131125	1.808704
H	0.520423	-2.672758	1.590815
C	4.796703	-1.163216	1.451666
H	5.774496	-1.106384	1.956206
C	-4.542961	-2.077986	1.084350
H	-4.821409	-2.922188	1.734940
C	-5.465004	-1.043556	0.862503
H	-6.461066	-1.082344	1.331417
C	3.865982	0.015448	-2.751625
H	4.956056	0.065801	-2.541188
H	3.661519	0.655891	-3.635704
H	3.608768	-1.030103	-3.012781
C	-2.081268	-3.373672	2.308022
H	-1.776173	-2.426658	2.800493
H	-1.298911	-4.135759	2.500734
H	-3.012867	-3.728088	2.796870
C	3.350509	1.978160	-1.229612
H	2.822902	2.329506	-0.319506
H	3.034835	2.619453	-2.078823
H	4.436238	2.151025	-1.072516
C	-3.488286	2.610894	-0.525077
H	-4.521265	2.879719	-0.217172
H	-3.069694	3.475396	-1.082272
H	-2.895405	2.462330	0.402099
C	1.548760	-4.480482	1.060558
H	1.417369	-4.346025	-0.030469

H	0.744294	-5.151947	1.429635
H	2.522242	-4.989385	1.228673
C	-2.658299	-4.481131	0.082112
H	-3.644576	-4.850135	0.436449
H	-1.904080	-5.271072	0.281517
H	-2.718569	-4.339312	-1.015965
C	1.588010	-3.357364	3.328998
H	2.479102	-3.957430	3.610590
H	0.697849	-3.918767	3.679236
H	1.632944	-2.401122	3.889770
C	-4.409779	1.506493	-2.621540
H	-4.402809	0.605686	-3.268926
H	-4.090525	2.373614	-3.236974
H	-5.460741	1.686153	-2.310393
H	0.908383	1.865277	0.393261
H	-0.819314	2.120654	-0.198733
Si	-0.244909	-0.358334	-0.155584
C	2.094507	-2.998970	-2.108177
H	3.045059	-2.989716	-1.559237
C	-0.244730	-2.330326	-2.450243
H	-0.196516	-2.915984	-3.379253
H	2.015698	-3.632351	-3.002371
C	1.008385	-2.271346	-1.699615

TS-0

SCF (BP86-D3) = -990.855089

E(SCF)+ZPE(0 K)= -990.319304

H(298 K)= -990.290663

G(298 K)= -990.372660

Lowest Frequency = -205.4584 cm-1

N	0.251189	7.063165	15.209252
C	-0.258860	6.332819	14.233237
C	-0.979376	5.148426	14.881385
C	-0.475227	5.129622	16.356098
H	0.353816	4.397100	16.443962
H	-1.260979	4.826480	17.077675
C	0.059444	6.544596	16.659653
C	-0.944023	7.434663	17.413917
H	-0.596864	8.487582	17.441752
H	-1.036465	7.079456	18.460386
H	-1.952666	7.413194	16.961093
C	1.377237	6.518783	17.436138
H	2.144269	5.908826	16.921553
H	1.204214	6.075441	18.438541
H	1.773612	7.543017	17.584334
C	-2.496830	5.478266	14.793018
H	-2.757789	6.421591	15.311828
H	-3.086568	4.662617	15.262163
H	-2.813263	5.580060	13.735254
C	-0.757337	3.795249	14.185130
H	-1.001314	3.861189	13.105726
H	-1.407473	3.020475	14.645126
H	0.294969	3.466117	14.275935
C	0.735144	8.415940	14.981245
C	2.129398	8.697880	14.944153
C	2.532177	10.045227	14.811914
H	3.607589	10.282050	14.788958
C	1.600330	11.080887	14.680286
H	1.939994	12.123742	14.577276
C	0.232726	10.777135	14.650406
H	-0.498823	11.586998	14.504151
C	-0.228034	9.453198	14.797615
C	-1.726516	9.183314	14.673235
H	-1.914164	8.154768	15.025455
C	-2.587104	10.128020	15.532532
H	-2.256842	10.131086	16.591598
H	-3.649802	9.807989	15.505011
H	-2.553091	11.175093	15.164292
C	-2.156044	9.216611	13.191747
H	-1.959906	10.211710	12.738210
H	-3.241837	9.003004	13.095554
H	-1.601374	8.450873	12.613573
C	3.216286	7.622325	14.923573
H	2.716954	6.636223	15.016351
C	3.950497	7.640236	13.563614
H	3.241067	7.539353	12.721757
H	4.682292	6.806915	13.506218
H	4.514424	8.587120	13.426886
C	4.247055	7.777157	16.061872
H	4.847935	8.702207	15.932791
H	4.955785	6.922656	16.057915
H	3.774974	7.824764	17.061835
C	-0.090790	6.104238	11.324432
C	0.897656	7.231205	11.684763
H	0.031641	5.597367	10.350518
H	-1.131830	6.226978	11.663728

H	0.511337	8.093897	12.249934
H	1.684880	7.475182	10.949305
C	0.966954	5.902045	12.349423
C	1.895366	4.959415	12.676322
H	2.873633	5.231280	13.101845
H	1.662293	3.885054	12.603655

TS-3

SCF (BP86-D3) = -990.867846

E(SCF)+ZPE(0 K)= -990.331825

H(298 K)= -990.303532

G(298 K)= -990.384188

Lowest Frequency = -133.2225 cm-1

N	0.810223	7.154795	15.113020
C	1.783539	6.306469	14.633795
C	2.227527	5.313603	15.712138
C	1.429234	5.813376	16.949700
H	2.086820	6.456984	17.570908
H	1.092137	4.976534	17.593107
C	0.246749	6.665046	16.434615
C	-1.002736	5.780016	16.236926
H	-1.897668	6.367547	15.966604
H	-1.229088	5.246440	17.183101
H	-0.831826	5.018741	15.450845
C	-0.067941	7.840422	17.365300
H	0.823013	8.479919	17.521882
H	-0.396749	7.451646	18.350284
H	-0.877284	8.478336	16.958745
C	1.871183	3.867818	15.284912
H	0.780843	3.732141	15.145290
H	2.216836	3.141647	16.050067
H	2.378224	3.632881	14.325716
C	3.738880	5.348128	16.068670
H	4.346658	4.814424	15.314296
H	3.893931	4.846843	17.049179
H	4.121742	6.384939	16.149374
C	0.641978	8.509187	14.656530
C	1.723034	9.426037	14.835128
C	1.581179	10.738386	14.345272
H	2.410593	11.451244	14.474347
C	0.397447	11.165085	13.723184
H	0.303994	12.199779	13.357340
C	-0.665632	10.265540	13.578162
H	-1.594807	10.601607	13.090894
C	-0.568951	8.929778	14.021468
C	-1.752019	7.994674	13.777885
H	-1.417356	6.972110	14.041647
C	-2.949127	8.365415	14.679847
H	-2.664121	8.416350	15.750168
H	-3.770073	7.624490	14.576800
H	-3.355443	9.360882	14.401139
C	-2.199484	7.954304	12.300813
H	-2.598921	8.934102	11.964443
H	-3.009033	7.206253	12.167769
H	-1.365790	7.677156	11.625593
C	3.009821	9.046617	15.562917
H	2.826133	8.086893	16.082675
C	4.175216	8.823727	14.576949
H	3.966779	8.014076	13.840577
H	5.101790	8.551835	15.127205
H	4.385336	9.754192	14.006404
C	3.385628	10.070916	16.653771
H	3.697043	11.045221	16.221701
H	4.239750	9.691909	17.253298
H	2.538173	10.263714	17.344540
C	1.307859	6.876114	12.133360
C	0.934163	5.500979	12.524197
H	0.649368	7.709660	12.408765
H	1.855959	7.019774	11.190700
H	1.225359	4.647481	11.893591
H	0.020386	5.352959	13.123452
C	2.202950	6.233170	13.229449
C	3.533437	5.825873	12.803090
H	4.299142	5.563716	13.542481
H	3.612903	5.331380	11.820080

1a

SCF (BP86-D3) = -1527.546820

E(SCF)+ZPE(0 K)= -1526.940972

H(298 K)= -1526.904569

G(298 K)= -1527.009109

Lowest Frequency = 8.8793 cm-1

Si	3.599558	1.829524	3.225982
N	2.720734	2.902187	4.301866

C	1.794466	4.993191	5.163385
H	1.655979	6.078717	5.058502
H	1.348457	4.492420	6.032856
N	4.120799	3.027831	2.031206
C	2.510367	4.308384	4.217095
C	3.099400	4.976548	3.062446
H	2.925285	6.060695	3.003075
C	3.835605	4.409810	2.056236
C	4.378865	5.238669	0.920321
H	4.094853	6.301499	1.036346
H	4.000525	4.875924	-0.058496
H	5.485890	5.172046	0.866489
C	2.137593	2.221198	5.439231
C	2.900066	2.089077	6.630312
C	2.335383	1.363693	7.699943
H	2.913719	1.229473	8.628126
C	1.049710	0.810727	7.599692
H	0.627502	0.242852	8.444161
C	0.297249	0.988484	6.428613
H	-0.716167	0.561042	6.363885
C	0.818223	1.705926	5.332022
C	-0.017000	1.905508	4.068959
H	0.528733	2.620610	3.419833
C	-1.387769	2.536608	4.381192
H	-2.026796	1.861755	4.989487
H	-1.937160	2.753830	3.441056
H	-1.265543	3.488827	4.937165
C	-0.158670	0.583686	3.286427
H	0.836310	0.165086	3.025865
H	-0.722331	0.740625	2.342512
H	-0.698742	-0.180889	3.884858
C	4.293813	2.700036	6.760203
H	4.465260	3.330405	5.863619
C	4.400534	3.625399	7.988044
H	3.619885	4.412977	7.956370
H	5.392673	4.123373	8.012821
H	4.286921	3.065270	8.940535
C	5.379193	1.604030	6.771592
H	5.266439	0.936407	7.652435
H	6.394341	2.052614	6.810229
H	5.318069	0.971866	5.860797
C	4.911974	2.494063	0.941707
C	6.326246	2.441310	1.076445
C	7.069671	1.849624	0.034675
H	8.165607	1.782698	0.120503
C	6.434453	1.342575	-1.109109
H	7.032000	0.879130	-1.910253
C	5.040380	1.432574	-1.237671
H	4.553187	1.040616	-2.144381
C	4.253062	2.016092	-0.223515
C	2.734542	2.104205	-0.374091
H	2.361525	2.802173	0.404423
C	2.080988	0.731227	-0.110142
H	2.419492	-0.018338	-0.856930
H	0.974288	0.800903	-0.165918
H	2.349519	0.346343	0.896539
C	2.308496	2.675344	-1.740406
H	2.794935	3.652467	-1.941604
H	1.209263	2.826572	-1.768633
H	2.567198	1.990834	-2.575627
C	7.026749	2.981825	2.322984
H	6.300930	3.627654	2.860172
C	7.404799	1.828027	3.275826
H	8.125199	1.135128	2.791065
H	6.510450	1.234664	3.562293
H	7.869018	2.216788	4.206488
C	8.250914	3.854127	1.985214
H	9.070339	3.261084	1.527065
H	8.658652	4.318007	2.907399
H	7.986998	4.668347	1.278591

1b

SCF (BP86-D3) = -835.005493

E(SCF)+ZPE(0 K)= -834.554194

H(298 K)= -834.529777

G(298 K)= -834.604334

Lowest Frequency = 38.5594 cm-1

N	0.352016	6.973924	15.466658
C	0.140126	6.117039	14.496879
C	-0.548881	4.909850	15.130592
C	-0.427760	5.107733	16.677806
H	0.405926	4.487149	17.067088
H	-1.344188	4.799075	17.219993
C	-0.107443	6.600118	16.906017
C	-1.335741	7.437284	17.300732
H	-1.099500	8.520870	17.292369

H	-1.650011	7.167278	18.329746
H	-2.195382	7.259887	16.626624
C	1.006385	6.835513	17.935163
H	1.899406	6.214956	17.730170
H	0.633264	6.566078	18.944620
H	1.317488	7.899941	17.960301
C	-2.021518	4.924666	14.651931
H	-2.551159	5.843479	14.977771
H	-2.567545	4.049867	15.065269
H	-2.075114	4.881952	13.545004
C	0.133202	3.608388	14.666311
H	0.058271	3.494773	13.565397
H	-0.343851	2.725401	15.144187
H	1.210582	3.605325	14.935215
C	0.963242	8.264864	15.225236
C	2.372931	8.381662	15.348829
C	2.943207	9.662289	15.196941
H	4.033695	9.783316	15.287863
C	2.146078	10.780453	14.909546
H	2.608637	11.774865	14.805160
C	0.766145	10.624614	14.714805
H	0.157968	11.496635	14.428491
C	0.146191	9.364575	14.854111
C	-1.316108	9.182103	14.452169
H	-1.669270	8.221784	14.871039
C	-2.247112	10.288693	14.975630
H	-2.163850	10.406083	16.076145
H	-3.303449	10.047636	14.734828
H	-2.025319	11.273393	14.512614
C	-1.396635	9.038622	12.916015
H	-1.023067	9.954926	12.410913
H	-2.445527	8.868328	12.592083
H	-0.782325	8.176547	12.583645
C	3.265955	7.149365	15.480858
H	2.646807	6.318448	15.871625
C	3.728744	6.716723	14.071647
H	2.848215	6.503511	13.430503
H	4.354030	5.800193	14.126836
H	4.329475	7.517060	13.589145
C	4.449298	7.341328	16.443852
H	5.180588	8.084637	16.062022
H	4.997485	6.385192	16.574964
H	4.108194	7.683405	17.443047

4

SCF (BP86-D3) = -990.929756

E(SCF)+ZPE(0 K)= -990.390371

H(298 K)= -990.362318

G(298 K)= -990.442583

Lowest Frequency = 44.3895 cm-1

N	0.463334	7.028764	15.134152
C	0.186289	6.022855	14.132534
C	-0.973190	5.126637	14.708711
C	-1.061292	5.615300	16.192198
H	-1.179468	4.769783	16.900966
H	-1.947805	6.270849	16.321630
C	0.215948	6.452992	16.476824
C	-0.012341	7.538867	17.532769
H	0.888699	8.175939	17.647454
H	-0.228991	7.069434	18.513917
H	-0.862638	8.195727	17.269237
C	1.378606	5.535663	16.937900
H	1.661746	4.819847	16.140940
H	1.086891	4.952736	17.836723
H	2.275813	6.131150	17.197819
C	-2.304775	5.366933	13.970576
H	-2.544938	6.444815	13.901025
H	-3.140206	4.864555	14.503741
H	-2.269396	4.960259	12.939530
C	-0.661202	3.617634	14.655991
H	-0.579138	3.252633	13.611006
H	-1.480263	3.045308	15.140572
H	0.283364	3.370973	15.180743
C	0.798464	8.388048	14.856527
C	2.165732	8.793971	14.790569
C	2.479520	10.132703	14.474515
H	3.536047	10.442793	14.422588
C	1.473461	11.072433	14.217460
H	1.734770	12.112188	13.962654
C	0.130261	10.679315	14.293936
H	-0.661032	11.421740	14.099286
C	-0.229672	9.355424	14.619421
C	-1.712460	9.007005	14.700690
H	-1.769379	7.957823	15.049472
C	-2.462321	9.887612	15.721602
H	-1.968925	9.875392	16.715223

H	-3.505589	9.529904	15.851651
H	-2.513550	10.946317	15.389520
C	-2.400617	9.080889	13.322665
H	-2.362579	10.110890	12.908261
H	-3.469547	8.789466	13.401094
H	-1.919412	8.406593	12.587958
C	3.306156	7.818267	15.054115
H	2.840331	6.825905	15.205231
C	4.264112	7.709571	13.851278
H	3.711725	7.466583	12.920091
H	5.019581	6.913698	14.022717
H	4.814421	8.658441	13.675947
C	4.066392	8.178615	16.346145
H	4.580409	9.159414	16.253665
H	4.837932	7.413716	16.577455
H	3.377585	8.244878	17.213921
C	-0.317857	6.355216	11.389344
C	0.636812	7.480552	11.788109
H	-0.006485	5.659499	10.591439
H	-1.398558	6.574227	11.406082
H	0.210217	8.455831	12.071711
H	1.609329	7.544580	11.271395
C	0.422381	6.306025	12.688417
C	1.352856	5.345351	13.345546
H	2.373864	5.692957	13.564111
H	1.276523	4.276388	13.088330

5a

SCF (BP86-D3) = -990.939458

E(SCF)+ZPE(0 K)= -990.399301

H(298 K)= -990.371468

G(298 K)= -990.451455

Lowest Frequency = 33.6955 cm-1

N	0.840170	7.140203	15.077509
C	1.522097	6.017125	14.403120
C	2.060246	5.121450	15.610308
C	1.467544	5.821441	16.870678
H	2.231433	6.492393	17.316152
H	1.171757	5.090925	17.652319
C	0.291259	6.696387	16.386284
C	-1.009005	5.846206	16.302130
H	-1.890273	6.466354	16.054623
H	-1.206806	5.367783	17.284127
H	-0.934134	5.040234	15.548769
C	0.018067	7.883436	17.315780
H	0.920809	8.504763	17.455559
H	-0.310811	7.515171	18.308764
H	-0.779997	8.536362	16.908790
C	1.585396	3.655659	15.511854
H	0.482852	3.566557	15.455185
H	1.920582	3.090674	16.406874
H	2.014367	3.149552	14.622146
C	3.596719	5.097265	15.725382
H	4.068706	4.588981	14.860675
H	3.890645	4.548578	16.645588
H	4.019412	6.119294	15.804646
C	0.715799	8.468543	14.561590
C	1.799605	9.398822	14.705360
C	1.643126	10.719293	14.234723
H	2.473216	11.433992	14.353991
C	0.453718	11.149450	13.633062
H	0.351969	12.187974	13.279049
C	-0.603666	10.244297	13.488795
H	-1.540931	10.579483	13.015213
C	-0.495198	8.910071	13.935430
C	-1.704365	7.998700	13.744298
H	-1.407036	6.997443	14.104077
C	-2.904426	8.470454	14.592047
H	-2.629199	8.594306	15.659431
H	-3.739632	7.740298	14.535425
H	-3.289313	9.449304	14.234469
C	-2.115775	7.854553	12.265015
H	-2.460385	8.820000	11.837467
H	-2.951390	7.129915	12.164697
H	-1.274960	7.490948	11.640754
C	3.127666	9.039828	15.371289
H	3.126728	7.941966	15.515213
C	4.338528	9.424560	14.493819
H	4.202251	9.098498	13.444667
H	5.266342	8.956244	14.885566
H	4.504534	10.523094	14.491654
C	3.286018	9.699205	16.758584
H	3.265792	10.807039	16.676797
H	4.256267	9.411850	17.216693
H	2.482379	9.402877	17.460446
C	1.291029	6.221435	12.214442

C	0.615718	5.323419	13.283413
H	0.719937	7.158019	12.039106
H	1.554370	5.773068	11.233899
H	0.875812	4.259326	13.125982
H	-0.479241	5.416642	13.412019
C	2.414613	6.442901	13.205567
C	3.725163	6.633412	12.981108
H	4.479191	6.669483	13.776802
H	4.083620	6.773773	11.946879

5bE

SCF (BP86-D3) = -1221.846310

E(SCF)+ZPE(0 K)= -1221.226616

H(298 K)= -1221.194023

G(298 K)= -1221.285917

Lowest Frequency = 27.5789 cm-1

N	0.798873	7.003671	15.124213
C	1.337670	5.716326	14.634855
C	1.818829	4.979418	15.968682
C	1.345528	5.939468	17.101845
H	2.189721	6.589013	17.414161
H	1.003280	5.385433	18.000715
C	0.250308	6.843958	16.496232
C	-1.133675	6.137559	16.577243
H	-1.953629	6.801684	16.246864
H	-1.345008	5.849519	17.628263
H	-1.170733	5.216623	15.966426
C	0.132828	8.190666	17.217207
H	1.100770	8.722309	17.241921
H	-0.205148	8.030506	18.261195
H	-0.601812	8.851603	16.714788
C	1.185437	3.579547	16.125148
H	0.078570	3.605219	16.098000
H	1.484840	3.138398	17.098949
H	1.530082	2.887220	15.329158
C	3.346043	4.798970	16.068683
H	3.735405	4.115726	15.287080
H	3.602648	4.364553	17.058395
H	3.882312	5.766264	15.989556
C	0.793309	8.228580	14.383924
C	1.970204	9.050523	14.345152
C	1.939067	10.267936	13.633177
H	2.841972	10.898807	13.608922
C	0.785802	10.701509	12.967375
H	0.783759	11.658857	12.421803
C	-0.364065	9.905093	13.006647
H	-1.274349	10.244659	12.485985
C	-0.382851	8.674557	13.697156
C	-1.688355	7.884029	13.697744
H	-1.485753	6.936671	14.228283
C	-2.795318	8.631905	14.470396
H	-2.465619	8.909764	15.492374
H	-3.706654	8.003513	14.562611
H	-3.084815	9.569873	13.950249
C	-2.173468	7.524662	12.278821
H	-2.433367	8.429947	11.690191
H	-3.083342	6.890014	12.330258
H	-1.401921	6.965232	11.712485
C	3.271856	8.688321	15.060188
H	3.168038	7.639661	15.400745
C	4.497764	8.793557	14.125517
H	4.304928	8.342210	13.133970
H	5.374408	8.279864	14.573828
H	4.790715	9.852724	13.962486
C	3.521248	9.567559	16.305136
H	3.600598	10.639378	16.023695
H	4.471902	9.277039	16.800819
H	2.711003	9.479925	17.054593
C	1.050053	5.570730	12.448855
C	0.324554	4.947106	13.667558
H	0.555998	6.500302	12.092593
H	1.253009	4.921614	11.570864
H	0.464512	3.849836	13.689089
H	-0.749063	5.180500	13.796275
C	2.216736	5.865529	13.367946
C	3.547463	5.931183	13.128994
C	4.207877	5.940002	11.812956
C	3.562663	6.356236	10.618484
C	5.571514	5.553847	11.728108
C	4.245016	6.351346	9.391618
H	2.527401	6.723338	10.657742
C	6.251565	5.545435	10.500943
H	6.098247	5.249446	12.647698
C	5.588927	5.938874	9.323127
H	3.724551	6.685118	8.479539
H	7.307714	5.233706	10.462670

H	6.121212	5.937251	8.358753
H	4.235011	5.972639	13.984498

5bZ

SCF (BP86-D3) = -1221.846993
E(SCF)+ZPE(0 K)= -1221.227348
H(298 K)= -1221.194819
G(298 K)= -1221.285925
Lowest Frequency = 16.5217 cm-1

N	0.969353	7.161494	15.022984
C	1.580130	6.108889	14.184965
C	1.523932	4.844804	15.112627
C	1.630530	5.458684	16.525897
H	2.698365	5.590758	16.785673
H	1.192365	4.796905	17.300889
C	0.920381	6.833060	16.484154
C	-0.524961	6.747442	17.026600
H	-1.024180	7.731560	16.933497
H	-0.512030	6.480421	18.103567
H	-1.136389	5.994573	16.497440
C	1.642214	7.868323	17.370829
H	2.709376	7.982273	17.108547
H	1.589042	7.560290	18.435439
H	1.159550	8.862804	17.281043
C	0.162095	4.131042	14.924279
H	-0.683688	4.844638	14.896673
H	-0.015827	3.421204	15.758476
H	0.135213	3.549032	13.980881
C	2.637151	3.819785	14.845534
H	2.630217	3.490601	13.785043
H	2.482923	2.920441	15.478797
H	3.642158	4.224161	15.068424
C	0.518641	8.428709	14.510160
C	1.423210	9.540673	14.393327
C	0.944361	10.788008	13.940394
H	1.646057	11.633942	13.860446
C	-0.396955	10.975660	13.590763
H	-0.753212	11.959554	13.245530
C	-1.277242	9.892158	13.675336
H	-2.330458	10.033753	13.384047
C	-0.852311	8.620356	14.117702
C	-1.908272	7.511669	14.110673
H	-1.408109	6.587990	14.462286
C	-3.097662	7.811712	15.048907
H	-2.773089	8.052625	16.078805
H	-3.779741	6.936856	15.101239
H	-3.692484	8.672877	14.677096
C	-2.456866	7.252673	12.687659
H	-3.066851	8.108847	12.330248
H	-3.108684	6.353685	12.680978
H	-1.649673	7.095633	11.944174
C	2.912853	9.451249	14.715174
H	3.102899	8.415470	15.048249
C	3.789165	9.705205	13.469791
H	3.489978	9.068766	12.616307
H	4.851922	9.481299	13.700105
H	3.728500	10.765689	13.143650
C	3.341083	10.417396	15.840877
H	3.297637	11.474458	15.502211
H	4.388986	10.209621	16.144667
H	2.700238	10.330215	16.738880
C	2.100914	6.709123	12.120824
C	0.929340	5.937033	12.764323
H	1.848831	7.786904	11.996212
H	2.530169	6.334114	11.167677
H	0.931120	4.872203	12.458981
H	-0.085633	6.342055	12.635119
C	2.901610	6.471962	13.387339
C	4.255393	6.411112	13.462685
H	4.767273	6.572748	12.493292
C	5.181124	6.159283	14.577897
C	6.429366	5.534797	14.318813
C	4.908908	6.552353	15.909661
C	7.340320	5.276626	15.354944
H	6.676383	5.238053	13.286109
C	5.818252	6.301596	16.947817
H	3.970374	7.078976	16.119379
C	7.037879	5.653764	16.677531
H	8.297274	4.779325	15.129135
H	5.574354	6.622025	17.973432
H	7.754402	5.454451	17.489971

5d

SCF (BP86-D3) = -1559.009522
E(SCF)+ZPE(0 K)= -1558.385908

H(298 K)= -1558.349466

G(298 K)= -1558.452177

Lowest Frequency = 11.0183 cm-1

N	0.797030	6.990153	15.127838
C	1.304275	5.672602	14.692575
C	1.771692	4.979079	16.054101
C	1.321544	5.995023	17.147528
H	2.180876	6.636411	17.434131
H	0.966888	5.485216	18.067350
C	0.247215	6.899674	16.506180
C	-1.153129	6.231151	16.616957
H	-1.956809	6.899606	16.256797
H	-1.370793	5.996002	17.679722
H	-1.213539	5.284557	16.048609
C	0.163328	8.277662	17.170205
H	1.143410	8.786999	17.170920
H	-0.175366	8.169248	18.220518
H	-0.557119	8.933843	16.641824
C	1.106704	3.601430	16.266078
H	0.000775	3.651321	16.236637
H	1.395782	3.193537	17.257216
H	1.435698	2.869728	15.499247
C	3.294636	4.768498	16.160823
H	3.667838	4.043523	15.409424
H	3.542067	4.370175	17.167758
H	3.852947	5.719256	16.042317
C	0.820963	8.183664	14.337591
C	2.017691	8.974069	14.265714
C	2.016309	10.160246	13.502130
H	2.934031	10.767712	13.452043
C	0.873985	10.592703	12.817078
H	0.895369	11.525572	12.231116
C	-0.295139	9.827270	12.889206
H	-1.196836	10.166757	12.353905
C	-0.344218	8.628227	13.631625
C	-1.668978	7.870997	13.665378
H	-1.489667	6.941193	14.234132
C	-2.755260	8.677550	14.407921
H	-2.416981	8.988069	15.417589
H	-3.682083	8.076842	14.525955
H	-3.021455	9.600756	13.850369
C	-2.166061	7.466878	12.262652
H	-2.404451	8.353826	11.638198
H	-3.091465	6.858064	12.340879
H	-1.410644	6.865653	11.717704
C	3.310349	8.611460	14.997045
H	3.182292	7.580585	15.381099
C	4.539453	8.650027	14.061153
H	4.335878	8.168701	13.086004
H	5.402449	8.131280	14.529621
H	4.859697	9.694324	13.859093
C	3.580180	9.535256	16.205008
H	3.683347	10.592466	15.879724
H	4.524330	9.243991	16.712440
H	2.768736	9.496614	16.957167
C	1.009695	5.449558	12.514069
C	0.273592	4.887537	13.756317
H	0.536256	6.377323	12.126851
H	1.195565	4.765252	11.659498
H	0.390298	3.789237	13.819371
H	-0.794160	5.149494	13.876208
C	2.183886	5.752324	13.419781
C	3.516268	5.773581	13.179000
C	4.167159	5.713612	11.861871
C	3.525930	6.105104	10.656498
C	5.519308	5.287189	11.782888
C	4.193695	6.036629	9.427569
H	2.503842	6.506418	10.685886
C	6.191517	5.212704	10.557438
H	6.046871	5.003482	12.707595
C	5.527465	5.584921	9.371509
H	3.683494	6.352605	8.505386
H	7.237077	4.872738	10.518065
H	4.208076	5.827645	14.029841
C	6.225381	5.458099	8.040151
F	5.794209	6.386432	7.132593
F	6.008100	4.227444	7.463734
F	7.581857	5.598275	8.143668

5e

SCF (BP86-D3) = -1378.995019

E(SCF)+ZPE(0 K)= -1378.266933

H(298 K)= -1378.228624

G(298 K)= -1378.333481

Lowest Frequency = 16.9524 cm-1

N	0.810037	6.990063	15.136475
C	1.297989	5.626929	14.832463
C	1.782008	5.077268	16.252817
C	1.362345	6.208210	17.239839
H	2.232845	6.867164	17.440376
H	1.021098	5.803765	18.215580
C	0.284314	7.051543	16.525148
C	-1.119484	6.412775	16.732920
H	-1.925422	7.049768	16.323995
H	-1.315757	6.288726	17.818471
H	-1.200095	5.414215	16.264808
C	0.224908	8.492298	17.042267
H	1.210181	8.987008	16.974279
H	-0.097326	8.498723	18.103358
H	-0.497595	9.097897	16.458988
C	1.108040	3.737882	16.622580
H	0.002228	3.796590	16.606242
H	1.410929	3.434699	17.646760
H	1.416358	2.924893	15.932868
C	3.304459	4.861670	16.354331
H	3.656464	4.060370	15.673631
H	3.567450	4.566149	17.392443
H	3.868659	5.788876	16.127511
C	0.820980	8.090107	14.220794
C	2.018659	8.861299	14.038536
C	2.005820	9.956964	13.149985
H	2.924780	10.549552	13.015136
C	0.851325	10.319181	12.444898
H	0.864260	11.182229	11.759933
C	-0.319022	9.573619	12.624436
H	-1.230661	9.858445	12.074119
C	-0.356850	8.463022	13.494328
C	-1.683992	7.723256	13.637710
H	-1.497665	6.862168	14.303736
C	-2.752220	8.615146	14.304947
H	-2.392880	9.035162	15.266660
H	-3.679165	8.037512	14.507550
H	-3.025452	9.471423	13.652060
C	-2.209280	7.166124	12.299314
H	-2.455992	7.978027	11.582719
H	-3.135469	6.575620	12.463041
H	-1.466104	6.502552	11.813202
C	3.324254	8.575750	14.780822
H	3.200359	7.595149	15.280735

C	4.534548	8.502662	13.822852
H	4.311933	7.911759	12.914512
H	5.405907	8.038182	14.331123
H	4.851745	9.516137	13.496368
C	3.620206	9.629010	15.871027
H	3.724056	10.641510	15.425620
H	4.571388	9.390176	16.392611
H	2.821588	9.683081	16.635938
C	0.965472	5.167649	12.696566
C	0.242429	4.759081	14.004510
H	0.494080	6.048150	12.208563
H	1.132188	4.385610	11.925818
H	0.345846	3.672954	14.187953
H	-0.820294	5.046915	14.110929
C	2.156927	5.559534	13.545242
C	3.484507	5.555988	13.280077
C	4.126658	5.362459	11.970830
C	3.473207	5.577678	10.728852
C	5.490522	4.984203	11.919546
C	4.147358	5.390869	9.516015
H	2.433282	5.932817	10.711255
C	6.158994	4.794312	10.699980
H	6.038628	4.830360	12.864051
C	5.504225	4.987070	9.463481
H	3.597311	5.578864	8.579980
H	7.216001	4.493446	10.728047
H	4.186113	5.706926	14.111632
C	6.199963	4.795181	8.100858
C	6.174928	6.135989	7.322119
H	6.671589	6.020503	6.335111
H	5.139322	6.487818	7.137666
H	6.707434	6.930051	7.886040
C	5.442535	3.711622	7.290526
H	5.926068	3.557973	6.302220
H	5.444002	2.741653	7.830252
H	4.386181	3.995667	7.106793
C	7.669355	4.349199	8.252739
H	8.275064	5.100789	8.800551
H	7.753598	3.379840	8.786779
H	8.128172	4.218749	7.250707

C3-H2

SCF (BP86-D3) = -155.851107

E(SCF)+ZPE(0 K)= -155.769173

H(298 K)= -155.763830

G(298 K)= -155.795307

Lowest Frequency = 282.7548 cm-1

C	-5.354879	0.769898	0.000966
C	-4.581719	2.108689	-0.000520
H	-5.879574	0.472893	-0.925578
H	-5.879987	0.474690	0.927845
H	-4.587581	2.712531	0.925404
H	-4.587618	2.710120	-0.928012
C	-3.880490	0.811069	0.001261
C	-2.724078	0.143246	0.003879
H	-1.756537	0.673703	0.003932
H	-2.699966	-0.959904	0.005800

C3-PhCF3

SCF (BP86-D3) = -723.919666

E(SCF)+ZPE(0 K)= -723.753939

H(298 K)= -723.740455

G(298 K)= -723.795239

Lowest Frequency = 10.6216 cm-1

C	-5.625528	1.329608	0.098052
C	-4.847756	2.296452	1.017364
H	-6.305731	1.764941	-0.657130
H	-5.986807	0.378432	0.530755
H	-4.679462	1.997666	2.068691
H	-4.998650	3.383418	0.880929
C	-4.176711	1.535306	-0.050883
C	-3.046250	1.227227	-0.712523
H	-3.116120	0.500299	-1.541710
C	-1.705437	1.767867	-0.439887
C	-0.609336	1.340828	-1.231953
C	-1.452988	2.711592	0.591818
C	0.685401	1.829117	-1.009748
H	-0.780622	0.611021	-2.039508
C	-0.163964	3.203017	0.818299
H	-2.283959	3.062427	1.220558
C	0.911971	2.765860	0.015970
H	1.521948	1.486701	-1.636541
H	0.014191	3.936322	1.619341
C	2.305006	3.267466	0.305010
F	2.315628	4.586616	0.672572
F	2.893198	2.578354	1.340573
F	3.143280	3.143413	-0.766723

C3-Ph

SCF (BP86-D3) = -386.756536

E(SCF)+ZPE(0 K)= -386.594843

H(298 K)= -386.585123

G(298 K)= -386.628983

Lowest Frequency = 51.4523 cm-1

C	-5.627617	1.333688	0.083644
C	-4.854743	2.293843	1.014885
H	-6.305213	1.775949	-0.670201
H	-5.994203	0.381061	0.509235
H	-4.695240	1.987956	2.065737
H	-5.006627	3.381814	0.886281
C	-4.176924	1.540200	-0.054595
C	-3.041887	1.236757	-0.709729
H	-3.109766	0.514912	-1.544205
C	-1.697946	1.771262	-0.430328
C	-0.601743	1.342840	-1.221655
C	-1.444080	2.707223	0.607092
C	0.695899	1.826972	-0.989357
H	-0.777466	0.616411	-2.032430
C	-0.148309	3.189977	0.838201
H	-2.277442	3.055527	1.235345
C	0.929718	2.753658	0.042356
H	1.530524	1.478207	-1.618452
H	0.025584	3.915893	1.648813
H	1.946323	3.135374	0.227119

C3-PhtBu

SCF (BP86-D3) = -543.905140

E(SCF)+ZPE(0 K)= -543.634775

H(298 K)= -543.619481

G(298 K)= -543.675784

Lowest Frequency = 33.6205 cm-1

C	-5.654356	1.296215	0.146200
C	-4.827078	2.095859	1.177435
H	-6.372704	1.851896	-0.485017

H	-5.999796	0.284806	0.430740
H	-4.610394	1.622845	2.153533
H	-4.983286	3.189213	1.237808
C	-4.213070	1.531767	-0.037503
C	-3.117166	1.348202	-0.796039
H	-3.232860	0.772912	-1.733026
C	-1.759954	1.839020	-0.507870
C	-0.706088	1.563464	-1.410073
C	-1.438119	2.591969	0.651858
C	0.603373	2.014050	-1.172141
H	-0.918089	0.980871	-2.322224
C	-0.132726	3.037151	0.882351
H	-2.228345	2.828627	1.380180
C	0.926540	2.762854	-0.020398
H	1.379357	1.767777	-1.910859
H	0.067293	3.618089	1.797025
C	2.348453	3.277281	0.281080
C	2.320704	4.824609	0.380222
H	1.645778	5.175483	1.187510
H	3.337039	5.217140	0.597482
H	1.972698	5.275064	-0.572729
C	2.831545	2.681041	1.628636
H	2.855581	1.572193	1.583751
H	3.855806	3.039853	1.866026
H	2.170461	2.972096	2.470408
C	3.358933	2.879698	-0.815251
H	3.445090	1.777886	-0.917691
H	3.081642	3.299595	-1.804558
H	4.365462	3.270028	-0.558007

Int-S1-2

SCF (BP86-D3) = -1558.971390

E(SCF)+ZPE(0 K)= -1558.349989

H(298 K)= -1558.312843

G(298 K)= -1558.416624

Lowest Frequency = 10.6725 cm-1

N	0.280738	7.272229	14.970627
C	-0.232993	6.467161	14.020504
C	-0.626814	5.116994	14.648754
C	-0.183470	5.294814	16.129284
H	0.826104	4.858149	16.274708
H	-0.867780	4.792048	16.839720
C	-0.112550	6.811521	16.372580
C	-1.488906	7.415462	16.724628
H	-1.403527	8.496536	16.938424
H	-1.886018	6.918922	17.632990
H	-2.224689	7.285113	15.908672
C	0.886129	7.216393	17.457274
H	1.849796	6.687298	17.367314
H	0.453940	6.959502	18.444760
H	1.075514	8.308478	17.441377
C	-2.138749	4.779573	14.573667
H	-2.763454	5.553268	15.059168
H	-2.316786	3.821335	15.103459
H	-2.498799	4.655260	13.536021
C	0.198114	3.992359	13.976652
H	-0.145726	3.783335	12.945631
H	0.091186	3.059414	14.568641
H	1.275217	4.254633	13.931149
C	1.102373	8.443447	14.741273
C	2.518212	8.255730	14.760799
C	3.327186	9.373623	14.483066
H	4.421017	9.250633	14.464620
C	2.772537	10.638298	14.237955
H	3.428438	11.497538	14.027140
C	1.383493	10.805463	14.271580
H	0.953584	11.802016	14.085388
C	0.518333	9.716825	14.512014
C	-0.987371	9.951955	14.445233
H	-1.487266	8.965827	14.517332
C	-1.471524	10.834755	15.615282
H	-1.152451	10.434298	16.599139
H	-2.578630	10.917338	15.616519
H	-1.056466	11.861311	15.531272
C	-1.405001	10.580809	13.097775
H	-1.075442	11.638405	13.025278
H	-2.509595	10.569221	12.989344
H	-0.965587	10.034000	12.241444
C	3.205218	6.927405	15.089595
H	2.422201	6.205320	15.395655
C	3.936820	6.300302	13.888169
H	3.238669	6.023442	13.079092
H	4.477786	5.386006	14.210940
H	4.680593	6.999866	13.454403
C	4.190747	7.096701	16.270013
H	5.095429	7.657235	15.954716

H	4.530707	6.103995	16.632088
H	3.743066	7.644746	17.123445
C	-1.198470	6.029354	11.622433
C	-1.778219	7.296360	12.158337
H	-0.822275	6.020605	10.588733
H	-1.632234	5.073406	11.946103
H	-2.585142	7.226580	12.907409
H	-1.809889	8.187553	11.512737
C	-0.345991	6.857423	12.590978
C	0.834320	7.557255	12.006418
H	0.895932	8.649122	12.120266
C	1.885536	6.927335	11.311429
C	1.974179	5.506368	11.048931
C	2.985530	7.718957	10.801823
C	3.053976	4.946712	10.371838
H	1.182953	4.841166	11.420200
C	4.059800	7.153615	10.129394
H	2.969679	8.807064	10.980121
C	4.119431	5.751732	9.895650
H	3.084770	3.858212	10.205286
H	4.880869	7.795677	9.772512
C	5.314499	5.142895	9.258183
F	6.347442	4.865421	10.156726
F	5.893867	5.953648	8.306881
F	5.050353	3.939915	8.645554

Int-S1-3

SCF (BP86-D3) = -1378.975853

E(SCF)+ZPE(0 K)= -1378.248278

H(298 K)= -1378.209902

G(298 K)= -1378.313340

Lowest Frequency = 22.2687 cm-1

N	0.364992	7.532413	14.900732
C	0.410654	6.627997	13.755505
C	0.455502	5.160752	14.336952
C	0.184030	5.406927	15.858049
H	1.132360	5.320531	16.428218
H	-0.513111	4.652605	16.277806
C	-0.348435	6.854856	16.010194
C	-1.884560	6.894951	15.807200
H	-2.279956	7.918694	15.951463
H	-2.395446	6.225718	16.530941
H	-2.158553	6.564975	14.785825
C	0.000742	7.457630	17.374678
H	1.088886	7.418212	17.570793
H	-0.517234	6.893576	18.176798
H	-0.317075	8.517618	17.438801
C	-0.637405	4.239912	13.756834
H	-1.652421	4.662327	13.892910
H	-0.607823	3.257419	14.273179
H	-0.488311	4.050060	12.676234
C	1.818246	4.457052	14.158182
H	1.996089	4.152714	13.109801
H	1.850566	3.541727	14.787148
H	2.659649	5.106007	14.459043
C	1.106558	8.749557	15.027775
C	2.503548	8.733832	15.349040
C	3.215123	9.949744	15.411692
H	4.292075	9.930942	15.645959
C	2.579286	11.181094	15.208507
H	3.151481	12.120828	15.269626
C	1.205294	11.203580	14.941815
H	0.697598	12.171279	14.799587
C	0.457306	10.011429	14.839857
C	-1.031991	10.117045	14.542728
H	-1.376940	9.086265	14.342048
C	-1.811127	10.658684	15.758935
H	-1.633158	10.045884	16.666138
H	-2.903784	10.662536	15.558212
H	-1.506642	11.700533	15.997475
C	-1.321109	10.969796	13.291453
H	-1.125042	12.048240	13.473136
H	-2.386003	10.874361	12.992033
H	-0.693185	10.654526	12.434234
C	3.256125	7.450668	15.680508
H	2.508426	6.636600	15.670676
C	4.331601	7.114037	14.630731
H	3.889690	6.984287	13.624579
H	4.856600	6.171182	14.894191
H	5.097726	7.915972	14.566149
C	3.873754	7.495890	17.094264
H	4.699623	8.235782	17.157817
H	4.294605	6.504218	17.364092
H	3.122339	7.770199	17.863258
C	-1.344683	6.432030	11.518820
C	-1.537605	7.828053	12.102247

H	-1.005838	6.356716	10.470699
H	-2.056166	5.647817	11.821350
H	-2.364061	7.965665	12.820068
H	-1.353110	8.697749	11.451017
C	-0.376248	6.990054	12.516454
C	1.109940	7.190212	12.482884
H	1.430197	8.235466	12.646812
C	2.007205	6.470245	11.530950
C	1.683682	5.239625	10.919045
C	3.238749	7.061789	11.161735
C	2.564669	4.610765	10.022502
H	0.725515	4.753519	11.150715
C	4.115729	6.432611	10.267497
H	3.512291	8.042646	11.584052
C	3.808869	5.182834	9.679113
H	2.257278	3.648577	9.588476
H	5.064386	6.936796	10.022595
C	4.808245	4.516095	8.711941
C	4.291199	3.163269	8.179268
H	4.117526	2.435792	8.999386
H	3.344560	3.277325	7.611104
H	5.040422	2.718192	7.492030
C	5.055434	5.454037	7.502478
H	5.775047	4.992071	6.793216
H	4.109631	5.650833	6.955758
H	5.474818	6.431650	7.816687
C	6.145721	4.267631	9.456103
H	6.594178	5.212348	9.826054
H	5.992875	3.600429	10.330032
H	6.884050	3.787145	8.779090

Int-S1E

SCF (BP86-D3) = -1221.828919

E(SCF)+ZPE(0 K)= -1221.210320

H(298 K)= -1221.177446

G(298 K)= -1221.268976

Lowest Frequency = 33.2556 cm-1

N	1.229165	7.232993	14.860056
C	1.628936	6.284694	13.851355
C	1.267930	4.856436	14.416713
C	0.820500	5.188376	15.878694
H	1.174476	4.425861	16.602008
H	-0.287524	5.207753	15.944149
C	1.353393	6.606396	16.199671
C	0.511456	7.329156	17.254649
H	0.855968	8.374307	17.392680
H	0.603302	6.805156	18.227647
H	-0.559002	7.357479	16.977752
C	2.828664	6.553603	16.673241
H	3.494971	6.183430	15.869029
H	2.936298	5.877342	17.546827
H	3.184446	7.557612	16.977457
C	0.113494	4.224037	13.614195
H	-0.741484	4.918991	13.504998
H	-0.253576	3.307068	14.122928
H	0.449760	3.934717	12.597742
C	2.428155	3.839556	14.467177
H	2.732638	3.496667	13.462287
H	2.099646	2.946407	15.039240
H	3.328089	4.252402	14.961985
C	0.660295	8.519701	14.606059
C	1.484184	9.685125	14.607226
C	0.910341	10.939496	14.309827
H	1.548171	11.838361	14.306771
C	-0.452433	11.062450	14.012276
H	-0.882763	12.047853	13.771658
C	-1.266697	9.921730	14.034504
H	-2.342117	10.022137	13.814612
C	-0.740201	8.649976	14.339176
C	-1.696705	7.461170	14.377042
H	-1.102620	6.588095	14.707164
C	-2.835419	7.670822	15.397368
H	-2.445121	7.940911	16.400025
H	-3.438842	6.744502	15.501954
H	-3.524190	8.482254	15.079949
C	-2.281392	7.129050	12.989365
H	-2.874085	7.977893	12.586698
H	-2.955023	6.248230	13.050344
H	-1.488433	6.895572	12.252959
C	2.973292	9.623170	14.929203
H	3.224473	8.552439	15.055860
C	3.840049	10.176586	13.780359
H	3.596848	9.681392	12.817551
H	4.917887	10.010549	13.989413
H	3.690985	11.268329	13.641820
C	3.290864	10.333302	16.260447

H	3.080730	11.422495	16.198193
H	4.362173	10.210690	16.526652
H	2.681601	9.923782	17.092606
C	1.072483	7.787470	11.570124
C	1.060571	6.338717	11.101760
H	0.122561	8.253324	11.872097
H	1.785586	8.478867	11.091146
H	1.735718	6.057918	10.277165
H	0.095886	5.805242	11.101210
C	1.663153	6.706747	12.425816
C	3.008099	6.583954	13.057958
C	4.088637	5.644666	12.663646
C	3.941450	4.636459	11.679215
C	5.352028	5.766661	13.296166
C	5.004145	3.775449	11.358682
H	2.973268	4.512449	11.173861
C	6.414354	4.906129	12.978900
H	5.493511	6.554206	14.055459
C	6.246262	3.900095	12.008156
H	4.859513	2.996909	10.592017
H	7.383175	5.022026	13.491474
H	7.078089	3.223427	11.756229
H	3.410754	7.544845	13.411771

Int-S1Z

SCF (BP86-D3) = -1221.824135

E(SCF)+ZPE(0 K)= -1221.205139

H(298 K)= -1221.172551

G(298 K)= -1221.262844

Lowest Frequency = 36.1805 cm-1

N	1.416511	7.418484	14.844041
C	1.673568	6.422193	13.818144
C	1.337458	4.994173	14.426607
C	0.840189	5.364603	15.854338
H	1.117671	4.596271	16.605436
H	-0.266050	5.438134	15.858857
C	1.426494	6.760394	16.189986
C	0.582583	7.513547	17.227114
H	1.011863	8.511274	17.444660
H	0.562490	6.933120	18.171825
H	-0.458287	7.669473	16.893506
C	2.862058	6.647826	16.752268
H	3.534485	6.125091	16.049104
H	2.863643	6.075050	17.702979
H	3.288111	7.650591	16.949102
C	0.263559	4.223732	13.630918
H	-0.681997	4.790510	13.526740
H	0.024651	3.266945	14.142048
H	0.623207	3.981299	12.610190
C	2.572638	4.063480	14.509929
H	2.877620	3.696004	13.508672
H	2.329135	3.170479	15.122675
H	3.452219	4.558732	14.963704
C	0.659383	8.625642	14.643287
C	1.323654	9.871682	14.861517
C	0.593105	11.077297	14.787686
H	1.113085	12.032399	14.967178
C	-0.772405	11.082901	14.485370
H	-1.331050	12.031275	14.434309
C	-1.420861	9.864424	14.241629
H	-2.495776	9.867128	13.999057
C	-0.738938	8.632684	14.316178
C	-1.549947	7.365182	14.049941
H	-0.825405	6.538863	13.959993
C	-2.517566	7.024859	15.204289
H	-1.996441	6.898337	16.172629
H	-3.060421	6.079973	14.989424
H	-3.273433	7.828451	15.334249
C	-2.357126	7.421333	12.733744
H	-3.217745	8.118719	12.812150
H	-2.772525	6.418604	12.498344
H	-1.742909	7.747046	11.872692
C	2.817338	9.949673	15.152143
H	3.199815	8.913021	15.093695
C	3.551152	10.773994	14.077790
H	3.360756	10.364393	13.065756
H	4.646903	10.741615	14.246426
H	3.236113	11.839697	14.087058
C	3.115562	10.495097	16.562233
H	2.785766	11.551058	16.670025
H	4.206443	10.462308	16.769447
H	2.602380	9.905761	17.349910
C	0.977362	7.883134	11.536080
C	0.735148	6.422695	11.161036
H	0.138136	8.471208	11.935201
H	1.674778	8.475028	10.922693

H	1.270014	6.009656	10.288642
H	-0.279712	6.020288	11.309643
C	1.546192	6.776926	12.367647
C	2.921765	6.468572	12.850743
C	4.076419	7.424985	12.834097
C	4.386060	8.159486	11.665802
C	4.974522	7.506780	13.921400
C	5.539000	8.960816	11.592701
H	3.726619	8.080656	10.788575
C	6.126109	8.308770	13.856172
H	4.765061	6.927517	14.830646
C	6.412847	9.042654	12.690815
H	5.757301	9.521346	10.669496
H	6.805170	8.359696	14.722434
H	7.315046	9.672529	12.638169
H	3.256547	5.448899	12.585220

TS-S1-2

SCF (BP86-D3) = -1558.931430

E(SCF)+ZPE(0 K)= -1558.311728

H(298 K)= -1558.274723

G(298 K)= -1558.377786

Lowest Frequency = -146.8059 cm-1

N	0.198299	7.210205	15.158824
C	-0.155289	6.272521	14.322601
C	-0.708544	5.089152	15.115128
C	-0.377255	5.444420	16.603991
H	0.569470	4.947655	16.902516
H	-1.163143	5.103851	17.306448
C	-0.190975	6.971979	16.650223
C	-1.509087	7.703479	16.956384
H	-1.371808	8.799519	16.975786
H	-1.867496	7.393111	17.958854
H	-2.299377	7.461682	16.220218
C	0.894076	7.457604	17.614181
H	1.851111	6.919368	17.482235
H	0.551850	7.289635	18.655437
H	1.082276	8.543073	17.488171
C	-2.223675	4.878686	14.885421
H	-2.819428	5.761519	15.190481
H	-2.570253	4.011564	15.484838
H	-2.447307	4.668123	13.821574
C	0.058004	3.820677	14.672219
H	-0.151492	3.569659	13.613264
H	-0.246688	2.959184	15.303768
H	1.153689	3.955869	14.776102
C	1.042267	8.328458	14.760517
C	2.438933	8.064532	14.699456
C	3.281964	9.100061	14.252764
H	4.362401	8.914142	14.161892
C	2.767467	10.358627	13.911362
H	3.444556	11.155543	13.565677
C	1.390829	10.597166	14.001062
H	0.993275	11.584463	13.718783
C	0.494180	9.586948	14.411790
C	-1.002876	9.884121	14.396052
H	-1.536313	8.940532	14.625697
C	-1.373186	10.939768	15.460253
H	-0.991007	10.671785	16.466588
H	-2.474378	11.062660	15.528309
H	-0.939376	11.928508	15.200683
C	-1.482930	10.345597	13.003402
H	-1.067260	11.340685	12.739457
H	-2.589465	10.433715	12.988386
H	-1.182519	9.630788	12.215121
C	3.037386	6.701416	15.055422
H	2.306388	6.165529	15.692284
C	3.232453	5.833813	13.799674
H	2.263254	5.657182	13.296062
H	3.675926	4.851804	14.068706
H	3.896283	6.329207	13.061400
C	4.338486	6.819336	15.871588
H	5.175589	7.217054	15.260817
H	4.650818	5.818700	16.235848
H	4.213542	7.485464	16.750803
C	-1.038237	5.734509	11.567115
C	-1.728978	7.001504	12.085612
H	-0.972342	5.588174	10.475027
H	-1.177440	4.801363	12.135670
H	-2.338378	6.919586	13.001383
H	-2.135305	7.711162	11.344858
C	-0.241568	6.849841	12.152662
C	0.813567	7.615827	11.679849
H	0.821157	8.690221	11.925763
C	2.004333	7.118341	11.036975
C	2.186903	5.755885	10.635301

C	3.098631	8.006989	10.791628
C	3.378325	5.316505	10.058003
H	1.379916	5.030536	10.812514
C	4.291570	7.570532	10.214113
H	2.998574	9.060239	11.100140
C	4.448055	6.216085	9.837927
H	3.491164	4.259546	9.770623
H	5.118248	8.279733	10.053154
C	5.751821	5.723825	9.295575
F	6.614938	5.282687	10.288424
F	6.447316	6.690382	8.612922
F	5.608554	4.659072	8.440755

TS-S1-3

SCF (BP86-D3) = -1378.909234

E(SCF)+ZPE(0 K)= -1378.185185

H(298 K)= -1378.146386

G(298 K)= -1378.251440

Lowest Frequency = -194.1396 cm-1

N	0.238797	7.233617	15.122000
C	-0.126243	6.280224	14.308037
C	-0.624637	5.087223	15.126071
C	-0.251917	5.469499	16.597997
H	0.721500	5.008980	16.867163
H	-0.999865	5.110786	17.332034
C	-0.116297	7.002085	16.620948
C	-1.453108	7.694706	16.937706
H	-1.348572	8.794332	16.947750
H	-1.790254	7.380406	17.946263
H	-2.243977	7.424958	16.212127
C	0.965912	7.535665	17.562062
H	1.938398	7.028340	17.422040
H	0.643702	7.368121	18.609662
H	1.115211	8.625161	17.420666
C	-2.138479	4.811896	14.968093
H	-2.756459	5.671384	15.293896
H	-2.416058	3.938702	15.593717
H	-2.405590	4.577096	13.920009
C	0.173749	3.842917	14.667588
H	-0.063496	3.572486	13.619638
H	-0.075907	2.979045	15.319741
H	1.266300	4.020846	14.730424
C	1.053490	8.367597	14.704846
C	2.454817	8.136048	14.631747
C	3.267720	9.191348	14.176109
H	4.350934	9.030489	14.071870
C	2.720551	10.438254	13.843574
H	3.374944	11.250701	13.490416
C	1.340277	10.645991	13.952114
H	0.916863	11.624346	13.676376
C	0.471871	9.614515	14.368750
C	-1.032355	9.874218	14.360382
H	-1.541366	8.918055	14.593615
C	-1.425489	10.923024	15.423087
H	-1.035209	10.667443	16.429611
H	-2.529213	11.019773	15.492992
H	-1.016227	11.921328	15.160266
C	-1.529391	10.320567	12.968209
H	-1.148382	11.330194	12.706852
H	-2.638368	10.370264	12.954472
H	-1.202812	9.618629	12.178455
C	3.091047	6.790611	14.989242
H	2.381459	6.242044	15.639750
C	3.296007	5.915378	13.740742
H	2.327748	5.693053	13.255552
H	3.784366	4.955899	14.014046
H	3.918800	6.426822	12.977871
C	4.397333	6.949095	15.790873
H	5.218277	7.358520	15.166010
H	4.736786	5.960458	16.163778
H	4.266982	7.623186	16.663318
C	-1.108026	5.685345	11.694474
C	-1.794354	6.938585	12.234955
H	-1.036344	5.563135	10.600826
H	-1.256408	4.738753	12.239096
H	-2.405300	6.835828	13.148705
H	-2.199143	7.668001	11.514435
C	-0.295069	6.788041	12.300460
C	0.707562	7.568554	11.714019
H	0.718903	8.647822	11.934975
C	1.873488	7.066853	11.026756
C	2.071524	5.696446	10.662760
C	2.947543	7.947017	10.694807
C	3.250988	5.259781	10.052220
H	1.291225	4.957831	10.901646
C	4.124843	7.498119	10.080399

H	2.854690	9.012214	10.967411
C	4.322131	6.137720	9.744107
H	3.344029	4.186528	9.813101
H	4.911962	8.239015	9.872212
C	5.611317	5.595861	9.095538
C	5.272273	4.930115	7.736687
H	6.188648	4.524307	7.255077
H	4.557006	4.090757	7.858429
H	4.813475	5.665522	7.042965
C	6.647937	6.710107	8.839666
H	6.957887	7.208275	9.782111
H	7.559622	6.281629	8.372490
H	6.253950	7.488417	8.153193
C	6.256425	4.542477	10.033782
H	7.189368	4.132715	9.588563
H	6.510948	4.995381	11.015165
H	5.571956	3.690291	10.224289

TS-S1E

SCF (BP86-D3) = -1221.761226

E(SCF)+ZPE(0 K)= -1221.145743

H(298 K)= -1221.112545

G(298 K)= -1221.205393

Lowest Frequency = -177.0125 cm-1

N	0.203614	7.222186	15.150620
C	-0.127758	6.285570	14.303841
C	-0.683709	5.087710	15.075709
C	-0.383233	5.441525	16.571130
H	0.568662	4.963091	16.882795
H	-1.173198	5.082018	17.259376
C	-0.228593	6.971692	16.626384
C	-1.569693	7.678559	16.887956
H	-1.451070	8.776430	16.917426
H	-1.959279	7.356311	17.874867
H	-2.328047	7.429168	16.121562
C	0.812882	7.474149	17.628698
H	1.785701	6.958760	17.524689
H	0.439105	7.289156	18.656065
H	0.980049	8.564506	17.517400
C	-2.191328	4.841370	14.832516
H	-2.811626	5.706014	15.139726
H	-2.516806	3.962703	15.426640
H	-2.405197	4.629348	13.767458
C	0.116957	3.837439	14.638729
H	-0.069121	3.588555	13.575164
H	-0.181200	2.967333	15.261552
H	1.207504	3.994871	14.762029
C	1.049681	8.353312	14.793185
C	2.450287	8.106795	14.788424
C	3.297745	9.160641	14.396914
H	4.383392	8.989825	14.348908
C	2.782058	10.418976	14.056819
H	3.462899	11.230217	13.754538
C	1.400066	10.639537	14.092563
H	1.002129	11.626680	13.810320
C	0.499877	9.611305	14.445430
C	-0.998724	9.888083	14.359817
H	-1.529416	8.933861	14.548737
C	-1.439255	10.924496	15.416006
H	-1.108234	10.648966	16.438310
H	-2.544084	11.032624	15.426184
H	-1.005601	11.922028	15.192358
C	-1.412649	10.362478	12.949790
H	-1.012789	11.374636	12.729503
H	-2.518720	10.418438	12.872838
H	-1.043996	9.673445	12.167292
C	3.049536	6.744291	15.144155
H	2.305084	6.194284	15.753250
C	3.288033	5.895252	13.883371
H	2.335866	5.705192	13.354078
H	3.746170	4.919518	14.151361
H	3.951884	6.413264	13.160836
C	4.325193	6.861333	15.999813
H	5.177435	7.269979	15.417800
H	4.632660	5.858645	16.362699
H	4.171278	7.517602	16.881961
C	-0.980861	5.752824	11.621252
C	-1.668546	7.011285	12.148701
H	-0.862351	5.643486	10.530146
H	-1.168905	4.802008	12.145637
H	-2.322008	6.908733	13.032623
H	-2.027319	7.756482	11.419855
C	-0.177848	6.831486	12.278757
C	0.870874	7.597302	11.761826
H	0.894213	8.672222	12.003383
C	2.044489	7.086911	11.092514

C	2.225064	5.716275	10.716434
C	3.129171	7.970208	10.788058
C	3.399943	5.273505	10.094707
H	1.433827	4.987594	10.949071
C	4.300652	7.521772	10.164745
H	3.037473	9.030007	11.081381
C	4.455095	6.165135	9.808414
H	3.499404	4.206683	9.830439
H	5.112898	8.239538	9.958025
H	5.377389	5.810532	9.321612

TS-S1Z

SCF (BP86-D3) = -1221.758396

E(SCF)+ZPE(0 K)= -1221.142590

H(298 K)= -1221.109438

G(298 K)= -1221.203460

Lowest Frequency = -188.6164 cm-1

N	1.055041	7.118895	15.320922
C	1.222070	6.030257	14.582817
C	1.130776	4.819518	15.522579
C	1.279869	5.415177	16.949573
H	2.344886	5.351968	17.255043
H	0.684348	4.867021	17.707381
C	0.864466	6.890913	16.857864
C	-0.603020	7.141801	17.238590
H	-0.899068	8.190042	17.032765
H	-0.737066	6.954335	18.323314
H	-1.291970	6.473054	16.691724
C	1.763070	7.791916	17.703121
H	2.827669	7.699928	17.414076
H	1.669540	7.492670	18.767723
H	1.455513	8.852455	17.627351
C	-0.283472	4.206685	15.295632
H	-1.103099	4.917574	15.514062
H	-0.415461	3.327053	15.960283
H	-0.398061	3.871987	14.244801
C	2.149608	3.689868	15.300113
H	2.058620	3.260131	14.284111
H	1.957605	2.878575	16.035114
H	3.188895	4.042481	15.438991
C	0.626855	8.415560	14.802239
C	1.418951	9.589485	14.983394
C	0.822146	10.844408	14.732403
H	1.419542	11.757027	14.883587
C	-0.492970	10.957504	14.269702
H	-0.939336	11.949150	14.094876
C	-1.216346	9.793634	13.985061
H	-2.228064	9.875689	13.557534
C	-0.681667	8.512049	14.229666
C	-1.509695	7.307437	13.788481
H	-0.952507	6.396082	14.076877
C	-2.912324	7.255033	14.423312
H	-2.871504	7.304418	15.529125
H	-3.426115	6.312453	14.140196
H	-3.551323	8.094443	14.075298
C	-1.610927	7.281913	12.247372
H	-2.210523	8.136518	11.867435
H	-2.102317	6.346348	11.906073
H	-0.608324	7.336247	11.779870
C	2.924958	9.577817	15.253500
H	3.229282	8.534124	15.469843
C	3.655017	10.028702	13.964228
H	3.373845	9.406906	13.093003
H	4.755534	9.971502	14.094260
H	3.403194	11.081366	13.717868
C	3.382293	10.478463	16.419241
H	3.229922	11.552987	16.184501
H	4.467666	10.334978	16.601656
H	2.848814	10.264378	17.364459
C	2.367473	7.130320	12.082437
C	1.787924	5.775491	11.637450
H	1.672670	7.848095	12.548027
H	3.156524	7.589501	11.460257
H	2.169958	5.320247	10.706359
H	0.718911	5.603855	11.839522
C	2.710741	5.870642	12.794629
C	3.818023	5.154480	13.186383
H	3.792104	4.066909	12.997343
C	4.944447	5.618578	13.981044
C	5.881056	4.688666	14.516895
C	5.149621	6.992292	14.281147
C	6.949887	5.112432	15.321659
H	5.751997	3.615003	14.298875
C	6.213086	7.414942	15.088151
H	4.454797	7.721580	13.851622
C	7.122891	6.478136	15.621180

H	7.655672	4.367870	15.725219
H	6.338373	8.489267	15.304069
H	7.958505	6.809209	16.257803

TS-S2-2

SCF (BP86-D3) = -1558.957435

E(SCF)+ZPE(0 K)= -1558.336912

H(298 K)= -1558.300111

G(298 K)= -1558.403453

Lowest Frequency = -384.2929 cm-1

N	0.821607	7.141155	15.095849
C	1.711188	6.239469	14.583695
C	2.160751	5.245427	15.681058
C	1.423545	5.805898	16.931384
H	2.124838	6.438809	17.513933
H	1.077480	4.996984	17.604234
C	0.261424	6.689821	16.434850
C	-1.025097	5.851528	16.280153
H	-1.900976	6.466781	16.011198
H	-1.251844	5.357807	17.246978
H	-0.905437	5.056260	15.519053
C	0.007866	7.890352	17.350251
H	0.923211	8.498877	17.481268
H	-0.314370	7.526902	18.346631
H	-0.787240	8.549561	16.949935
C	1.745184	3.802353	15.314981
H	0.650795	3.699669	15.184840
H	2.066072	3.102398	16.114056
H	2.238484	3.500121	14.369312
C	3.680731	5.232521	15.999619
H	4.255391	4.643360	15.261817
H	3.824733	4.761180	16.995204
H	4.108901	6.254251	16.045950
C	0.665307	8.494087	14.609150
C	1.759678	9.394733	14.776453
C	1.622200	10.711949	14.293406
H	2.462076	11.414854	14.408767
C	0.437622	11.149323	13.687613
H	0.350052	12.185108	13.323836
C	-0.635900	10.260621	13.550637
H	-1.566523	10.607238	13.074683
C	-0.548015	8.924565	13.992936
C	-1.752976	8.010788	13.774863
H	-1.444017	6.984825	14.054323
C	-2.931498	8.427416	14.681570
H	-2.633711	8.497164	15.747341
H	-3.766627	7.700039	14.603307
H	-3.322216	9.423414	14.384529
C	-2.214616	7.957740	12.302649
H	-2.597854	8.940352	11.956897
H	-3.039607	7.224007	12.189613
H	-1.396030	7.654952	11.620235
C	3.062736	9.012005	15.474572
H	2.940323	7.990515	15.882450
C	4.246487	8.978038	14.486243
H	4.049494	8.286658	13.642039
H	5.170340	8.636915	14.999789
H	4.446583	9.988284	14.070134
C	3.366456	9.936473	16.672302
H	3.587323	10.973911	16.344696
H	4.255723	9.567738	17.224922
H	2.515518	9.984138	17.383021
C	1.183763	6.649656	12.169856
C	0.724513	5.351418	12.756303
H	0.569745	7.534961	12.383705
H	1.559708	6.651063	11.136186
H	1.043335	4.406057	12.292651
H	-0.201626	5.313004	13.351639
C	2.214644	6.267532	13.200272
C	3.542011	5.835037	12.889194
C	4.122722	5.390335	11.659642
C	3.420783	4.992603	10.466135
C	5.559424	5.273124	11.587572
C	4.088918	4.568364	9.315573
H	2.322215	4.983200	10.443083
C	6.223979	4.850179	10.442221
H	6.150224	5.544920	12.478441
C	5.499477	4.502696	9.272564
H	3.505198	4.272399	8.429973
H	7.323919	4.791323	10.437549
H	4.240371	5.864263	13.731926
C	6.215010	3.996101	8.067266
F	5.484156	4.125781	6.912298
F	6.538762	2.646256	8.146481
F	7.416340	4.633443	7.850329

TS-S2-3

SCF (BP86-D3) = -1378.933782

E(SCF)+ZPE(0 K)= -1378.208918

H(298 K)= -1378.170333

G(298 K)= -1378.275294

Lowest Frequency = -414.7875 cm-1

N	0.820748	7.151666	15.113409
C	1.745349	6.269726	14.618164
C	2.224164	5.307795	15.727377
C	1.459311	5.855534	16.967206
H	2.135150	6.516203	17.549204
H	1.134760	5.042215	17.645752
C	0.271476	6.696675	16.454323
C	-0.985161	5.813167	16.303717
H	-1.879829	6.394542	16.021220
H	-1.201783	5.322316	17.274536
H	-0.831971	5.014798	15.551801
C	-0.024372	7.897148	17.357413
H	0.870843	8.535960	17.484746
H	-0.338825	7.534247	18.356588
H	-0.838081	8.527126	16.947454
C	1.864702	3.845229	15.377185
H	0.774485	3.698553	15.252634
H	2.215906	3.164806	16.180616
H	2.366392	3.555661	14.431770
C	3.742357	5.353989	16.053100
H	4.338464	4.780270	15.319968
H	3.903675	4.899910	17.054597
H	4.131880	6.391520	16.085911
C	0.632555	8.494064	14.614668
C	1.703206	9.425522	14.770303
C	1.538022	10.729888	14.262658
H	2.360802	11.454207	14.369370
C	0.345225	11.129700	13.645991
H	0.234699	12.156622	13.263645
C	-0.707448	10.214012	13.524085
H	-1.645167	10.530837	13.041058
C	-0.589837	8.888044	13.988999
C	-1.772819	7.942898	13.783902
H	-1.434720	6.926730	14.066304
C	-2.956068	8.334175	14.695770
H	-2.654515	8.414122	15.759692
H	-3.774887	7.587567	14.624480
H	-3.370829	9.320217	14.397662
C	-2.244095	7.869699	12.315529
H	-2.660886	8.838333	11.968818
H	-3.046674	7.110089	12.211420
H	-1.421045	7.590040	11.628568
C	3.007426	9.085956	15.487748
H	2.894994	8.078052	15.930533
C	4.196396	9.031538	14.507100
H	4.016681	8.300366	13.692253
H	5.124788	8.731835	15.038369
H	4.377798	10.026715	14.047847
C	3.291546	10.054981	16.654569
H	3.506224	11.082655	16.293506
H	4.179231	9.714290	17.227558
H	2.433448	10.118813	17.355571
C	1.189145	6.630390	12.184379
C	0.808337	5.320093	12.790268
H	0.541951	7.491148	12.397091
H	1.577435	6.636132	11.155363
H	1.147443	4.385977	12.317767
H	-0.104260	5.244572	13.403341
C	2.227158	6.268434	13.224610
C	3.567001	5.874105	12.906115
C	4.138495	5.385521	11.679993
C	3.434176	4.946593	10.506781
C	5.567080	5.271349	11.584258
C	4.101525	4.486791	9.364208
H	2.334697	4.935648	10.485411
C	6.220036	4.808279	10.437942
H	6.174195	5.572483	12.455824
C	5.512112	4.406005	9.277793
H	3.485720	4.167786	8.505903
H	7.319813	4.764399	10.457674
H	4.276170	5.944800	13.737901
C	6.191607	3.899259	7.990459
C	5.802521	4.817528	6.802654
H	6.276559	4.467873	5.859498
H	4.704906	4.834469	6.641136
H	6.131541	5.861401	6.988976
C	5.724034	2.451363	7.689504
H	6.197676	2.067136	6.759716
H	5.995236	1.769875	8.522983
H	4.624206	2.396068	7.553530

C	7.730332	3.892886	8.109309
H	8.132188	4.909889	8.300485
H	8.077375	3.226914	8.926845
H	8.183247	3.526033	7.164166

TS-S2E

SCF (BP86-D3) = -1221.786278

E(SCF)+ZPE(0 K)= -1221.169959

H(298 K)= -1221.137011

G(298 K)= -1221.229466

Lowest Frequency = -410.0583 cm-1

N	0.823967	7.148313	15.092746
C	1.733783	6.259497	14.583986
C	2.189085	5.266884	15.676413
C	1.428649	5.804413	16.923364
H	2.113919	6.440915	17.521038
H	1.085840	4.984150	17.584314
C	0.259315	6.677469	16.421977
C	-1.012898	5.820922	16.248371
H	-1.895143	6.424338	15.973204
H	-1.243311	5.315776	17.208595
H	-0.871142	5.034247	15.482037
C	-0.018564	7.865542	17.346897
H	0.887691	8.485011	17.490593
H	-0.344306	7.489152	18.337423
H	-0.818629	8.518297	16.945805
C	1.804025	3.818797	15.295086
H	0.712029	3.695573	15.161277
H	2.137268	3.116159	16.086927
H	2.305950	3.538378	14.347060
C	3.706118	5.278292	16.010506
H	4.295850	4.707864	15.269758
H	3.852890	4.801766	17.003722
H	4.114706	6.307492	16.066116
C	0.661411	8.503314	14.619077
C	1.747288	9.412541	14.798346
C	1.606563	10.729811	14.317241
H	2.441144	11.437490	14.442210
C	0.423637	11.162585	13.703973
H	0.332410	12.198940	13.342577
C	-0.643811	10.267745	13.558250
H	-1.573636	10.610378	13.077623
C	-0.550960	8.930663	13.996181
C	-1.748558	8.010167	13.765895
H	-1.429834	6.983279	14.031364
C	-2.930924	8.404615	14.677516
H	-2.634864	8.460146	15.744545
H	-3.761485	7.673171	14.587563
H	-3.327493	9.402651	14.395006
C	-2.210940	7.972579	12.293371
H	-2.607685	8.954898	11.961990
H	-3.026389	7.229761	12.169989
H	-1.388438	7.690628	11.606716
C	3.042960	9.035684	15.512630
H	2.912258	8.020417	15.932993
C	4.234525	8.982763	14.534977
H	4.045630	8.272938	13.703550
H	5.155553	8.655404	15.062688
H	4.434704	9.984624	14.098670
C	3.338897	9.974260	16.701201
H	3.571562	11.005883	16.363297
H	4.218906	9.606653	17.269355
H	2.479579	10.036914	17.400799
C	1.200288	6.681081	12.156489
C	0.786034	5.367485	12.733560
H	0.570426	7.551384	12.382427
H	1.593542	6.698602	11.129503
H	1.108811	4.435500	12.245718
H	-0.131981	5.300356	13.339352
C	2.224973	6.278626	13.194174
C	3.558785	5.863500	12.876886
C	4.131994	5.389639	11.646117
C	3.423287	4.986903	10.461020
C	5.564311	5.256472	11.570383
C	4.090496	4.535724	9.311602
H	2.323990	4.997902	10.437905
C	6.220012	4.803410	10.423303
H	6.160166	5.533683	12.457367
C	5.494350	4.441168	9.265691
H	3.492543	4.240214	8.432453
H	7.321025	4.730529	10.426347
H	6.010460	4.086452	8.359945
H	4.262181	5.905284	13.715367

TS-S2Z

SCF (BP86-D3) = -1221.780121

E(SCF)+ZPE(0 K)= -1221.163520

H(298 K)= -1221.130930

G(298 K)= -1221.221346

Lowest Frequency = -479.7375 cm-1

N	0.943558	7.141084	15.220662
C	1.647616	6.152653	14.591513
C	1.536206	4.871436	15.457699
C	1.383867	5.484307	16.870725
H	2.388085	5.651954	17.310420
H	0.833300	4.815867	17.561154
C	0.659113	6.841892	16.689898
C	-0.839147	6.779569	17.017781
H	-1.317795	7.758751	16.824833
H	-0.964076	6.552145	18.095519
H	-1.374722	6.002207	16.443349
C	1.301685	7.929109	17.566176
H	2.392194	7.994312	17.393963
H	1.140141	7.673820	18.633207
H	0.850829	8.924433	17.378536
C	0.227780	4.110921	15.078915
H	-0.639179	4.787684	14.948400
H	-0.023723	3.388312	15.882970
H	0.360656	3.542768	14.138569
C	2.682240	3.854838	15.357651
H	2.831900	3.546981	14.304376
H	2.421565	2.954474	15.952817
H	3.640903	4.253756	15.734832
C	0.617428	8.439948	14.663614
C	1.590965	9.482735	14.642036
C	1.193471	10.759958	14.193795
H	1.935755	11.573309	14.180100
C	-0.114367	11.012779	13.763574
H	-0.404982	12.022039	13.431380
C	-1.042584	9.965187	13.731440
H	-2.058204	10.155144	13.350286
C	-0.700210	8.663883	14.155633
C	-1.731337	7.553125	13.941008
H	-1.307592	6.617603	14.357268
C	-3.080562	7.828023	14.637695
H	-2.963603	8.053448	15.714971
H	-3.749304	6.947225	14.542628
H	-3.603411	8.689427	14.171504
C	-1.979640	7.324010	12.431684
H	-2.473263	8.206539	11.973301
H	-2.645819	6.449267	12.278219
H	-1.041675	7.142442	11.873027
C	3.055374	9.275563	15.011253
H	3.169818	8.242961	15.384685
C	3.962326	9.400064	13.767972
H	3.633330	8.722377	12.958015
H	5.000960	9.113699	14.033627
H	3.973354	10.441562	13.380814
C	3.544005	10.232657	16.117715
H	3.599395	11.280968	15.754852
H	4.564488	9.939649	16.441723
H	2.883499	10.221537	17.007242
C	1.699605	6.857814	12.166735
C	1.098649	5.517572	12.461272
H	1.035948	7.728227	12.302940
H	2.323747	6.957451	11.260783
H	1.536164	4.618634	12.002411
H	0.041143	5.442546	12.759377
C	2.457523	6.358632	13.369278
C	3.821224	5.958316	13.255023
H	4.149741	5.760652	12.218809
C	4.880384	6.009840	14.223466
C	6.230418	5.770946	13.789693
C	4.746074	6.370406	15.607252
C	7.329896	5.919584	14.641775
H	6.398785	5.477380	12.739310
C	5.850887	6.532869	16.452800
H	3.744677	6.528728	16.028724
C	7.163240	6.311409	15.988517
H	8.342498	5.726311	14.248624
H	5.678826	6.825386	17.502735
H	8.029452	6.425332	16.658528

10) References

[S1] Müller, C., Andrada, D. M., Bischoff, I. A., Zimmer, M., Huch, V., Steinbrück, N., Schäfer, A., *Organometallics*, **2019**, *38*, 1052−1061.

[S2] Lavallo, V., Canac, Y., Präsang, C., Donnadieu, B., Bertrand, G., *Angew. Chem. Int. Ed.,* **2005**, *44*, 5705–5709.

[S3] Driess, M., Yao, S., Brym, M., Wüllen, C., Lentz, D., *J. Am. Chem. Soc.*, **2006**, *128*, 9628−9626.

[S4] Driess, M., Yao, S., Brym, M., Wüllen, C., Lentz, D., *Angew. Chem. Int. Ed.*, **2006**, *45*, 4349−4352.

[S5] Jana, A., Objartel, I., Roesky, H. W., Stalke. D., *Inorg. Chem.,* **2009**, *48*, 798−800.

[S6] Gans-Eichler, T., Gudat, D., Nieger, M., *Angew. Chem. Int. Ed.*, **2002**, *41*, 1888−1891.

[S7] Palani, A., Wendlandt, A. E., *J. Am. Chem. Soc.*, **2023**, *145*, 20053−20061.

[S8] Kong, R. Y., Crimmin, M. R., *J. Am. Chem. Soc.*, **2020**, *142*, 11967−11971.

[S9] Agilent (2014). *CrysAlis PRO*. Agilent Technologies Ltd, Yarnton, Oxfordshire, England.

[S10] Dolomanov, O. V., Bourhis, L. J., Gildea, R. J., Howard, J. A. K., Puschmann, H., OLEX2 : a complete structure solution, refinement and analysis program *J. Appl. Crystallogr.* **2009**, *42*, 339–341.

[S11] Sheldrick, G. M., SHELXT – Integrated space-group and crystal-structure determination *Acta Crystallogr. Sect. A Found. Adv.* **2015**, *71*, 3–8.

[S12] Sheldrick, G. M., Crystal structure refinement with SHELXL *Acta Crystallogr. Sect. C Struct. Chem.* **2015**, *71*, 3–8.

[S13] Gaussian 16, Revision C.01, M. J. Frisch, G. W. Trucks, H. B. Schlegel, G. E. Scuseria, M. A. Robb, J. R. Cheeseman, G. Scalmani, V. Barone, G. A. Petersson, H. Nakatsuji, X. Li, M. Caricato, A. V. Marenich, J. Bloino, B. G. Janesko, R. Gomperts, B. Mennucci, H. P. Hratchian, J. V. Or z, A. F. Izmaylov, J. L. Sonnenberg, D. Williams-Young, F. Ding, F. Lipparini, F. Egidi, J. Goings, B. Peng, S273 A. Petrone, T. Henderson, D. Ranasinghe, V. G. Zakrzewski, J. Gao, N. Rega, G. Zheng, W. Liang, M. Hada, M. Ehara, K. Toyota, R. Fukuda, J. Hasegawa, M. Ishida, T. Nakajima, Y. Honda, O. Kitao, H. Nakai, T. Vreven, K. Throssell, J. A. Montgomery, Jr., J. E. Peralta, F. Ogliaro, M. J. Bearpark, J. J. Heyd, E. N. Brothers, K. N. Kudin, V. N. Staroverov, T. A. Keith, R. Kobayashi, J. Normand, K. Raghavachari, A. P. Rendell, J. C. Burant, S. S. Iyengar, J. Tomasi, M. Cossi, J. M. Millam, M. Klene, C. Adamo, R. Cammi, J. W. Ochterski, R. L. Mar n, K. Morokuma, O. Farkas, J. B. Foresman, and D. J. Fox, Gaussian, Inc., Wallingford CT, 2016.

[S14] Becke, A. D. *Phys. Rev. A*, **1988**, 38, 3098.

[S15] Perdew, J. P. *Phys. Rev. B*, **1986**, 33, 8822.

[S16] Becke, A. D., *J. Chem. Phys.*, **1993**, 5648–5652.

[S17] Hehre, W. J., Ditchfield, R., Pople., J. A., *J. Chem. Phys.*, **1972**, *56*, 2257-2261.

[S18] Hariharan, P. C., Pople., J. A., *Theoret. Chim. Acta,* **1973**, *28*, 213-222

[S19] Grimme, S., Antony, J., Ehrlich, S., Krieg, H., *J. Chem. Phys.*, **2010**, *132*, 154104-154119.

[S20] Grimme, S., Ehrlich S., Goerigk, L., *J. Comput. Chem.*, **2011**, *32*, 1456-1465.

[S21] Ishida, K., Morokuma, K., Komornicki, A., *J. Chem. Phys.*, **1977**, 66 (5), 2153–2156.

[S22] Luchini, G., Alegre-Requena, J. V., Funes-Ardoiz, I., Paton, R. S., *F1000Research*, **2020**, *9*, 291.

[S23] Glendening, E. D., Landis, C. R., Weinhold, F., *J. Comput. Chem.*, **2019**, *40*, 2234-2241.

[S24] Luchini, G.; Alegre-Requena, J. V.; Funes-Ardoiz, I.; Paton, R. S. GoodVibes: Automated Thermochemistry for Heterogeneous Computational Chemistry Data. *F1000Research*, **2020**, *9*, 291.
